# Novel SRY-box transcription factor 9 variant in campomelic dysplasia and the location of missense and nonsense variants along the protein domains: A case report

**DOI:** 10.3389/fped.2022.975947

**Published:** 2022-11-18

**Authors:** Carlos A. Calvache, Estefanía C. Vásquez, Vanessa I. Romero, Kazuyoshi Hosomichi, Juan C. Pozo

**Affiliations:** ^1^ School of Medicine, Universidad San Francisco de Quito, Quito, Ecuador; ^2^ Department of Bioinformatics and Genomics, Kanazawa University, Kanazawa, Japan; ^3^ School of Medicine, Universidad de Cuenca, Cuenca, Ecuador

**Keywords:** campomelic dysplasia, high-mobility group box (HMG), self-dimerization domain (DIM), SOX9 gene, Ecuador

## Abstract

**Background:**

Campomelic dysplasia (CD) is a rare disorder that involves the skeletal and genital systems. This condition has been associated with a diverse set of mutations in the SRY-box transcription factor 9 (*SOX9*) gene.

**Case presentation:**

We herein report a case involving a 4-year-old female patient with CD, female sex reversal, type 1 Arnold–Chiari malformation, and bilateral conductive hearing loss and investigate the causal mutation. Whole-exome sequencing analysis detected a novel Trp115X* variant in the *SOX9* gene. We performed a literature review of the reported cases and demonstrated that the missense variants were located only in the self-dimerization domain (DIM) and high-mobility group box domains. We also reported that variants in the DIM domain do not cause sex reversal and identified that the amino acid sequences that were mutated in the patients with campomelic dysplasia are evolutionarily conserved among primates.

**Conclusions:**

We suggest that missense variants cannot be located in the K2, PQA, and PQS given that these domains function critically for transcriptional activation or repression of target genes and evolve under purifying selection.

## Background

Campomelic dysplasia (CD) belongs to the group of almost lethal prenatal skeletal dysplasia, causing abnormalities during bone and sexual development and heart, respiratory, and neurological malformations (1). Mutations in the SRY-box transcription factor 9 (*SOX9*) gene have been associated with CD. We herein report a novel nonsense variant in a child with CD, sex reversal, and Arnold–Chiari malformation type I and discovered that missense variants were located in the self-dimerization domain (DIM) and high-mobility group box (HMG) domains. Further, we found that no sex reversal when variants were located in the DIM domain, and that the missense variants were limited to humans.

CD is an autosomal dominant skeletal dysplasia that is primarily and clinically characterized by micrognathia, cleft palate, small chest, shortened long-type bones, scapular hypoplasia, clubfoot, and complete sex reversal or ambiguous genitalia. In fact, around one-third of affected men show complete sexual feminization or a degree of ambiguous genitalia. This condition is usually fatal in the perinatal period and has been associated with high postnatal mortality rates due to respiratory failure (2), with mortality rates reaching 77% in the first month and 90% by the second year (3). The main clinical differential diagnosis includes osteogenesis imperfecta, kyphomelic syndrome, thanatophoric dysplasia, mesomelic dysplasia, and Cummings syndrome (3).

*SOX* gene is the only causal gene for CD. The protein produced by this gene is a transcription factor involved in chondrogenesis that promotes cartilage-specific extracellular matrix components, regulates other SOX family genes, and plays a key role in the development of the endochondral skeleton and male sexual differentiation (4). *SOX9* domains include an HMG domain, a DIM domain, and three transactivation domains (TAD), namely K2, PQA, and PQS. The HMG box creates a twisted L-shaped structure that binds to the minor groove of the DNA. DIM has a dimerization function and directly influences the transactivation activity. The DIM and TAD domains function in the transcriptional regulation of pathways involving *SOX9*. TADs control the transcriptional machinery by mediating protein–protein interactions. K2 and PQS are critical for the appropriate transcriptional activation or repression of target genes. PQA, which is exclusive to mammals, enhances PQS transactivation activity, is unable to activate transcription alone, and is associated with the SRY sex-determining mechanism (5). Most pathological variants are *de novo*; however, there are a few parental germline mosaic cases (2).

This report described a case involving a female patient with skeletal dysplasia in whom whole-exome sequencing (WES) in trio analysis detected a novel nonsense variant, reviewed published cases, determined the locations of the reported variants along the gene domains, and compared the variants with other primates.

## Case presentation

**Figure 1:**
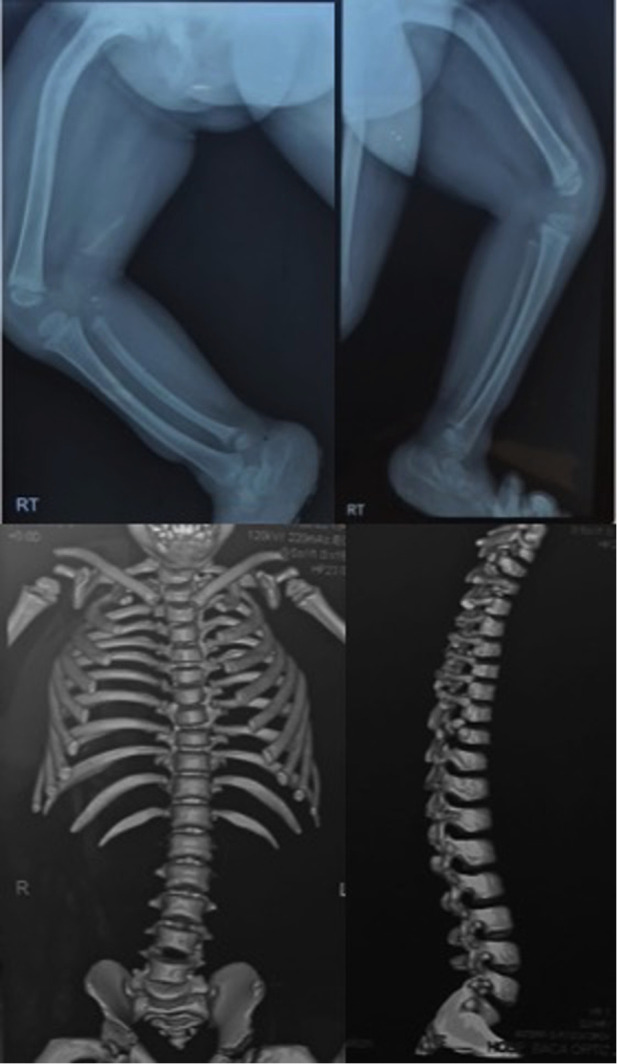
Radiography and computed tomography of the legs, hip, and spine showing femoral, tibial, and fibular bowing, hip rotation, and an abnormal spine curvature.

Our case involves a 4-year-old patient assigned as female at birth who visited the genetic outpatient clinic for ambiguous genitalia and skeletal dysplasia. She was born *via* cesarean delivery from non-consanguineous parents and had a cleft palate, which was corrected at 4 months. The girl had a moderate developmental delay characterized by being able to stand at 1 year of age and walk at 3 years of age after constant physiotherapy. Physical examination of the head revealed trigonocephaly, a protruding frontal region, almond-shaped eyes with antimongoloid deviation, a flat nasal bridge, a long philtrum, retromicrognathia, and a short neck. Radiography of the head revealed a vertebral fusion between C5 and C6 (Figure 1). We observed limb malformations, including prominent hips, shortening of the right lower limb, valgus knees, and rhizomelic arms (Table 1). Radiography of the limbs revealed femur bowing, an underdeveloped acetabulum, and a femoral head. At birth, the physicians described the appearance of external female genitalia. Abdominal ultrasonography revealed ovaries, a small uterus, and shortened vagina. Hormone analysis showed increased testosterone, luteinizing hormone, and follicle-stimulating hormone levels. A peripheral blood karyotype reported a 46XY with a positive SRY gene. Brain magnetic resonance imaging showed Arnold–Chiari malformation type 1 (Supplementary Figure S1). Moreover, she had moderate conductive bilateral hearing loss, which was corrected with hearing aids (Supplementary Figure S2). Written informed consent was obtained from the parents to perform WES given that this analysis is not covered by the public health system.

**Table 1:** Clinical features of the nonsense and missense variants in reported cases.

Nonsense variants																				
		E28X		59X (C155bp)	59X (C155bp)	W86X	261-262insG	W115X	Q117X	Y319X	Q375X	Q391X	R394X	E400X	Q412X	Ser438X	Y440X	Y440X	Y440X	Q458X
**General Features**																				
‘Intellectual disability								X	X									X		
Short stature					X			X					X		X					X
**Facial Features**																				
Flat face					X		X				X			X	X	X		X	X	X
Macrocephaly				X										X				X		
Depressed nasal bridge				X	X		X	X								X	X	X	X	
Hypertelorism																X				
Low-set ears						X	X				X									
Long philtrum					X															
Cleft palate		X					X				X			X				X	X	
Micrognathia				X	X			X		X				X		X	X	X	X	
Retrognathia		X														X	X	X		
**Bone malformation**																				
Spinal dysraphism											X	X							X	X
Hypoplastic scapula		X		X	X	X				X	X			X			X			
Bell Shaped Thorax																	X		X	
Bowing of the bones		X		X	X	X	X	X	X	X	X	X	X	X		X	X	X	X	
Dislocation of radial head/hip				X												X				
Hypoplastic patellae																				
**Respiratory**																				
Tracheobronchomalacia																X			X	
Hypoplastic Lungs																				
Respiratory Distress				X		X	X				X			X		X	X			
**Neurological**																				
Abnormal Brain Convolutions					X															
Hypoplastic of Corpus Collosum								X												
Ventriculomegaly								X												
Absence of the Olfactory Bulbs																			X	
**Cardiovascular**																				
Abnormalities																				
**Renal**																				
Hydronephrosis							X												X	X
Other Renal Abnormalities							X				X									X
**Status**																				
Abortion		14 weeks								21 weeks		20 weeks	14 weeks		14 weeks					27 weeks
Death				Day 1		Day 12					Week 7			Week 10			Month 3			
Last reported age					18 years		39 weeks gestation	4 years	12 years							Newborn		11 years	4 years	
**Reproductive system**																				
Sex Reversal								X	X		X						X			
External genitalia		Male		Female	Female	Female	Male	Female	Male	Female	Female	Unknown	Female	Male	Unknown	Male	Female	Female	Male	Female
karyotype		XY		XX	XX	XX	ND	XY	XX	XX	XY	ND	XX	XY	ND	XY	XY	XX	XY	XX
**Others**																				
Hearing Impairment					X			X	X								X	X		
**Protein domain**		DIM		DIM	DIM	DIM	DIM	HMG	HMG		PQA				PQS	PQS	PQS	PQS	PQS	PQS

**Missense variants**																				
	H65Y	A76E	Q79P	V80G	P108L	F112S	M113V	119 (C358bp)	A119V	W143R	R152P	F154L	A158T	A158V	H165Q	H169Q	H169P	P170L	P170L	P170R
**General Features**																				
Intellectual disability	X			X					X									X	X	
Short stature									X					X				X	X	
**Facial Features**																				
Flat face					X		X	X	X				X				X		X	X
Macrocephaly	X									X			X	X				X		
Depressed nasal bridge	X											X		X			X	X		
Hypertelorism	X		X									X		X				X		
Low-set ears	X		X		X					X		X		X	X			X	X	X
Long philtrum														X						
Cleft palate	X	X	X	X	X		X		X				X		X	X			X	X
Micrognathia	X		X	X			X	X	X			X		X	X	X				X
Retrognathia								X									X	X		X
**Bone malformations**																				
Spinal dysraphism							X			X										
Hypoplastic scapula	X	X			X	X	X		X	X				X	X					X
Bell Shaped Thorax				X			X								X					
Bowing of the bones	X		X	X	X	X		X		X		X	X	X		X	X	X	X	X
Dislocation of radial head/hip	X			X			X								X					
Hypoplastic patellae	X																			
**Respiratory**																				
Tracheobronchomalacia	X		X	X	X		X		X											
Hypoplastic Lungs															X					
Respiratory Distress	X					X								X				X		
**Neurological**			X																	
Abnormal Brain Convolutions																				
Hypoplastic of Corpus Collosum			X																	
Ventriculomegaly																				
Absence of the Olfactory Bulbs																				
**Cardiovascular**																				
Cardiovascular Abnormalities			X	X															X	
**Renal**																				
Hydronephrosis																				
Other Renal Abnormalities								X												
**Status**																				
Abortion or death								Week 21									Week 20			
Death					Month 6					Month 5	Day 2	Day 1								Month 1
Last reported age	11 years	6 years	1.5 years	2 years 2 months		Newborn	Newborn		Newborn				19 years	Newborn	Newborn	10 years		21 years	15 years	
**Reproductive system**																				
Sex Reversal					X					X			X							
External genitalia	Male	Male	Male	Female	Female	Male	Female	ND	Male	Female	Female	Female	Female	Female	Male	Male	Male	Female	Female	Male
Karyotype	XY	XY	XY	XX	XY	XY	XX	ND	XY	XY	XX	XX	XY	XX	XY	XY	XY	XX	XX	XY
**Others**																				
Hearing Impairment			X	X														X	X	
**Protein domain**	DIM	DIM	DIM	DIM	HMG	HMG	HMG	HMG	HMG	HMG	HMG	HMG	HMG	HMG	HMG	HMG	HMG	HMG	HMG	HMG

ND, not described; Blank, not reported on the paper.

We used a capture of target regions using probes, followed by next-generation sequencing with Illumina technology. The total number of reads was 41,660,733, whereas that of the mapped reads was 41,603,016 (99.86%). We aligned the raw data using the Burrows–Wheeler Aligner software, sorted and merged the data using the Picard tools software, and identified the nucleotide variants (SNV) and insertions or deletions (indel) using GATK. The GRCh37 version of the human genome was taken as reference.

After performing WES on the mother, father, and patient, we identified 1,582 variants causing non-synonymous, stop-gain, and frameshift changes; selected those with minor allele frequency (<0.01) in various databases; filtered variants unique to the daughter and absent in the parents; and detected one nonsense heterozygote variant in the *SOX9* gene of the patient. We sequenced only the *SOX9* gene and confirmed the variant. The pathogenic variant was caused by a guanine to adenine change at the 344 bp (c.G344A), resulting in a tryptophan to a premature stop codon change at position 115 (p.Trp115*) of the HMG domain. The frequency of this variant was 0 in the Genome Aggregation Database and Exome Aggregation Consortium databases and was not reported in the Ensembl genome and ClinVar databases. The parents did not have the nonsense variant and were homozygous for the reference allele (G). To the best of our knowledge, no genes have been previously associated with Arnold–Chiari malformation type I.

**Figure 2:**
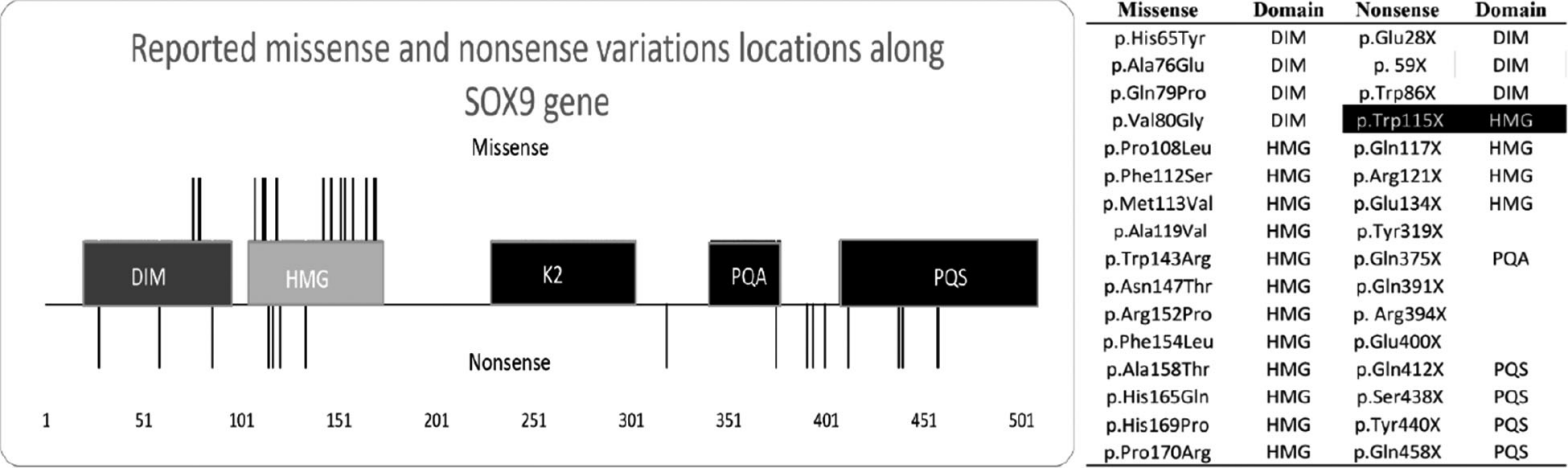
Graphic description of the *SOX* gene variant. We identified 115 cases of campomelic dysplasia, determined the amino acid variant (right side), and constructed a visual representation of the variant along *SOX9* (left side). Missense variants are the lines over the image, whereas nonsense variants are the lines below the image. Missense variants are limited from amino acids 76 to 170. Ala = alanine, arg = arginine, asn = asparagine, asp = aspartic acid, asx = asparagine or aspartic acid, cys = cysteine, glu = glutamic acid, gln = glutamine, glx = glutamine or glutamic acid, gly = glycine, his = histidine, ile = isoleucine, leu = leucine, lys = lysine, met = methionine, phe = phenylalanine, pro = proline, ser = serine, thr = threonine, trp = tryptophan, tyr = tyrosine, val = valine. The *SOX9* domains include a high-mobility group box (HMG) (gray box), self-dimerization domain (DIM) (dark gray box), and three transactivation domains (TAD), namely K2, PQA, and PQS. Black box includes the reported case.

We subsequently performed a literature review on CD and found 115 reported cases. Thereafter, we filtered the cases with nonsense and missense variants and excluded translocations and papers that did not include the description of the mutation. The results after correlating the variant location with the domains along the gene are shown in Figure 2 and Supplementary Table S1. The final number of research articles included 18 nonsense and 20 missense cases. Although variants p.Asn147Thr, p.Arg121*, and p.Glu134* were mentioned in review articles, no clinical description was provided.

Our literature review (Supplementary Table S1) revealed that missense variants were located at the beginning of *SOX9*, with 4 located in the DIM and 12 in the HMG domains. There was no missense variant in the K2, PQA, and PQS domains. There were no reported cases of sex reversal with mutations in the DIM domain, but three cases with mutations in the HMG domains exhibited sex reversal. The reported missense cases shared facial features and bone malformations. Three of these patients had cardiovascular malformations. Premature death did not occur in patients with DIM mutations, unlike those with HMG mutations who died during the first months of life. The oldest reported individual was a 21-year-old woman.

Nonsense variants were located along the whole *SOX9* gene, with three in the DIM, four in the HMG, one in the PQA, and four in the PQS domain. The mutation in our patient was localized in the HMG domain. Four mutations (Tyr319*, Gln391*, Arg394*, and Glu400*) did not occur on a functional domain and could alter the protein structure. There was no nonsense variant in the K2 domain. The reported nonsense cases shared facial features and bone malformations. Our patient, along with two others, had neurological malformations. Most patients died during gestation or the postnatal period or were aborted by the mothers. The oldest reported individual was an 18-year-old woman. There were no reported cases of sex reversal with mutations in the DIM, whereas two cases with mutations in the HMG, one with mutations in the PQA, and one with mutations in the PQS domains exhibited sex reversal. From the information provided in the reported cases, respiratory and cardiovascular malformations were more common in missense than in nonsense variants.

We considered that the variant causing CD develops only in humans given that *SOX9* alters essential functions during development. There were no reported cases of primates with phenotypes such as CD. We compared and aligned a normal human sequence and all missense variants with *SOX9* from chimpanzees (*Pan troglodytes* NC_036896.1), orangutans (*Pongo abelii* NC_036920.1), gorillas (*Gorilla* NC_044619.1), rhesus monkeys (*Macaca mulatta* NC_041770.1), and house mice (*Mus musculus* NC_000083.7). The protein alignment confirmed that the *SOX* gene was highly preserved between species (similarity between 96.45% and 100%, as described in Supplementary Figure S3) and that no other species had the missense or nonsense variants (Supplementary Figures S4A,B) (6, 7).

## Discussion and conclusion

CD results from missense, nonsense, frameshift, or splice site loss-of-function mutations in one of the three *SOX9* exons (2). *SOX9* is a transcription factor during chondrogenesis that promotes cartilage-specific extracellular matrix components and regulates other SOX family members. We performed WES in trio analysis, which revealed a Trp115* novel variant, conducted a literature review of the reported cases, located the variants along *SOX9* domains, and compared the variants with other primates to determine the conservation (Supplementary Table S2).

Our patient presented with skeletal dysplasia, characteristic facial features, and sex reversal. Compared to other cases, she did not exhibit cardiovascular and respiratory abnormalities, which are the main causes of premature death (8). However, she is the first reported case of CD and Arnold–Chiari malformation type 1. The oldest reported nonsense survivor was still alive at 18 years of age (3). Although the reason for the patient's survival was not described in the publication, we believe that it may be attributed to the lack of respiratory and cardiovascular defects, such as tracheobronchomalacia and laryngomalacia (3).

Missense variants are located at the DIM and HMG domains, which are the most conserved between species. The reported missense variant was not located in the K2, PQA, and PQS, which are domains that evolve under purifying selection and sweep away deleterious mutations. The missense and nonsense variants were not between the 66–75 residues of the DIM that interacts with the promoter activation of *Amh*, which is a sex-determining gene that requires only a *SOX9* monomer for its activation. Cases with DIM mutations showed no sex reversal. The HMG box is highly conserved with a significant constraint due to its binding pattern, and mutations reduce the DNA binding affinity (9). Mutations in the DIM domain decrease the dimerization and cease or reduce the transactivation activity for chondrogenic and sex-determining genes without altering other domain functions. We suggest that the lack of missense variants in the K2, PQA, and PQS domains was associated with the critical function during transcriptional activation or repression of target genes or that a single missense variant in the K2, PQA, and PQS domains could be responsible for the CD phenotype when the DIM or HMG domains are intact (5). Moreover, the K2 and PQS domains have evolved under purifying selection and have a relaxed selective pressure (primarily K2), reflecting a higher fixation of mutations (5).

Previous studies have reported that nonsense mutations in the DIM and HMG (64 to 181 aa) present a classical type of CD with a reduced amount of *SOX9*. Cases with DIM mutations showed no sex reversal. Nonsense variants between the K2 and PQA (228 to 401 aa) have more truncated proteins, which escape the nonsense-mediated decay and have a dominant-negative effect. In contrast, the variant along the PQS (402–509 aa) produces a truncated protein but leaves a longer fraction of the TA domain, resulting in a lower transactivation effect and allowing patients to survive for several years (2). Meyer in 1997 reported 12 cases of CD and concluded no phenotype/genotype correlation, although 3 out of the 12 patients were not found to have variants in *SOX9* and gene sequencing was not available. However, not all *SOX9* domains had been identified in 1997, and Meyer suggested the need for a larger sample and research on the residual transactivation activity of a mutant *SOX9* protein. Gene sequencing can be beneficial for detecting point mutations that cause CD. Our study is the first to review the literature that analyzes all reported cases, associates these cases with the functional domains, and agrees with Meyer regarding the phenotype variability and residual activity of mutant *SOX9* (10).

One limitation of the current study was our use of information provided by the articles and not that obtained from the concerned patients. Furthermore, we did not conduct protein quantification in our patient considering that the pathology could have resulted from either a null protein or dominant-negative protein.

*SOX9*, which binds to response elements of target genes as a homodimer or monomer to activate or repress the transcriptional machinery, has been associated with CD—a rare disorder that involves the skeletal and genital systems. Our study described the first case of CD with Arnold–Chiari type malformation 1 (Trp115*) and found that the reported missense variants were located between amino acids 76–170 in the DIM and HMG domains, Further, we discovered that variants in the DIM domain had no sex reversal and that the mutated amino acid sequences in the human patients with CD were evolutionarily conserved among primates.

## Data Availability

The datasets for this article are not publicly available due to concerns regarding participant/patient anonymity. Requests to access the datasets should be directed to the corresponding author.
