# Supplementary material for: Novel SRY-box transcription factor 9 variant in campomelic dysplasia and the location of missense and nonsense variants along the protein domains: A case report
Source: Front Pediatr. 2022 Nov 18;10:975947. doi: 10.3389/fped.2022.975947 (PMC9716274; doi:10.3389/fped.2022.975947)
Supplement: Supplementary file 1 [file Datasheet1.pdf]

Supplementary data

Supplementary Figure 1. Brain and spine CT and MRI showing type 1 Arnold–Chiari malformation.

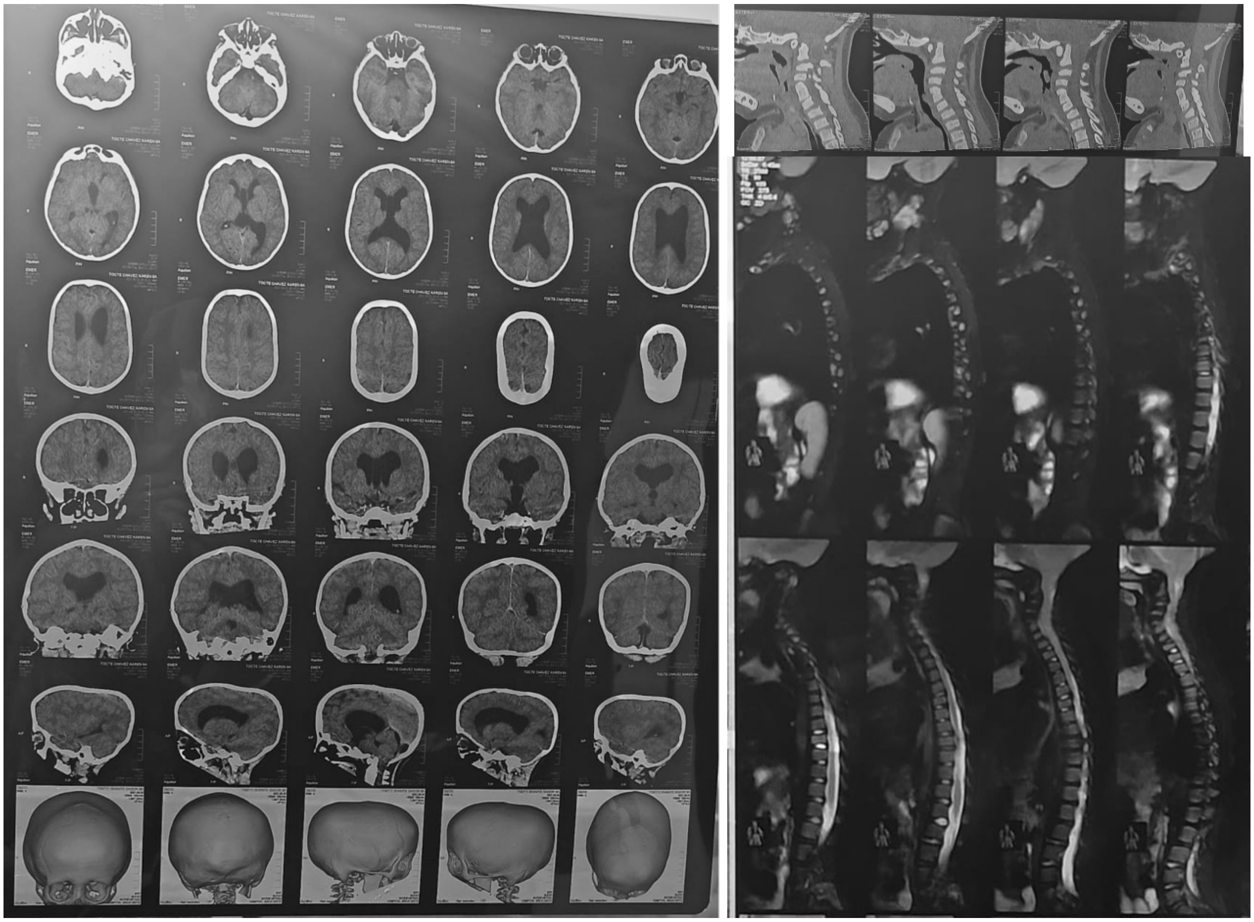

Supplementary figure 2. Timeline

Timeline

2014-03-04	C-section delivery
2014-07-10	Cleft palate correction
2015-01-13	Developmental delay
2015-04-14	Abdominal ultrasound reported ovaries, a small uterus and shortened vagina, and increased testosterone, LH, and FSH. A peripheral blood karyotype reported a 46XY with positive SRY gene.
2016-06-20	A brain MRI showed an Arnold Chiari malformation type 1.
2016-08-08	Moderate conductive bilateral hearing loss, corrected with hearing aids.
2018-10-08	Visit to explain the WES analysis and consent
2021-04-05	Visit to deliver the results

Supplementary figure 3. Alignment of Human, chimpanzee, gorilla, orangutan, rhesus monkey, house mouse and missense human variants in SOX9.

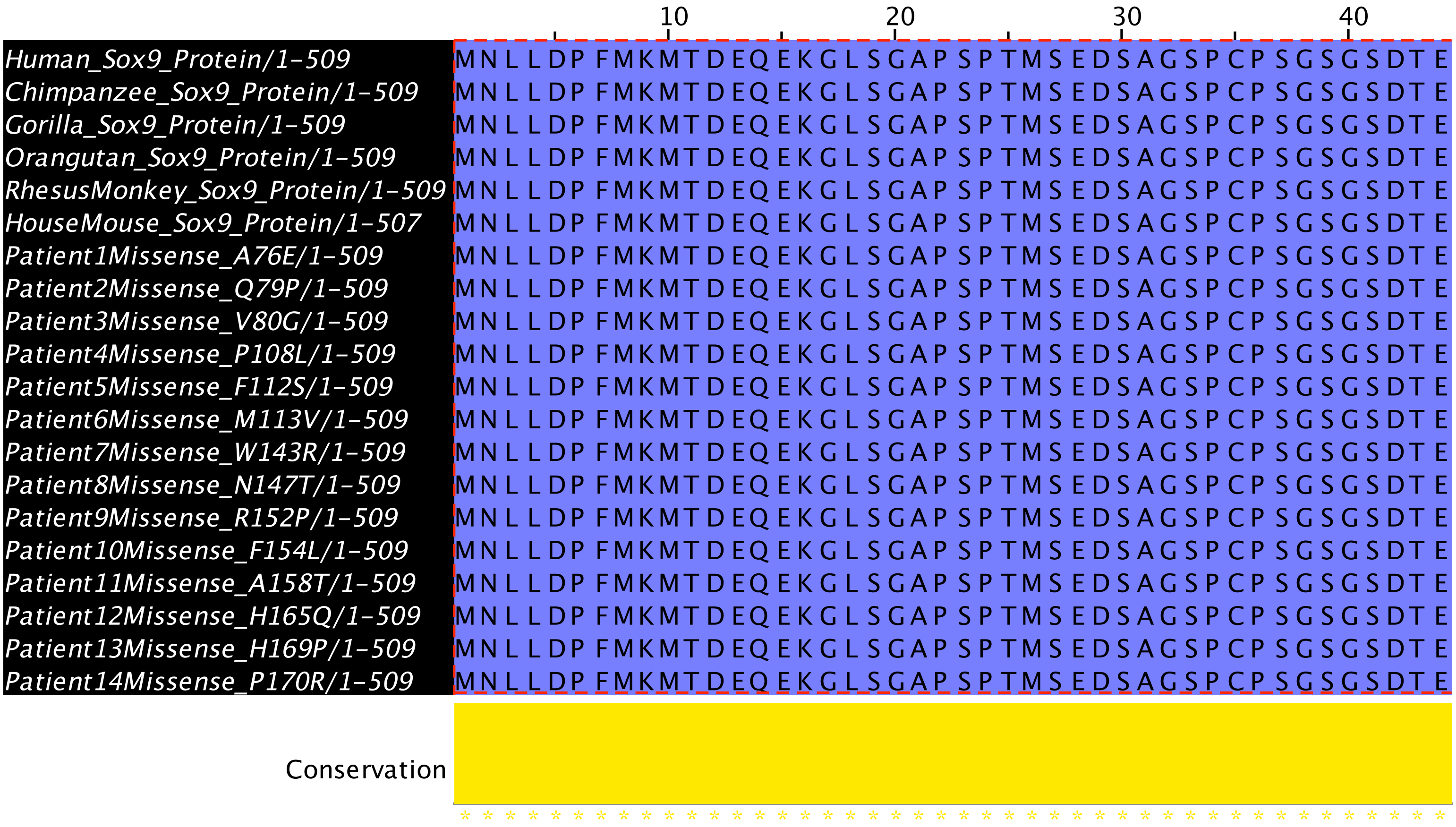

10
20
30
40
Human_Sox9_Protein/1-509 MNLLDPFMKMTDEQEKGLSGAPSPTMSEDSAGSPCPSGSGSDTE
Chimpanzee_Sox9_Protein/1-509 MNLLDPFMKMTDEQEKGLSGAPSPTMSEDSAGSPCPSGSGSDTE
Gorilla_Sox9_Protein/1-509 MNLLDPFMKMTDEQEKGLSGAPSPTMSEDSAGSPCPSGSGSDTE
Orangutan_Sox9_Protein/1-509 MNLLDPFMKMTDEQEKGLSGAPSPTMSEDSAGSPCPSGSGSDTE
RhesusMonkey_Sox9_Protein/1-509 MNLLDPFMKMTDEQEKGLSGAPSPTMSEDSAGSPCPSGSGSDTE
HouseMouse_Sox9_Protein/1-507 MNLLDPFMKMTDEQEKGLSGAPSPTMSEDSAGSPCPSGSGSDTE
Patient1Missense_A76E/1-509 MNLLDPFMKMTDEQEKGLSGAPSPTMSEDSAGSPCPSGSGSDTE
Patient2Missense_Q79P/1-509 MNLLDPFMKMTDEQEKGLSGAPSPTMSEDSAGSPCPSGSGSDTE
Patient3Missense_V80G/1-509 MNLLDPFMKMTDEQEKGLSGAPSPTMSEDSAGSPCPSGSGSDTE
Patient4Missense_P108L/1-509 MNLLDPFMKMTDEQEKGLSGAPSPTMSEDSAGSPCPSGSGSDTE
Patient5Missense_F112S/1-509 MNLLDPFMKMTDEQEKGLSGAPSPTMSEDSAGSPCPSGSGSDTE
Patient6Missense_M113V/1-509 MNLLDPFMKMTDEQEKGLSGAPSPTMSEDSAGSPCPSGSGSDTE
Patient7Missense_W143R/1-509 MNLLDPFMKMTDEQEKGLSGAPSPTMSEDSAGSPCPSGSGSDTE
Patient8Missense_N147T/1-509 MNLLDPFMKMTDEQEKGLSGAPSPTMSEDSAGSPCPSGSGSDTE
Patient9Missense_R152P/1-509 MNLLDPFMKMTDEQEKGLSGAPSPTMSEDSAGSPCPSGSGSDTE
Patient10Missense_F154L/1-509 MNLLDPFMKMTDEQEKGLSGAPSPTMSEDSAGSPCPSGSGSDTE
Patient11Missense_A158T/1-509 MNLLDPFMKMTDEQEKGLSGAPSPTMSEDSAGSPCPSGSGSDTE
Patient12Missense_H165Q/1-509 MNLLDPFMKMTDEQEKGLSGAPSPTMSEDSAGSPCPSGSGSDTE
Patient13Missense_H169P/1-509 MNLLDPFMKMTDEQEKGLSGAPSPTMSEDSAGSPCPSGSGSDTE
Patient14Missense_P170R/1-509 MNLLDPFMKMTDEQEKGLSGAPSPTMSEDSAGSPCPSGSGSDTE
Conservation

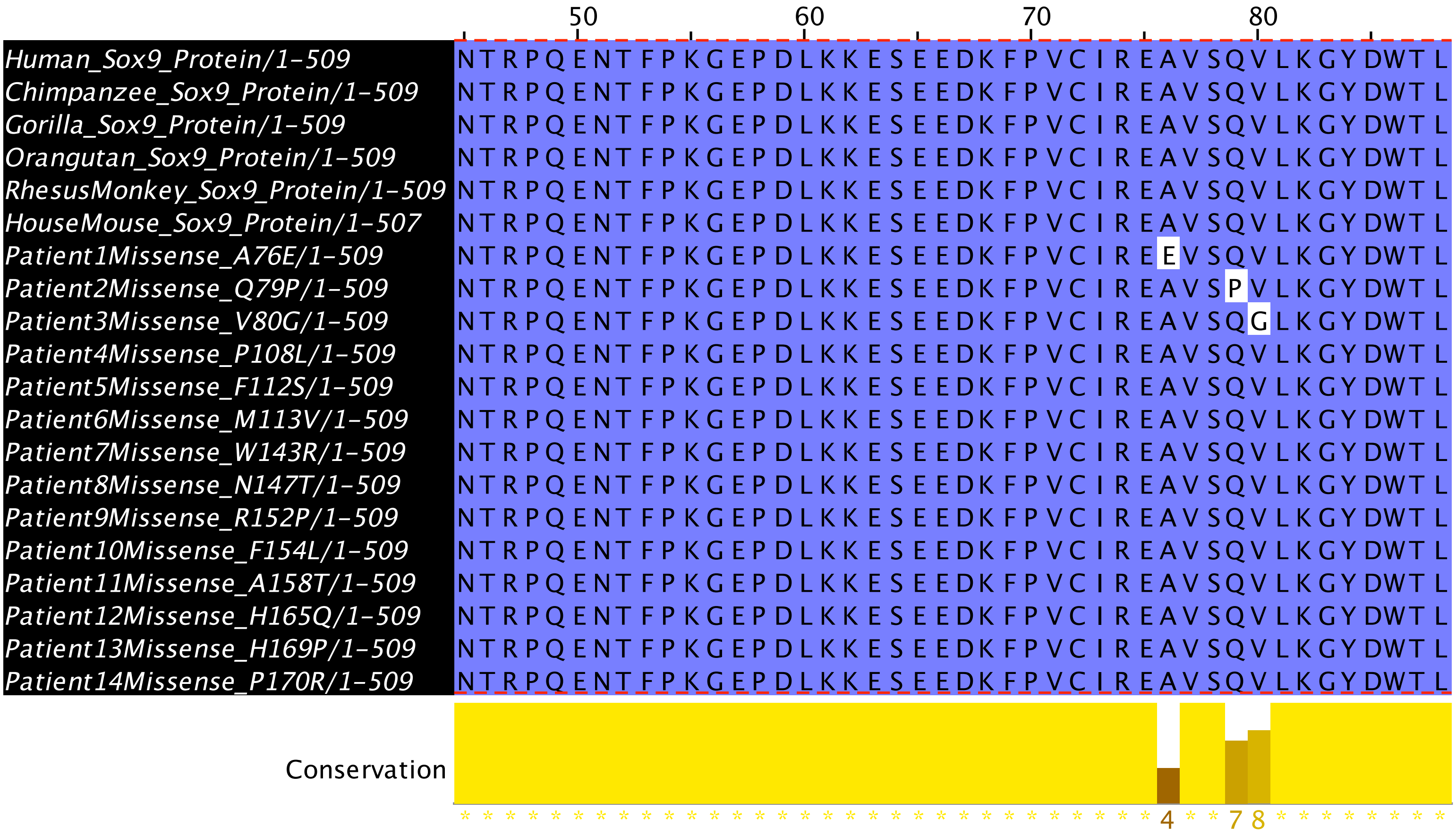
50
60
70
80
Human_Sox9_Protein/1-509 NTRPQENTFPKGEPDLKKESEEDKFPVCIREAVSQVLKGYDWTL
Chimpanzee_Sox9_Protein/1-509 NTRPQENTFPKGEPDLKKESEEDKFPVCIREAVSQVLKGYDWTL
Gorilla_Sox9_Protein/1-509 NTRPQENTFPKGEPDLKKESEEDKFPVCIREAVSQVLKGYDWTL
Orangutan_Sox9_Protein/1-509 NTRPQENTFPKGEPDLKKESEEDKFPVCIREAVSQVLKGYDWTL
RhesusMonkey_Sox9_Protein/1-509 NTRPQENTFPKGEPDLKKESEEDKFPVCIREAVSQVLKGYDWTL
HouseMouse_Sox9_Protein/1-507 NTRPQENTFPKGEPDLKKESEEDKFPVCIREAVSQVLKGYDWTL
Patient1Missense_A76E/1-509 NTRPQENTFPKGEPDLKKESEEDKFPVCIREEVSQVLKGYDWTL
Patient2Missense_Q79P/1-509 NTRPQENTFPKGEPDLKKESEEDKFPVCIREAVSPVLKGYDWTL
Patient3Missense_V80G/1-509 NTRPQENTFPKGEPDLKKESEEDKFPVCIREAVSQGLKGYDWTL
Patient4Missense_P108L/1-509 NTRPQENTFPKGEPDLKKESEEDKFPVCIREAVSQVLKGYDWTL
Patient5Missense_F112S/1-509 NTRPQENTFPKGEPDLKKESEEDKFPVCIREAVSQVLKGYDWTL
Patient6Missense_M113V/1-509 NTRPQENTFPKGEPDLKKESEEDKFPVCIREAVSQVLKGYDWTL
Patient7Missense_W143R/1-509 NTRPQENTFPKGEPDLKKESEEDKFPVCIREAVSQVLKGYDWTL
Patient8Missense_N147T/1-509 NTRPQENTFPKGEPDLKKESEEDKFPVCIREAVSQVLKGYDWTL
Patient9Missense_R152P/1-509 NTRPQENTFPKGEPDLKKESEEDKFPVCIREAVSQVLKGYDWTL
Patient10Missense_F154L/1-509 NTRPQENTFPKGEPDLKKESEEDKFPVCIREAVSQVLKGYDWTL
Patient11Missense_A158T/1-509 NTRPQENTFPKGEPDLKKESEEDKFPVCIREAVSQVLKGYDWTL
Patient12Missense_H165Q/1-509 NTRPQENTFPKGEPDLKKESEEDKFPVCIREAVSQVLKGYDWTL
Patient13Missense_H169P/1-509 NTRPQENTFPKGEPDLKKESEEDKFPVCIREAVSQVLKGYDWTL
Patient14Missense_P170R/1-509 NTRPQENTFPKGEPDLKKESEEDKFPVCIREAVSQVLKGYDWTL
Conservation
******************************4**78********

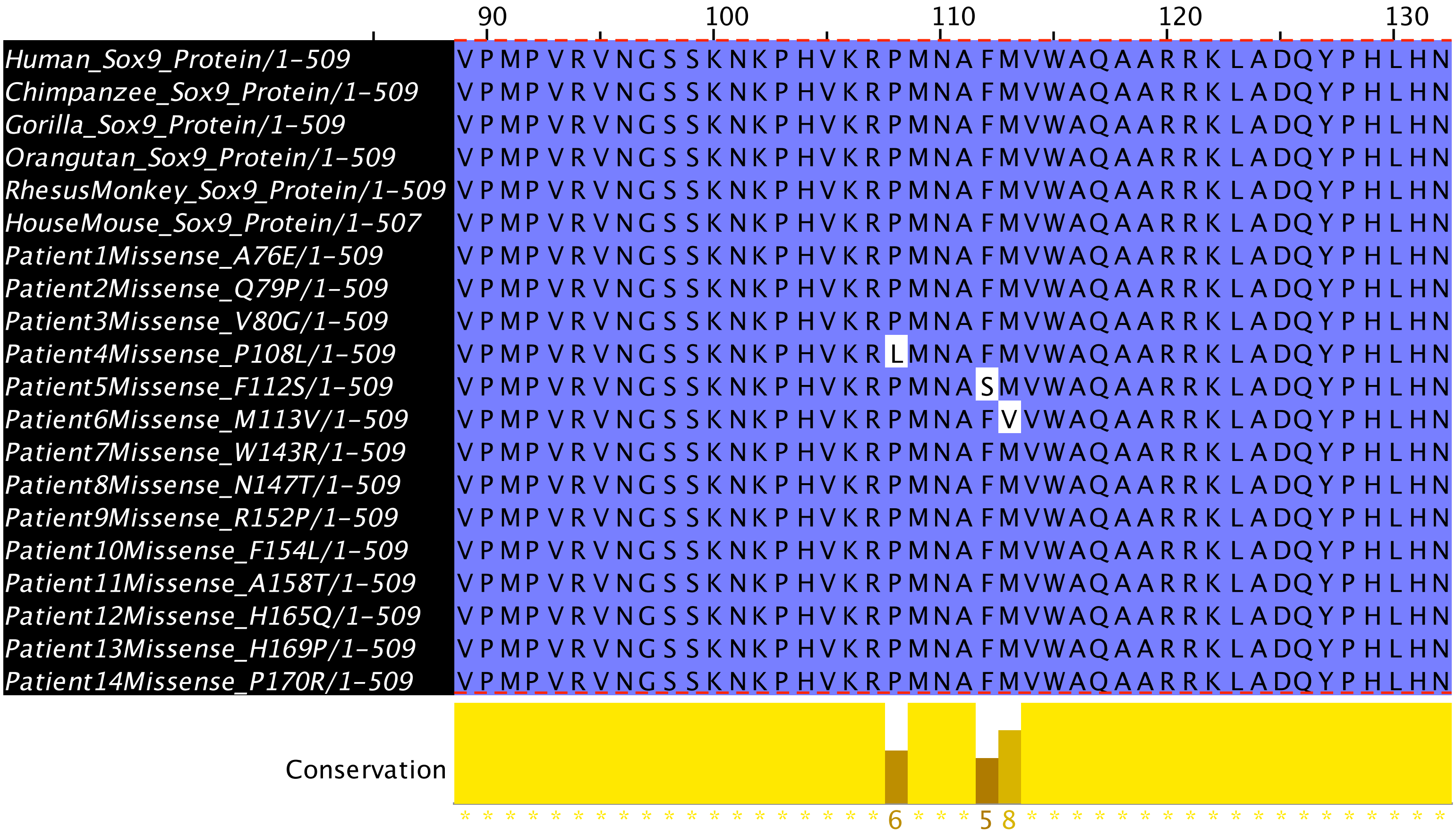

90 100 110 120 130
Human_Sox9_Protein/1-509
Chimpanzee_Sox9_Protein/1-509
Gorilla_Sox9_Protein/1-509
Orangutan_Sox9_Protein/1-509
RhesusMonkey_Sox9_Protein/1-509
HouseMouse_Sox9_Protein/1-507
Patient1Missense_A76E/1-509
Patient2Missense_Q79P/1-509
Patient3Missense_V80G/1-509
Patient4Missense_P108L/1-509
Patient5Missense_F112S/1-509
Patient6Missense_M113V/1-509
Patient7Missense_W143R/1-509
Patient8Missense_N147T/1-509
Patient9Missense_R152P/1-509
Patient10Missense_F154L/1-509
Patient11Missense_A158T/1-509
Patient12Missense_H165Q/1-509
Patient13Missense_H169P/1-509
Patient14Missense_P170R/1-509
VPMPVRVNGSSKNKPHVKRPMNAFMVWAQAARRKLADQYPHLHN
Conservation

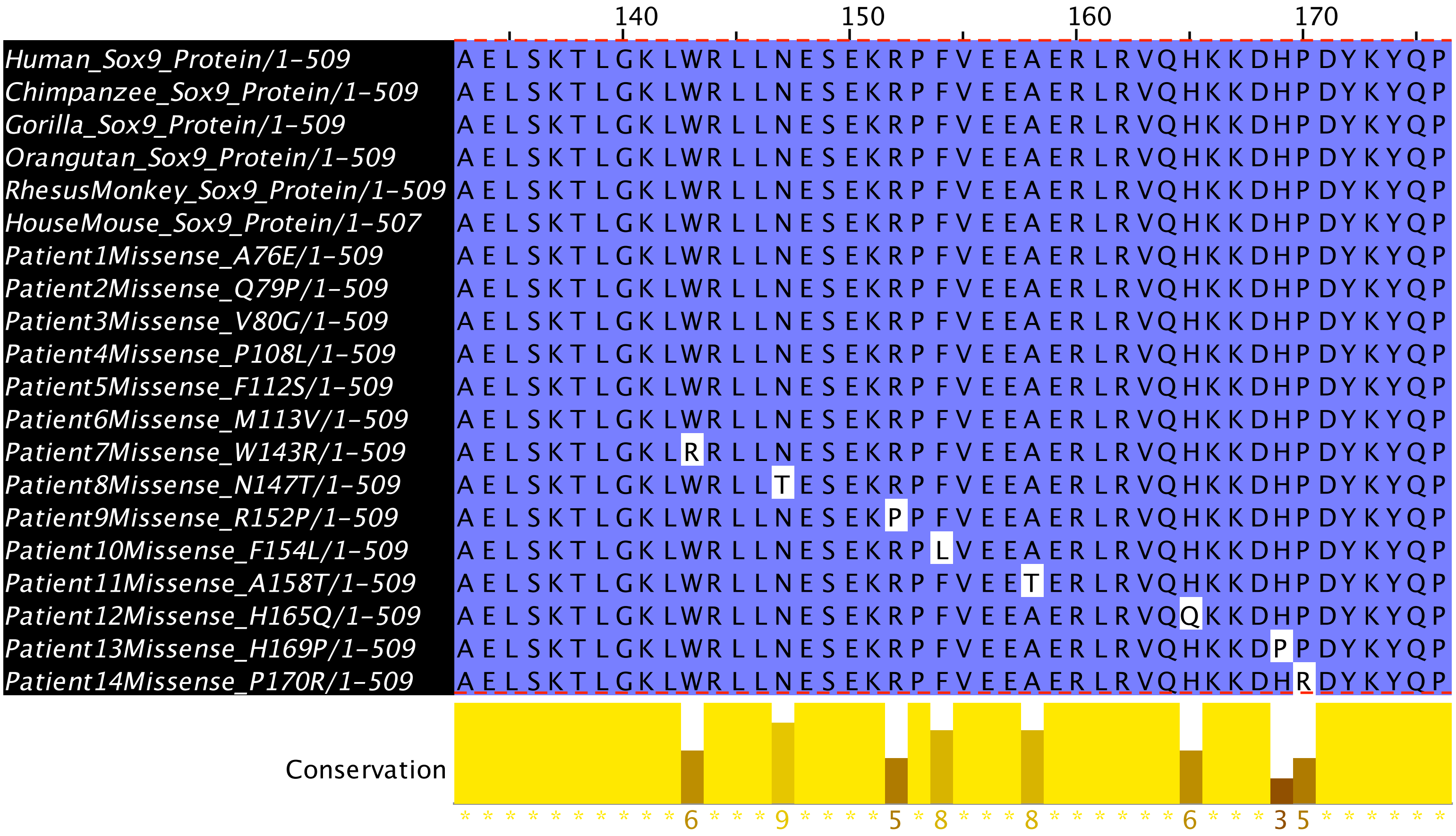
140
150
160
170
Human_Sox9_Protein/1-509
Chimpanzee_Sox9_Protein/1-509
Gorilla_Sox9_Protein/1-509
Orangutan_Sox9_Protein/1-509
RhesusMonkey_Sox9_Protein/1-509
HouseMouse_Sox9_Protein/1-507
Patient1Missense_A76E/1-509
Patient2Missense_Q79P/1-509
Patient3Missense_V80G/1-509
Patient4Missense_P108L/1-509
Patient5Missense_F112S/1-509
Patient6Missense_M113V/1-509
Patient7Missense_W143R/1-509
Patient8Missense_N147T/1-509
Patient9Missense_R152P/1-509
Patient10Missense_F154L/1-509
Patient11Missense_A158T/1-509
Patient12Missense_H165Q/1-509
Patient13Missense_H169P/1-509
Patient14Missense_P170R/1-509
AELSKTLGKLWRLLNESEKRPFVEEAERLRVQHKKDHPDYKYQP
Conservation
* * * * * * * * * * 6 * * * 9 * * * * 5 * 8 * * * 8 * * * * * * 6 * * * 3 5 * * * * * *

Sequence	180 190 200 210
Human_Sox9_Protein/1-509	RRRKSVKNGQAEAEEATEQTHISPNAIFKALQADSPHSSSGMSE
Chimpanzee_Sox9_Protein/1-509	RRRKSVKNGQAEAEEATEQTHISPNAIFKALQADSPHSSSGMSE
Gorilla_Sox9_Protein/1-509	RRRKSVKNGQAEAEEATEQTHISPNAIFKALQADSPHSSSGMSE
Orangutan_Sox9_Protein/1-509	RRRKSVKNGQAEAEEATEQTHISPNAIFKALQADSPHSSSGMSE
RhesusMonkey_Sox9_Protein/1-509	RRRKSVKNGQAEAEEATEQTHISPNAIFKALQADSPHSSSGMSE
HouseMouse_Sox9_Protein/1-507	RRRKSVKNGQAEAEEATEQTHISPNAIFKALQADSPHSSSGMSE
Patient1Missense_A76E/1-509	RRRKSVKNGQAEAEEATEQTHISPNAIFKALQADSPHSSSGMSE
Patient2Missense_Q79P/1-509	RRRKSVKNGQAEAEEATEQTHISPNAIFKALQADSPHSSSGMSE
Patient3Missense_V80G/1-509	RRRKSVKNGQAEAEEATEQTHISPNAIFKALQADSPHSSSGMSE
Patient4Missense_P108L/1-509	RRRKSVKNGQAEAEEATEQTHISPNAIFKALQADSPHSSSGMSE
Patient5Missense_F112S/1-509	RRRKSVKNGQAEAEEATEQTHISPNAIFKALQADSPHSSSGMSE
Patient6Missense_M113V/1-509	RRRKSVKNGQAEAEEATEQTHISPNAIFKALQADSPHSSSGMSE
Patient7Missense_W143R/1-509	RRRKSVKNGQAEAEEATEQTHISPNAIFKALQADSPHSSSGMSE
Patient8Missense_N147T/1-509	RRRKSVKNGQAEAEEATEQTHISPNAIFKALQADSPHSSSGMSE
Patient9Missense_R152P/1-509	RRRKSVKNGQAEAEEATEQTHISPNAIFKALQADSPHSSSGMSE
Patient10Missense_F154L/1-509	RRRKSVKNGQAEAEEATEQTHISPNAIFKALQADSPHSSSGMSE
Patient11Missense_A158T/1-509	RRRKSVKNGQAEAEEATEQTHISPNAIFKALQADSPHSSSGMSE
Patient12Missense_H165Q/1-509	RRRKSVKNGQAEAEEATEQTHISPNAIFKALQADSPHSSSGMSE
Patient13Missense_H169P/1-509	RRRKSVKNGQAEAEEATEQTHISPNAIFKALQADSPHSSSGMSE
Patient14Missense_P170R/1-509	RRRKSVKNGQAEAEEATEQTHISPNAIFKALQADSPHSSSGMSE
Conservation	**

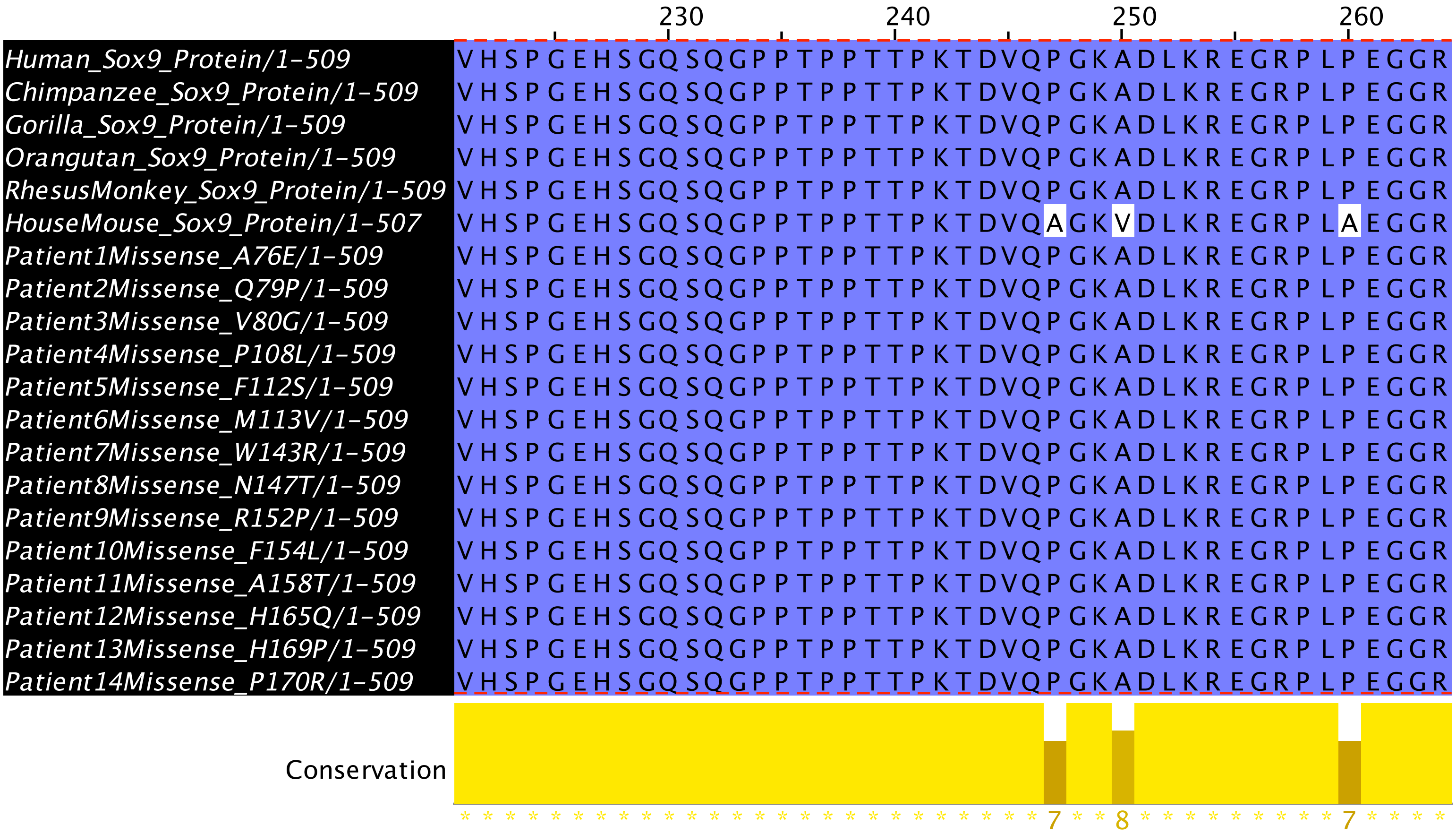

230
240
250
260
Human_Sox9_Protein/1-509 VHSPGEHSGQSQGPPTPPTTPKTDVQPGKADLKREGRPLPEGGR
Chimpanzee_Sox9_Protein/1-509 VHSPGEHSGQSQGPPTPPTTPKTDVQPGKADLKREGRPLPEGGR
Gorilla_Sox9_Protein/1-509 VHSPGEHSGQSQGPPTPPTTPKTDVQPGKADLKREGRPLPEGGR
Orangutan_Sox9_Protein/1-509 VHSPGEHSGQSQGPPTPPTTPKTDVQPGKADLKREGRPLPEGGR
RhesusMonkey_Sox9_Protein/1-509 VHSPGEHSGQSQGPPTPPTTPKTDVQPGKADLKREGRPLPEGGR
HouseMouse_Sox9_Protein/1-507 VHSPGEHSGQSQGPPTPPTTPKTDVQAGKVDLKREGRPLAEGGR
Patient1Missense_A76E/1-509 VHSPGEHSGQSQGPPTPPTTPKTDVQPGKADLKREGRPLPEGGR
Patient2Missense_Q79P/1-509 VHSPGEHSGQSQGPPTPPTTPKTDVQPGKADLKREGRPLPEGGR
Patient3Missense_V80G/1-509 VHSPGEHSGQSQGPPTPPTTPKTDVQPGKADLKREGRPLPEGGR
Patient4Missense_P108L/1-509 VHSPGEHSGQSQGPPTPPTTPKTDVQPGKADLKREGRPLPEGGR
Patient5Missense_F112S/1-509 VHSPGEHSGQSQGPPTPPTTPKTDVQPGKADLKREGRPLPEGGR
Patient6Missense_M113V/1-509 VHSPGEHSGQSQGPPTPPTTPKTDVQPGKADLKREGRPLPEGGR
Patient7Missense_W143R/1-509 VHSPGEHSGQSQGPPTPPTTPKTDVQPGKADLKREGRPLPEGGR
Patient8Missense_N147T/1-509 VHSPGEHSGQSQGPPTPPTTPKTDVQPGKADLKREGRPLPEGGR
Patient9Missense_R152P/1-509 VHSPGEHSGQSQGPPTPPTTPKTDVQPGKADLKREGRPLPEGGR
Patient10Missense_F154L/1-509 VHSPGEHSGQSQGPPTPPTTPKTDVQPGKADLKREGRPLPEGGR
Patient11Missense_A158T/1-509 VHSPGEHSGQSQGPPTPPTTPKTDVQPGKADLKREGRPLPEGGR
Patient12Missense_H165Q/1-509 VHSPGEHSGQSQGPPTPPTTPKTDVQPGKADLKREGRPLPEGGR
Patient13Missense_H169P/1-509 VHSPGEHSGQSQGPPTPPTTPKTDVQPGKADLKREGRPLPEGGR
Patient14Missense_P170R/1-509 VHSPGEHSGQSQGPPTPPTTPKTDVQPGKADLKREGRPLPEGGR
Conservation
**************************7**8**********7****

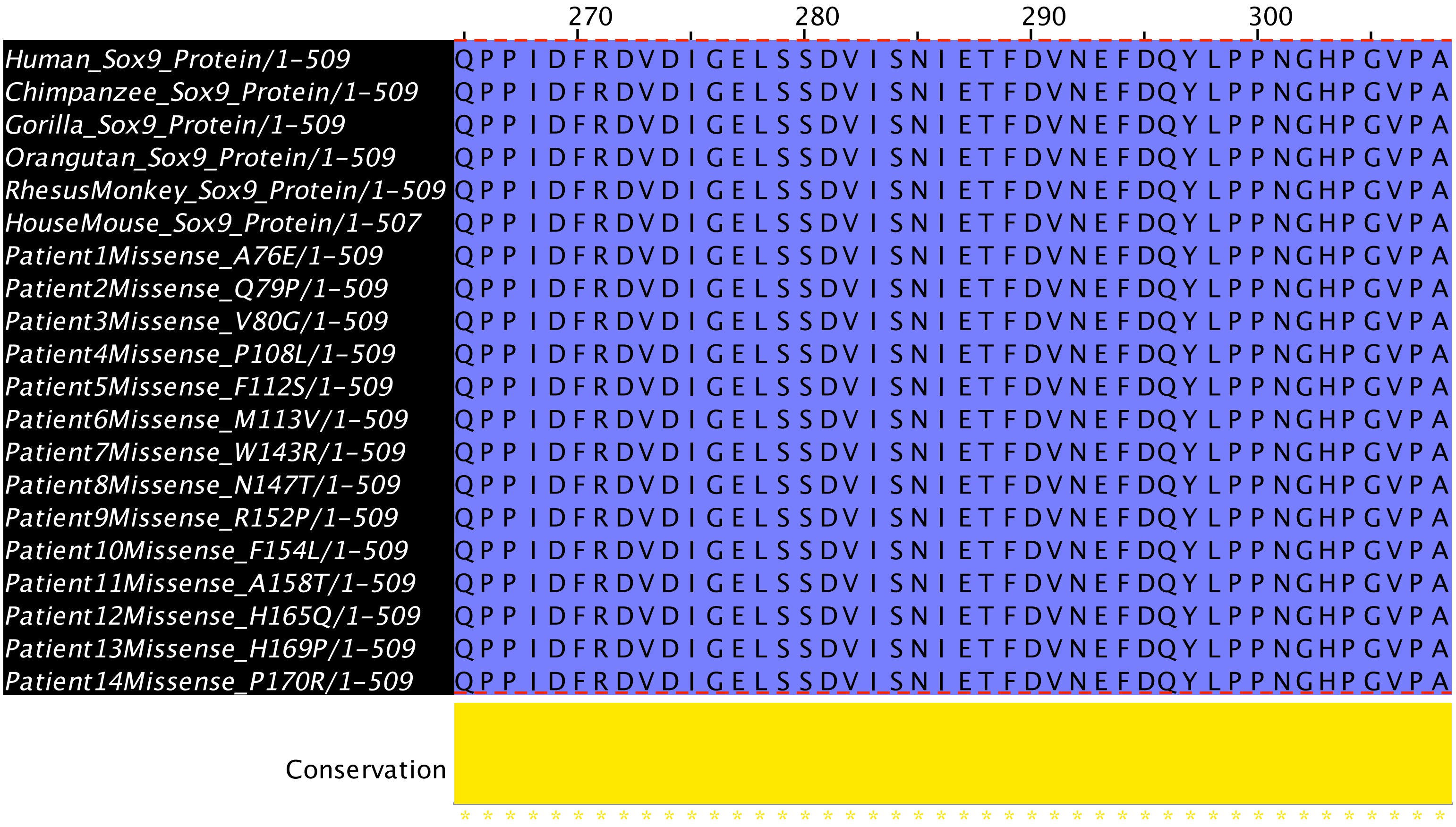
270
280
290
300
Human_Sox9_Protein/1-509 QPPIDFRDVDIGELSSDVISNIETFDVNEFDQYLPPNGHPGVPA
Chimpanzee_Sox9_Protein/1-509 QPPIDFRDVDIGELSSDVISNIETFDVNEFDQYLPPNGHPGVPA
Gorilla_Sox9_Protein/1-509 QPPIDFRDVDIGELSSDVISNIETFDVNEFDQYLPPNGHPGVPA
Orangutan_Sox9_Protein/1-509 QPPIDFRDVDIGELSSDVISNIETFDVNEFDQYLPPNGHPGVPA
RhesusMonkey_Sox9_Protein/1-509 QPPIDFRDVDIGELSSDVISNIETFDVNEFDQYLPPNGHPGVPA
HouseMouse_Sox9_Protein/1-507 QPPIDFRDVDIGELSSDVISNIETFDVNEFDQYLPPNGHPGVPA
Patient1Missense_A76E/1-509 QPPIDFRDVDIGELSSDVISNIETFDVNEFDQYLPPNGHPGVPA
Patient2Missense_Q79P/1-509 QPPIDFRDVDIGELSSDVISNIETFDVNEFDQYLPPNGHPGVPA
Patient3Missense_V80G/1-509 QPPIDFRDVDIGELSSDVISNIETFDVNEFDQYLPPNGHPGVPA
Patient4Missense_P108L/1-509 QPPIDFRDVDIGELSSDVISNIETFDVNEFDQYLPPNGHPGVPA
Patient5Missense_F112S/1-509 QPPIDFRDVDIGELSSDVISNIETFDVNEFDQYLPPNGHPGVPA
Patient6Missense_M113V/1-509 QPPIDFRDVDIGELSSDVISNIETFDVNEFDQYLPPNGHPGVPA
Patient7Missense_W143R/1-509 QPPIDFRDVDIGELSSDVISNIETFDVNEFDQYLPPNGHPGVPA
Patient8Missense_N147T/1-509 QPPIDFRDVDIGELSSDVISNIETFDVNEFDQYLPPNGHPGVPA
Patient9Missense_R152P/1-509 QPPIDFRDVDIGELSSDVISNIETFDVNEFDQYLPPNGHPGVPA
Patient10Missense_F154L/1-509 QPPIDFRDVDIGELSSDVISNIETFDVNEFDQYLPPNGHPGVPA
Patient11Missense_A158T/1-509 QPPIDFRDVDIGELSSDVISNIETFDVNEFDQYLPPNGHPGVPA
Patient12Missense_H165Q/1-509 QPPIDFRDVDIGELSSDVISNIETFDVNEFDQYLPPNGHPGVPA
Patient13Missense_H169P/1-509 QPPIDFRDVDIGELSSDVISNIETFDVNEFDQYLPPNGHPGVPA
Patient14Missense_P170R/1-509 QPPIDFRDVDIGELSSDVISNIETFDVNEFDQYLPPNGHPGVPA
Conservation

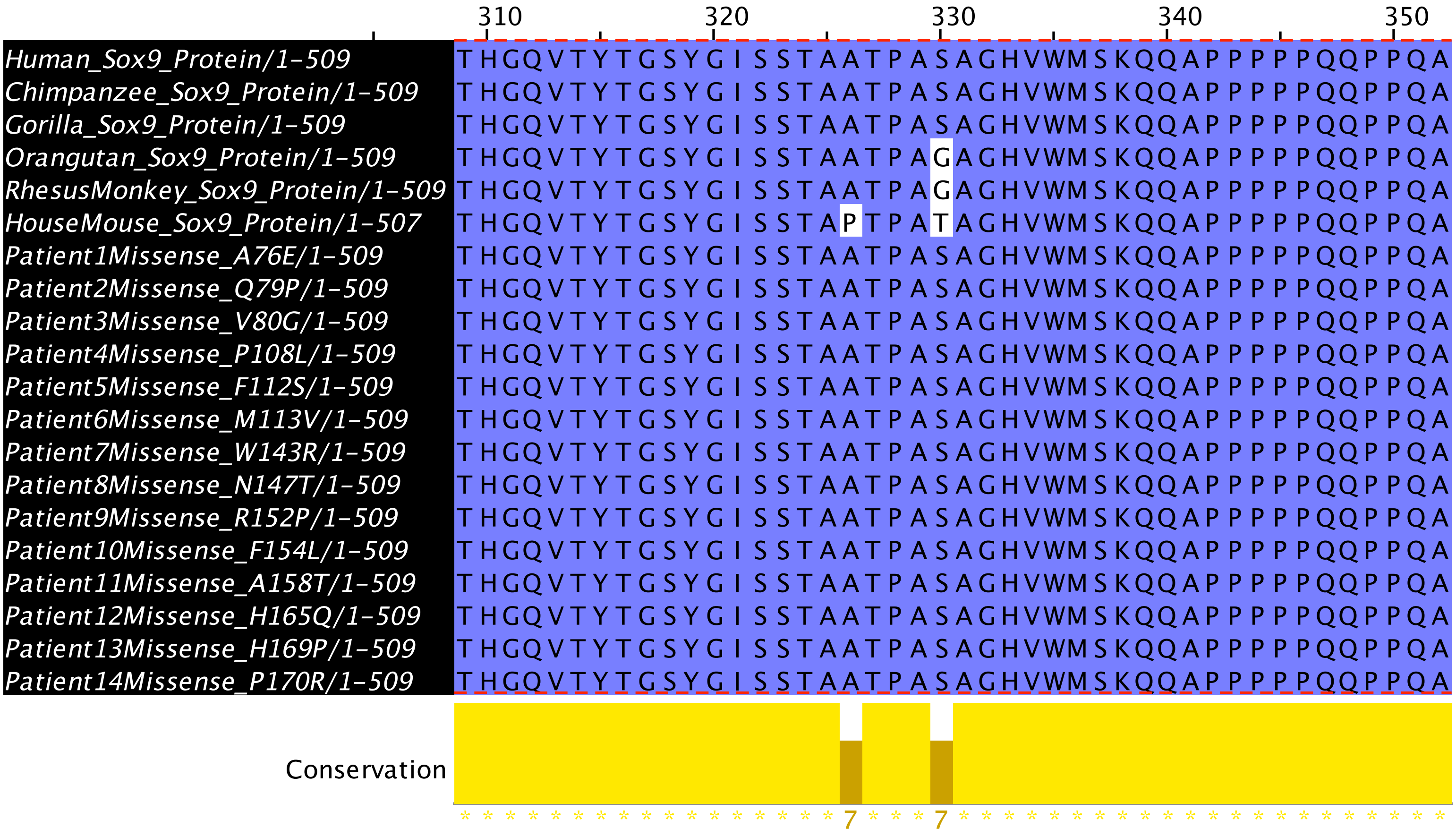

310
320
330
340
350
Human_Sox9_Protein/1-509
Chimpanzee_Sox9_Protein/1-509
Gorilla_Sox9_Protein/1-509
Orangutan_Sox9_Protein/1-509
RhesusMonkey_Sox9_Protein/1-509
HouseMouse_Sox9_Protein/1-507
Patient1Missense_A76E/1-509
Patient2Missense_Q79P/1-509
Patient3Missense_V80G/1-509
Patient4Missense_P108L/1-509
Patient5Missense_F112S/1-509
Patient6Missense_M113V/1-509
Patient7Missense_W143R/1-509
Patient8Missense_N147T/1-509
Patient9Missense_R152P/1-509
Patient10Missense_F154L/1-509
Patient11Missense_A158T/1-509
Patient12Missense_H165Q/1-509
Patient13Missense_H169P/1-509
Patient14Missense_P170R/1-509
THGQVTYTGSYGISSTAATPASAGHVWMSKQQAPPPPPQQPPQA
THGQVTYTGSYGISSTAATPAGAGHVWMSKQQAPPPPPQQPPQA
THGQVTYTGSYGISSTAPTPATAGHVWMSKQQAPPPPPQQPPQA
Conservation
* * * * * * * * * * * * * * * * * 7 * * * 7 *

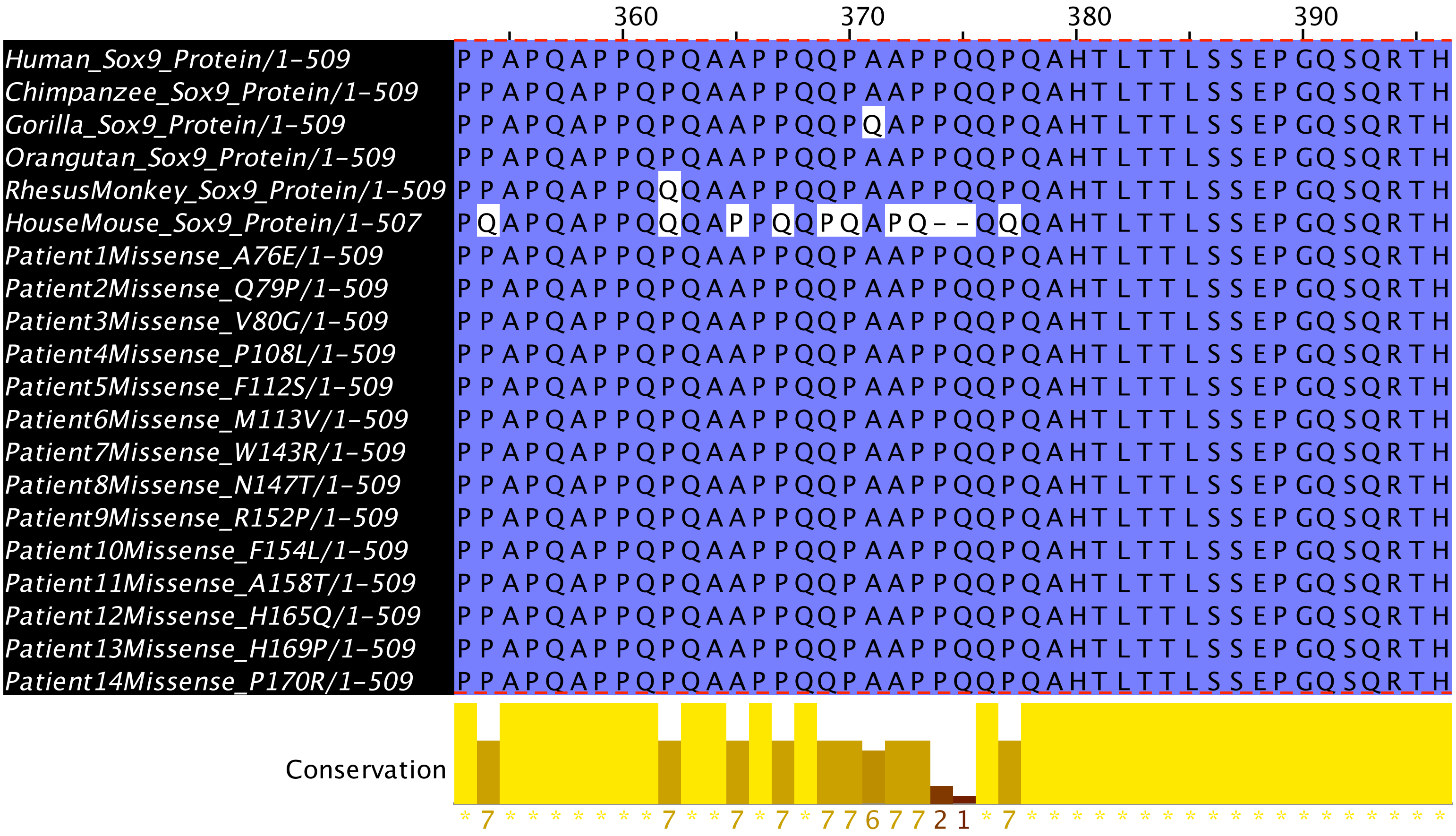
360
370
380
390
Human_Sox9_Protein/1-509
Chimpanzee_Sox9_Protein/1-509
Gorilla_Sox9_Protein/1-509
Orangutan_Sox9_Protein/1-509
RhesusMonkey_Sox9_Protein/1-509
HouseMouse_Sox9_Protein/1-507
Patient1Missense_A76E/1-509
Patient2Missense_Q79P/1-509
Patient3Missense_V80G/1-509
Patient4Missense_P108L/1-509
Patient5Missense_F112S/1-509
Patient6Missense_M113V/1-509
Patient7Missense_W143R/1-509
Patient8Missense_N147T/1-509
Patient9Missense_R152P/1-509
Patient10Missense_F154L/1-509
Patient11Missense_A158T/1-509
Patient12Missense_H165Q/1-509
Patient13Missense_H169P/1-509
Patient14Missense_P170R/1-509
Conservation

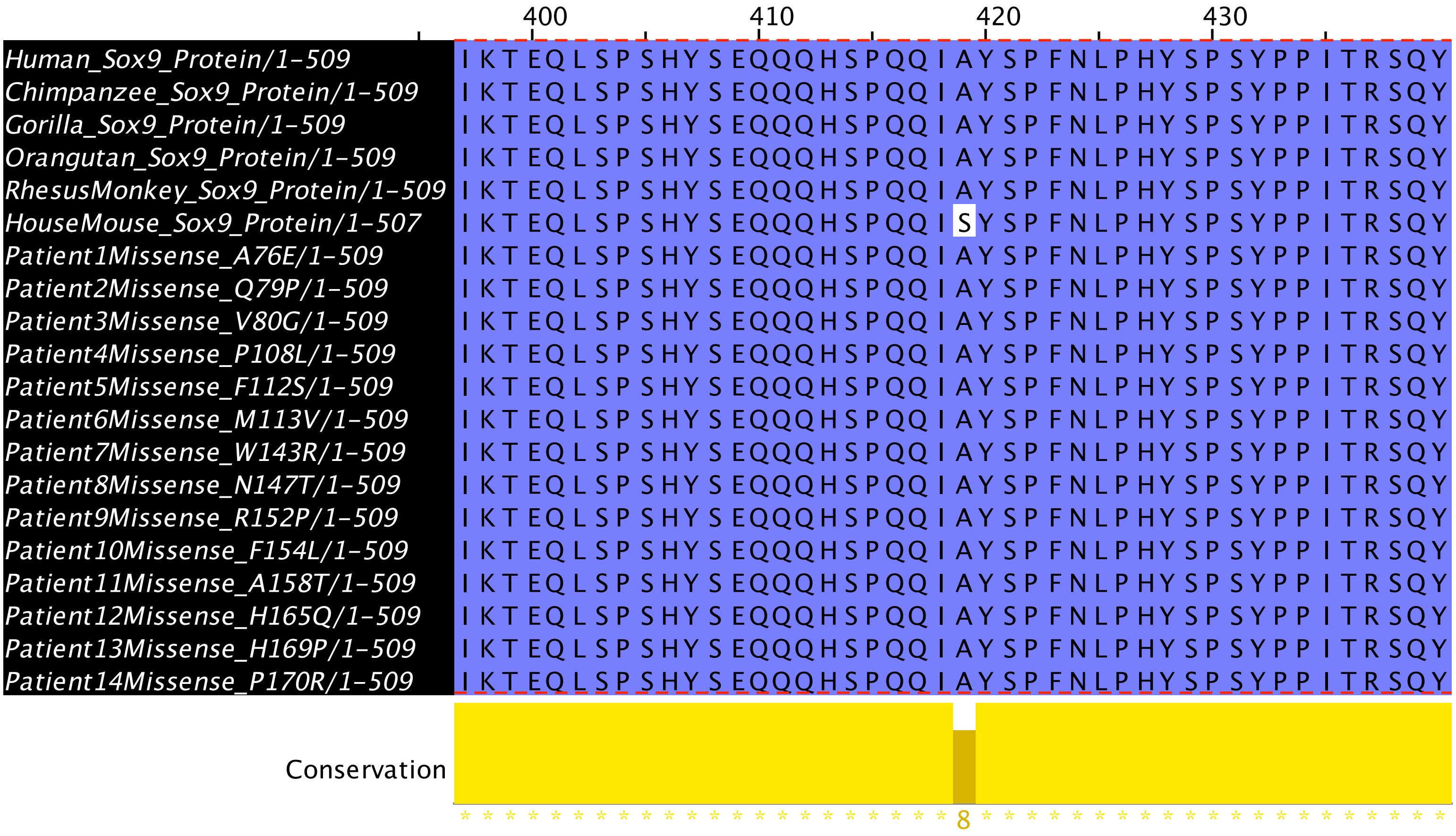

400
410
420
430
Human_Sox9_Protein/1-509
Chimpanzee_Sox9_Protein/1-509
Gorilla_Sox9_Protein/1-509
Orangutan_Sox9_Protein/1-509
RhesusMonkey_Sox9_Protein/1-509
HouseMouse_Sox9_Protein/1-507
Patient1Missense_A76E/1-509
Patient2Missense_Q79P/1-509
Patient3Missense_V80G/1-509
Patient4Missense_P108L/1-509
Patient5Missense_F112S/1-509
Patient6Missense_M113V/1-509
Patient7Missense_W143R/1-509
Patient8Missense_N147T/1-509
Patient9Missense_R152P/1-509
Patient10Missense_F154L/1-509
Patient11Missense_A158T/1-509
Patient12Missense_H165Q/1-509
Patient13Missense_H169P/1-509
Patient14Missense_P170R/1-509
IKTEQLSPSHYSEQQQHSPQQIAYSPFNLPHYSPSYPPITRSQY
IKTEQLSPSHYSEQQQHSPQQISYSPFNLPHYSPSYPPITRSQY
Conservation

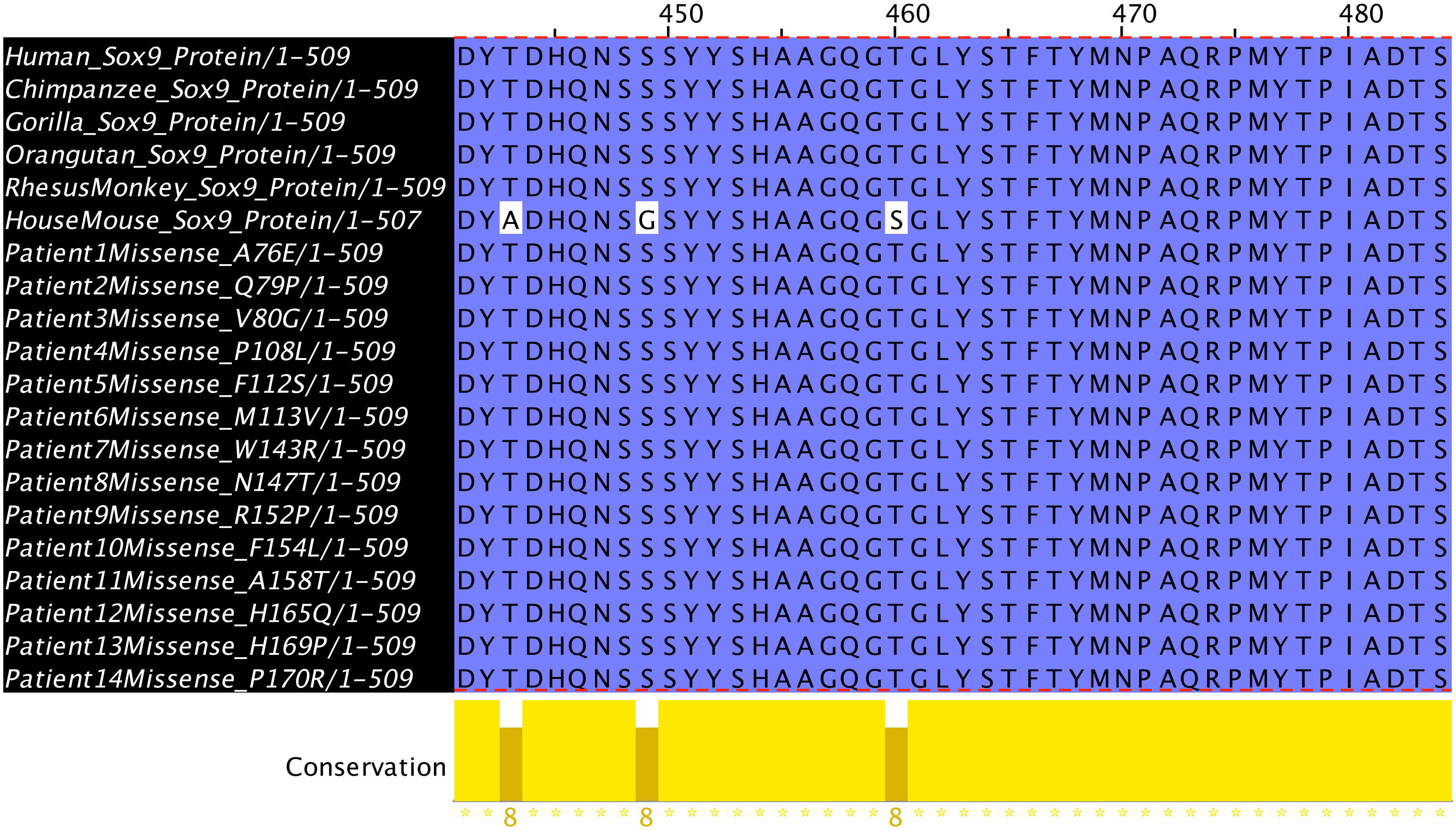
450
460
470
480
Human_Sox9_Protein/1-509 DYTDHQNSSSYYSHAAGQGTGLYSTFTYMNPAQRPMYTPIADTS
Chimpanzee_Sox9_Protein/1-509 DYTDHQNSSSYYSHAAGQGTGLYSTFTYMNPAQRPMYTPIADTS
Gorilla_Sox9_Protein/1-509 DYTDHQNSSSYYSHAAGQGTGLYSTFTYMNPAQRPMYTPIADTS
Orangutan_Sox9_Protein/1-509 DYTDHQNSSSYYSHAAGQGTGLYSTFTYMNPAQRPMYTPIADTS
RhesusMonkey_Sox9_Protein/1-509 DYTDHQNSSSYYSHAAGQGTGLYSTFTYMNPAQRPMYTPIADTS
HouseMouse_Sox9_Protein/1-507 DYADHQNSGSYYSHAAGQGSGLYSTFTYMNPAQRPMYTPIADTS
Patient1Missense_A76E/1-509 DYTDHQNSSSYYSHAAGQGTGLYSTFTYMNPAQRPMYTPIADTS
Patient2Missense_Q79P/1-509 DYTDHQNSSSYYSHAAGQGTGLYSTFTYMNPAQRPMYTPIADTS
Patient3Missense_V80G/1-509 DYTDHQNSSSYYSHAAGQGTGLYSTFTYMNPAQRPMYTPIADTS
Patient4Missense_P108L/1-509 DYTDHQNSSSYYSHAAGQGTGLYSTFTYMNPAQRPMYTPIADTS
Patient5Missense_F112S/1-509 DYTDHQNSSSYYSHAAGQGTGLYSTFTYMNPAQRPMYTPIADTS
Patient6Missense_M113V/1-509 DYTDHQNSSSYYSHAAGQGTGLYSTFTYMNPAQRPMYTPIADTS
Patient7Missense_W143R/1-509 DYTDHQNSSSYYSHAAGQGTGLYSTFTYMNPAQRPMYTPIADTS
Patient8Missense_N147T/1-509 DYTDHQNSSSYYSHAAGQGTGLYSTFTYMNPAQRPMYTPIADTS
Patient9Missense_R152P/1-509 DYTDHQNSSSYYSHAAGQGTGLYSTFTYMNPAQRPMYTPIADTS
Patient10Missense_F154L/1-509 DYTDHQNSSSYYSHAAGQGTGLYSTFTYMNPAQRPMYTPIADTS
Patient11Missense_A158T/1-509 DYTDHQNSSSYYSHAAGQGTGLYSTFTYMNPAQRPMYTPIADTS
Patient12Missense_H165Q/1-509 DYTDHQNSSSYYSHAAGQGTGLYSTFTYMNPAQRPMYTPIADTS
Patient13Missense_H169P/1-509 DYTDHQNSSSYYSHAAGQGTGLYSTFTYMNPAQRPMYTPIADTS
Patient14Missense_P170R/1-509 DYTDHQNSSSYYSHAAGQGTGLYSTFTYMNPAQRPMYTPIADTS
Conservation

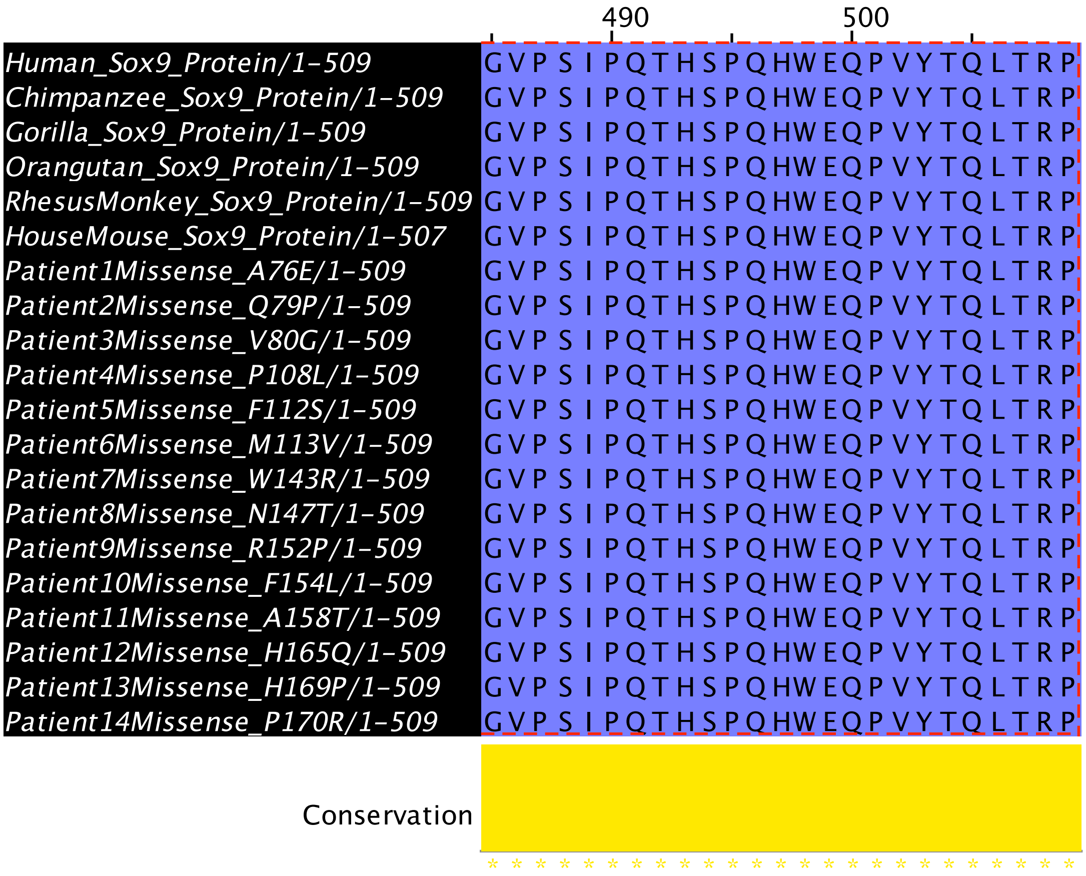
490
500
Human_Sox9_Protein/1-509 GVPSIPQTHSPQHWEQPVYTQLTRP
Chimpanzee_Sox9_Protein/1-509 GVPSIPQTHSPQHWEQPVYTQLTRP
Gorilla_Sox9_Protein/1-509 GVPSIPQTHSPQHWEQPVYTQLTRP
Orangutan_Sox9_Protein/1-509 GVPSIPQTHSPQHWEQPVYTQLTRP
RhesusMonkey_Sox9_Protein/1-509 GVPSIPQTHSPQHWEQPVYTQLTRP
HouseMouse_Sox9_Protein/1-507 GVPSIPQTHSPQHWEQPVYTQLTRP
Patient1Missense_A76E/1-509 GVPSIPQTHSPQHWEQPVYTQLTRP
Patient2Missense_Q79P/1-509 GVPSIPQTHSPQHWEQPVYTQLTRP
Patient3Missense_V80G/1-509 GVPSIPQTHSPQHWEQPVYTQLTRP
Patient4Missense_P108L/1-509 GVPSIPQTHSPQHWEQPVYTQLTRP
Patient5Missense_F112S/1-509 GVPSIPQTHSPQHWEQPVYTQLTRP
Patient6Missense_M113V/1-509 GVPSIPQTHSPQHWEQPVYTQLTRP
Patient7Missense_W143R/1-509 GVPSIPQTHSPQHWEQPVYTQLTRP
Patient8Missense_N147T/1-509 GVPSIPQTHSPQHWEQPVYTQLTRP
Patient9Missense_R152P/1-509 GVPSIPQTHSPQHWEQPVYTQLTRP
Patient10Missense_F154L/1-509 GVPSIPQTHSPQHWEQPVYTQLTRP
Patient11Missense_A158T/1-509 GVPSIPQTHSPQHWEQPVYTQLTRP
Patient12Missense_H165Q/1-509 GVPSIPQTHSPQHWEQPVYTQLTRP
Patient13Missense_H169P/1-509 GVPSIPQTHSPQHWEQPVYTQLTRP
Patient14Missense_P170R/1-509 GVPSIPQTHSPQHWEQPVYTQLTRP
Conservation
****

Supplementary figure 4. A) Percentage of similarity between human and human, chimpanzee, gorilla, orangutan, rhesus monkey and house mouse. **B)** Phylogenetic tree using SOX9 aminoacid sequences of human, chimpanzee, gorilla, orangutan, rhesus monkey and house mouse. Human and chimpanzee group together and the outcast is house mouse.

A)

	**Similarity with Human sequence (%)**
Human	100
Chimpanzee	100
Gorilla	99.8
Orangutan	99.8
RhesusMonkey	99.61
HouseMouse	96.45

B)

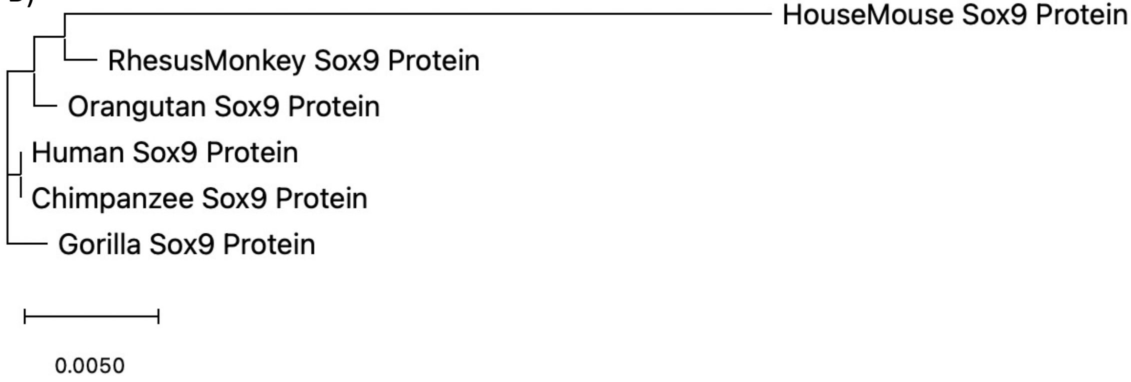

Supplementary table 1. Articles of patients with Campomelic dysplasia and the causal variant.

Variant	Title	Journal	DOI
**E28X**	Campomelic Dysplasia: echographic suspicion in the first trimester of pregnancy and final diagnosis of two cases	Fetal diagnosis and therapy	10.1159/000176299
**59X (C155bp)**	The phenotype of survivors of campomelic dysplasia	Journal of medical genetics	10.1136/jmg.39.8.597
**W86X**	Mutational analysis of the SOX9 gene in campomelic dysplasia and autosomal sex reversal: lack of genotype/phenotype correlations	Human Molecular genetics	https://doi.org/10.1093/hmg/6.1.91
**261-262insG**	Patient reports: Two novel frameshift mutations in the SOX9 gene in two patients with campomelic dyplasia who showed long term survival	Journal of pediactric endocrinology and metabolism	10.1515/jpem.2010.187
**Q117X**	Mutational analysis of the SOX9 gene in campomelic dysplasia and autosomal sex reversal: lack of genotype/phenotype correlations	Human Molecular genetics	https://doi.org/10.1093/hmg/6.1.91
**Y319X**	Dominant negative SOX9 mutations in campomelic dysplasia	Human Mutations	10.1002/humu.23888
**Q375X**	Mutational analysis of the SOX9 gene in campomelic dysplasia and autosomal sex reversal: lack of genotype/phenotype correlations	Human Molecular genetics	https://doi.org/10.1093/hmg/6.1.91
**Q391X**	Dominant negative SOX9 mutations in campomelic dysplasia	Human Mutations	10.1002/humu.23888
**R394X**	Dominant negative SOX9 mutations in campomelic dysplasia	Human Mutations	10.1002/humu.23888
**E400X**	Mutational analysis of the SOX9 gene in campomelic dysplasia and autosomal sex reversal: lack of genotype/phenotype correlations	Human Molecular genetics	https://doi.org/10.1093/hmg/6.1.91
**Q412X**	Dominant negative SOX9 mutations in campomelic dysplasia	Human Mutations	10.1002/humu.23888
**S438X**	Campomelic Dysplasia: airway management in two patients and an update on clinical molecular correlations in the head and neck	Annals of otology, rhinology & laryngology	10.1177/000348941112001009
**Y440X**	A homozygous nonsense mutation in SOX9 in the dominant disorder campomelic dysplasia: a case of mitotic gene conversion	Human genetics	10.1007/s00439-005-1295-y
**Y440X**	Mutational analysis of the SOX9 gene in campomelic dysplasia and autosomal sex reversal: lack of genotype/phenotype correlations	Human Molecular genetics	https://doi.org/10.1093/hmg/6.1.91
**Q458X**	Clinical, genetics and bioinformatics characterization of a campomelic dysplasia case report	Gene	https://doi.org/10.1016/j.gene.2015.11.039
**H65Y**	The phenotype of survivors of campomelic dysplasia	Journal of medical genetics	10.1136/jmg.39.8.597
**A76E**	Loss of DNA-dependent dimerization of the transcription factor SOX9 as a cause for campomelic dysplasia	Human Molecular genetics	https://doi.org/10.1093/hmg/ddg158
**Q79P**	The presence of diminished white matter and corpus callosal thinning in a case with a SOX9 mutation	Brain and Development	https://doi.org/10.1016/j.braindev.2017.09.002
**V80G**	Clinical Utility Gene Card for: campomelic dysplasia	European Journal of Medical Genetics	doi.org/10.1038/ejhg.2012.228
**P108L**	Mutational analysis of the SOX9 gene in campomelic dysplasia and autosomal sex reversal: lack of genotype/phenotype correlations	Human Molecular genetics	https://doi.org/10.1093/hmg/6.1.91
**F112S**	Dominant negative SOX9 mutations in campomelic dysplasia	Human Mutations	10.1002/humu.23888
**M113V**	A mutation creating an upstream initiation codon in the SOX9 5' UTR causes acampomelic campomelic dysplasia	Human Mutation	https://doi.org/10.1002/mgg3.282
**119 (C358)**	Familial Campomelic dysplasia due to maternal germinal mosaicism	Congenital Anomalies	10.1111/cga.12279
**A119V**	Campomelic dysplasia without sex reversal in a Turkish patient is due to mutation Ala119Val within the SOX9 gene	Clinical Dysmorphology	10.1097/00019605-200107000-00009
**W143R**	Mutational analysis of the SOX9 gene in campomelic dysplasia and autosomal sex reversal: lack of genotype/phenotype correlations	Human Molecular genetics	https://doi.org/10.1093/hmg/6.1.91
**R152P**	Mutational analysis of the SOX9 gene in campomelic dysplasia and autosomal sex reversal: lack of genotype/phenotype correlations	Human Molecular genetics	https://doi.org/10.1093/hmg/6.1.91
**F154L**	Compound Effects of Point Mutations Causing Campomelic Dysplasia/Autosomal Sex Reversal upon SOX9 Structure, Nuclear Transport, DNA Binding, and Transcriptional Activation	Journal of Biological Chemistry	https://doi.org/10.1074/jbc.M101278200
**A158T**	A case of campomelic dysplasia in whom a new mutation was found in the SOX9 gene	Turk pediatri arsivi	10.5152/tpa.2014.1187
**A158V**	Compound Effects of Point Mutations Causing Campomelic Dysplasia/Autosomal Sex Reversal upon SOX9 Structure, Nuclear Transport, DNA Binding, and Transcriptional Activation	Journal of Biological Chemistry	https://doi.org/10.1074/jbc.M101278200
**H165Q**	A mutation creating an upstream initiation codon in the SOX9 5' UTR causes acampomelic campomelic dysplasia	Human Mutation	https://doi.org/10.1002/mgg3.282
**H169Q**	A mutation creating an upstream initiation codon in the SOX9 5' UTR causes acampomelic campomelic dysplasia	Human Mutation	https://doi.org/10.1002/mgg3.282
**H169P**	Campomelic Dysplasia: echographic suspicion in the first trimester of pregnancy and final diagnosis of two cases	Fetal diagnosis and therapy	10.1159/000176299
**P170L**	Campomelic dysplasia: evidence of autosomal dominant inheritance	Journal of medical genetics	10.1136/jmg.30.8.683
**P170L**	Mild Campomelic Dysplasia: Report on a Case and Review	Molecular Syndromology	10.1159/000322861
**P170R**	Mutational analysis of the SOX9 gene in campomelic dysplasia and autosomal sex reversal: lack of genotype/phenotype correlations	Human Molecular genetics	https://doi.org/10.1093/hmg/6.1.91

Supplementary table 2. Variants found in the WES analysis

Chr	Start	End	Ref	Alt	Func.refGene	Gene.refGen	GeneDetail.r	ExonicFunc.r	AAChange.re
1	1238583	1238583	G	A	exonic	ACAP3	.	nonsynonym	ACAP3:NM_(
1	6638995	6638995	C	T	exonic	TAS1R1	.	nonsynonym	TAS1R1:NM_
1	26608843	26608843	C	A	exonic	UBXN11	.	nonsynonym	UBXN11:NM
1	26608852	26608852	G	A	exonic	UBXN11	.	nonsynonym	UBXN11:NM
1	26644537	26644537	C	A	exonic;splicir	CD52;UBXN1	.	nonsynonym	CD52:NM_00
1	27278439	27278439	G	A	exonic	KDF1	.	nonsynonym	KDF1:NM_15
1	36638206	36638206	G	A	exonic	MAP7D1	.	nonsynonym	MAP7D1:NM
1	40928731	40928731	G	A	exonic	ZFP69B	.	nonsynonym	ZFP69B:NM_
1	46739296	46739296	G	C	exonic	RAD54L	.	nonsynonym	RAD54L:NM_
1	113010178	113010178	G	A	exonic	WNT2B	.	nonsynonym	WNT2B:NM_
1	113058941	113058941	G	A	exonic	WNT2B	.	nonsynonym	WNT2B:NM_
1	115223010	115223010	C	T	exonic	AMPD1	.	nonsynonym	AMPD1:NM_
1	152129115	152129115	T	C	exonic	RPTN	.	nonsynonym	RPTN:NM_0(
1	152275453	152275453	G	A	exonic	FLG	.	nonsynonym	FLG:NM_002
1	152883786	152883786	C	G	exonic	IVL	.	nonsynonym	IVL:NM_005!
1	156012575	156012575	A	C	exonic	UBQLN4	.	nonsynonym	UBQLN4:NM
1	156708367	156708367	C	T	exonic	MRPL24	.	nonsynonym	MRPL24:NM
1	158687308	158687308	C	T	exonic	OR6K3	.	nonsynonym	OR6K3:NM_(
1	168698298	168698298	C	T	exonic	DPT	.	nonsynonym	DPT:NM_001
1	177906507	177906507	G	A	exonic	SEC16B	.	nonsynonym	SEC16B:NM_
1	179783184	179783184	C	T	exonic	FAM163A	.	nonsynonym	FAM163A:NI
1	186330835	186330835	C	T	exonic	TPR	.	nonsynonym	TPR:NM_003
1	205128792	205128792	A	C	exonic	DSTYK	.	nonsynonym	DSTYK:NM_(
1	212798251	212798251	C	T	exonic	FAM71A	.	nonsynonym	FAM71A:NM
1	225332296	225332296	C	G	exonic	DNAH14	.	nonsynonym	DNAH14:NM
1	226420879	226420879	C	T	exonic	LIN9	.	nonsynonym	LIN9:NM_00
1	226924196	226924196	C	T	exonic	ITPKB	.	nonsynonym	ITPKB:NM_0(
1	247151164	247151164	C	A	exonic	ZNF695	.	nonsynonym	ZNF695:NM_
1	247836078	247836078	A	C	exonic	OR13G1	.	nonsynonym	OR13G1:NM
1	248525100	248525100	G	A	exonic	OR2T4	.	nonsynonym	OR2T4:NM_(
2	1652964	1652964	G	A	exonic	PXDN	.	nonsynonym	PXDN:NM_0:
2	23865369	23865369	C	G	exonic	KLHL29	.	nonsynonym	KLHL29:NM_

2	46707808	46707808	C	G	exonic	TMEM247	.	nonsynonym	TMEM247:NI
2	61412677	61412677	C	G	exonic	AHSA2	.	nonsynonym	AHSA2:NM_:
2	69732709	69732709	G	A	exonic	AAK1	.	nonsynonym	AAK1:NM_01
2	71896796	71896796	G	A	exonic	DYSF	.	nonsynonym	DYSF:NM_00
2	110962532	110962532	C	A	exonic	NPHP1	.	nonsynonym	NPHP1:NM_(
2	125204473	125204473	C	T	exonic	CNTNAP5	.	nonsynonym	CNTNAP5:NI
2	152321090	152321090	C	G	exonic	RIF1	.	nonsynonym	RIF1:NM_00
2	152499355	152499355	T	C	exonic	NEB	.	nonsynonym	NEB:NM_00:
2	167300128	167300128	T	C	exonic	SCN7A	.	nonsynonym	SCN7A:NM_(
2	170350312	170350312	A	G	exonic	BBS5	.	nonsynonym	BBS5:NM_15
2	172325530	172325530	A	G	exonic	DCAF17	.	nonsynonym	DCAF17:NM_
2	179309165	179309165	G	A	exonic	PRKRA	.	nonsynonym	PRKRA:NM_(
2	179497018	179497018	G	A	exonic	TTN	.	nonsynonym	TTN:NM_003
2	189912958	189912958	G	A	exonic	COL5A2	.	nonsynonym	COL5A2:NM_
2	190313105	190313105	G	T	exonic	WDR75	.	nonsynonym	WDR75:NM_
2	196765098	196765098	G	A	exonic	DNAH7	.	nonsynonym	DNAH7:NM_
2	197208434	197208434	G	A	exonic	HECW2	.	nonsynonym	HECW2:NM_
2	220047173	220047173	A	T	exonic	FAM134A	.	nonsynonym	FAM134A:NI
2	233127939	233127939	G	A	exonic	DIS3L2	.	nonsynonym	DIS3L2:NM_(
2	241069329	241069329	T	C	exonic	MYEOV2	.	nonsynonym	MYEOV2:NM
3	9833071	9833071	G	A	exonic	TADA3	.	nonsynonym	TADA3:NM_(
3	32804254	32804254	C	T	exonic	CNOT10	.	nonsynonym	CNOT10:NM_
3	63976489	63976489	G	A	exonic	ATXN7	.	nonsynonym	ATXN7:NM_(
3	81754732	81754732	A	G	exonic	GBE1	.	nonsynonym	GBE1:NM_00
3	97311483	97311483	C	T	exonic	EPHA6	.	nonsynonym	EPHA6:NM_(
3	113528243	113528243	T	C	exonic	ATP6V1A	.	nonsynonym	ATP6V1A:NM
3	124896625	124896625	A	G	exonic	SLC12A8	.	nonsynonym	SLC12A8:NM
3	185993354	185993354	G	C	exonic	DGKG	.	nonsynonym	DGKG:NM_0
3	185993355	185993355	C	G	exonic	DGKG	.	nonsynonym	DGKG:NM_0
3	195516350	195516350	C	T	exonic	MUC4	.	nonsynonym	MUC4:NM_0
3	196743994	196743994	G	A	exonic	MFI2	.	nonsynonym	MFI2:NM_00
4	59408	59408	G	C	exonic	ZNF595,ZNF	.	nonsynonym	ZNF718:NM_
4	1388324	1388324	A	C	exonic	CRIPAK	.	nonsynonym	CRIPAK:NM_

4	2306696	2306696	G	T	exonic	ZFYVE28	.	nonsynonym	ZFYVE28:NM
4	3494917	3494917	C	T	exonic	DOK7	.	nonsynonym	DOK7:NM_0(
4	3534030	3534030	C	G	exonic	LRPAP1	.	nonsynonym	LRPAP1:NM_
4	8588929	8588929	C	T	exonic	GPR78	.	nonsynonym	GPR78:NM_(
4	13604488	13604488	C	G	exonic	BOD1L1	.	nonsynonym	BOD1L1:NM_
4	42658856	42658856	C	A	exonic	ATP8A1	.	nonsynonym	ATP8A1:NM_
4	102783698	102783698	T	G	exonic	BANK1	.	nonsynonym	BANK1:NM_(
4	108969883	108969883	G	A	exonic	LEF1	.	nonsynonym	LEF1:NM_00
4	169799408	169799408	G	C	exonic	PALLD	.	nonsynonym	PALLD:NM_0
5	620185	620185	G	A	exonic	CEP72	.	nonsynonym	CEP72:NM_0
5	32074181	32074181	C	T	exonic	PDZD2	.	nonsynonym	PDZD2:NM_1
5	53815191	53815191	C	G	exonic	SNX18	.	nonsynonym	SNX18:NM_(
5	74807116	74807116	C	T	exonic	COL4A3BP	.	nonsynonym	COL4A3BP:N
5	140250471	140250471	G	T	exonic	PCDHA11	.	nonsynonym	PCDHA11:NM
5	140731938	140731938	T	A	exonic	PCDHGB1	.	nonsynonym	PCDHGB1:NM
5	141052423	141052423	C	G	exonic	ARAP3	.	nonsynonym	ARAP3:NM_(
5	148207169	148207169	C	T	exonic	ADRB2	.	nonsynonym	ADRB2:NM_(
5	170738596	170738596	T	A	exonic	TLX3	.	nonsynonym	TLX3:NM_02
5	176072412	176072412	A	C	exonic	EIF4E1B	.	nonsynonym	EIF4E1B:NM_
5	176951713	176951713	A	G	exonic	FAM193B	.	nonsynonym	FAM193B:NM
6	26392629	26392629	G	A	exonic	BTN2A2	.	nonsynonym	BTN2A2:NM_
6	28228209	28228209	C	G	exonic	NKAPL	.	nonsynonym	NKAPL:NM_(
6	29364508	29364508	T	C	exonic	OR12D2	.	nonsynonym	OR12D2:NM_
6	30164467	30164467	C	A	exonic	TRIM26	.	nonsynonym	TRIM26:NM_
6	31750533	31750533	G	A	exonic	VARS	.	nonsynonym	VARS:NM_0(
6	31778432	31778432	C	A	exonic	HSPA1L	.	nonsynonym	HSPA1L:NM_
6	32015775	32015775	G	A	exonic	TNXB	.	nonsynonym	TNXB:NM_01
6	33385438	33385438	G	A	exonic	CUTA	.	nonsynonym	CUTA:NM_01
6	90348407	90348407	G	T	exonic	LYRM2	.	nonsynonym	LYRM2:NM_(
6	159398871	159398871	C	T	exonic	RSPH3	.	nonsynonym	RSPH3:NM_(
7	2751987	2751987	G	A	exonic	AMZ1	.	nonsynonym	AMZ1:NM_0
7	21778438	21778438	G	A	exonic	DNAH11	.	nonsynonym	DNAH11:NM_
7	87517349	87517349	A	C	exonic	DBF4	.	nonsynonym	DBF4:NM_0(

7	94897945	94897945	C	T	exonic	PPP1R9A	.	nonsynonym	PPP1R9A:NM
7	97842022	97842022	T	C	exonic	BHLHA15	.	nonsynonym	BHLHA15:NM
7	98449130	98449130	T	G	exonic	TMEM130	.	nonsynonym	TMEM130:N
7	99474158	99474158	G	T	exonic	OR2AE1	.	nonsynonym	OR2AE1:NM_
7	129818350	129818350	A	C	exonic	TMEM209	.	nonsynonym	TMEM209:N
7	129917678	129917678	C	T	exonic	CPA2	.	nonsynonym	CPA2:NM_00
7	130008374	130008374	C	T	exonic	CPA5	.	nonsynonym	CPA5:NM_00
7	134678273	134678273	C	T	exonic	AGBL3	.	nonsynonym	AGBL3:NM_
7	150883671	150883671	A	G	exonic	ASB10	.	nonsynonym	ASB10:NM_
7	157985138	157985138	C	T	exonic	PTPRN2	.	nonsynonym	PTPRN2:NM_
8	10480420	10480420	C	T	exonic	RP1L1	.	nonsynonym	RP1L1:NM_1
8	16035422	16035422	G	A	exonic	MSR1	.	nonsynonym	MSR1:NM_0
8	21996546	21996546	C	T	exonic	REEP4	.	nonsynonym	REEP4:NM_
8	22006170	22006170	C	T	exonic	LGI3	.	nonsynonym	LGI3:NM_13
8	38369926	38369926	A	C	exonic	C8orf86	.	nonsynonym	C8orf86:NM_
8	42231859	42231859	C	T	exonic	DKK4	.	nonsynonym	DKK4:NM_01
8	53586746	53586746	C	T	exonic	RB1CC1	.	nonsynonym	RB1CC1:NM_
8	101608953	101608953	T	G	exonic	SNX31	.	nonsynonym	SNX31:NM_1
8	113299353	113299353	A	G	exonic	CSMD3	.	nonsynonym	CSMD3:NM_
8	125103719	125103719	C	A	exonic	FER1L6	.	nonsynonym	FER1L6:NM_
8	144408427	144408427	C	A	exonic	TOP1MT	.	nonsynonym	TOP1MT:NM
8	144650859	144650859	C	G	exonic	MROH6	.	nonsynonym	MROH6:NM_
9	12821567	12821567	C	A	exonic	LURAP1L	.	nonsynonym	LURAP1L:NM
9	13110045	13110045	C	T	exonic	MPDZ	.	nonsynonym	MPDZ:NM_0
9	35906601	35906601	C	A	exonic	HRCT1	.	nonsynonym	HRCT1:NM_
9	35906604	35906604	A	C	exonic	HRCT1	.	nonsynonym	HRCT1:NM_
9	98229473	98229473	C	T	exonic	PTCH1	.	nonsynonym	PTCH1:NM_
9	127618856	127618856	T	G	exonic	WDR38	.	nonsynonym	WDR38:NM_
9	139734846	139734846	C	T	exonic	RABL6	.	nonsynonym	RABL6:NM_
9	139747787	139747787	G	A	exonic	MAMDC4	.	nonsynonym	MAMDC4:NM
10	1230874	1230874	C	T	exonic	ADARB2	.	nonsynonym	ADARB2:NM
10	23393091	23393091	A	G	exonic	MSRB2	.	nonsynonym	MSRB2:NM_
10	26851284	26851284	G	A	exonic	APBB1IP	.	nonsynonym	APBB1IP:NM

10	31134425	31134425	C	T	exonic	ZNF438	.	nonsynonym	ZNF438:NM_
10	70652345	70652345	G	A	exonic	STOX1	.	nonsynonym	STOX1:NM_(
10	79603320	79603320	G	A	exonic	DLG5	.	nonsynonym	DLG5:NM_00
10	88476509	88476509	C	A	exonic	LDB3	.	nonsynonym	LDB3:NM_00
10	102057199	102057199	C	G	exonic	PKD2L1	.	nonsynonym	PKD2L1:NM_
10	105794495	105794495	G	A	exonic	COL17A1	.	nonsynonym	COL17A1:NM
10	105819405	105819405	C	T	exonic	COL17A1	.	nonsynonym	COL17A1:NM
10	118969560	118969560	C	T	exonic	KCNK18	.	nonsynonym	KCNK18:NM_
10	128974238	128974238	T	C	exonic	FAM196A	.	nonsynonym	FAM196A:NI
11	803537	803537	T	G	exonic	PIDD1	.	nonsynonym	PIDD1:NM_1
11	2170560	2170560	A	T	exonic	INS-IGF2	.	nonsynonym	INS-IGF2:NM
11	5510540	5510540	G	A	exonic	OR52D1	.	nonsynonym	OR52D1:NM_
11	5536386	5536386	A	G	exonic	UBQLNL	.	nonsynonym	UBQLNL:NM
11	7021374	7021374	G	A	exonic	ZNF214	.	nonsynonym	ZNF214:NM_
11	18729488	18729488	C	T	exonic	IGSF22	.	nonsynonym	IGSF22:NM_
11	59573999	59573999	C	T	exonic	MRPL16	.	nonsynonym	MRPL16:NM_
11	66834232	66834232	C	T	exonic	RHOD	.	nonsynonym	RHOD:NM_0
11	73679454	73679454	G	A	exonic	DNAJB13	.	nonsynonym	DNAJB13:NM
11	96104223	96104223	C	T	exonic	CCDC82	.	nonsynonym	CCDC82:NM_
11	100998343	100998343	C	A	exonic	PGR	.	nonsynonym	PGR:NM_00(
11	118307385	118307385	C	T	exonic	KMT2A	.	nonsynonym	KMT2A:NM_
11	124180356	124180356	A	G	exonic	OR8D1	.	nonsynonym	OR8D1:NM_(
11	130332457	130332457	T	C	exonic	ADAMTS15	.	nonsynonym	ADAMTS15:N
11	134122759	134122759	C	A	exonic	THYN1	.	nonsynonym	THYN1:NM_(
11	134238603	134238603	G	A	exonic	GLB1L2	.	nonsynonym	GLB1L2:NM_
12	1963174	1963174	G	A	exonic	CACNA2D4	.	nonsynonym	CACNA2D4:N
12	6729534	6729534	G	T	exonic	LPAR5	.	nonsynonym	LPAR5:NM_(
12	51323835	51323835	G	A	exonic	METTL7A	.	nonsynonym	METTL7A:NN
12	56349632	56349632	G	C	exonic	PMEL	.	nonsynonym	PMEL:NM_0(
12	56743044	56743044	G	A	exonic	STAT2	.	nonsynonym	STAT2:NM_(
12	57433051	57433051	G	A	exonic	MYO1A	.	nonsynonym	MYO1A:NM_
12	88439500	88439500	A	G	exonic	C12orf29	.	nonsynonym	C12orf29:NM
12	104144426	104144426	C	T	exonic	STAB2	.	nonsynonym	STAB2:NM_(

12	108145285	108145285	C	T	exonic	PRDM4	.	nonsynonym	PRDM4:NM_
12	113875765	113875765	C	A	exonic	SDSL	.	nonsynonym	SDSL:NM_00
13	33590828	33590828	G	C	exonic	KL	.	nonsynonym	KL:NM_0047
13	114762038	114762038	C	T	exonic	RASA3	.	nonsynonym	RASA3:NM_(
14	23612372	23612372	T	G	exonic	SLC7A8	.	nonsynonym	SLC7A8:NM_
14	55604883	55604883	G	A	exonic	LGALS3	.	nonsynonym	LGALS3:NM_
14	60712590	60712590	G	C	exonic	PPM1A	.	nonsynonym	PPM1A:NM_
14	68251815	68251815	G	A	exonic	ZFYVE26	.	nonsynonym	ZFYVE26:NM
14	91779480	91779480	G	A	exonic	CCDC88C	.	nonsynonym	CCDC88C:NM
14	93392986	93392986	C	A	exonic	CHGA	.	nonsynonym	CHGA:NM_0(
14	96768380	96768380	T	G	exonic	ATG2B	.	nonsynonym	ATG2B:NM_(
15	42978426	42978426	T	A	exonic	STARD9	.	nonsynonym	STARD9:NM_
15	43494153	43494153	T	C	exonic	EPB42	.	nonsynonym	EPB42:NM_(
15	43739672	43739672	G	T	exonic	TP53BP1	.	nonsynonym	TP53BP1:NM
15	45439691	45439691	C	T	exonic	DUOX1	.	nonsynonym	DUOX1:NM_
15	59186663	59186663	T	C	exonic	SLTM	.	nonsynonym	SLTM:NM_0(
15	72191110	72191110	T	C	exonic	MYO9A	.	nonsynonym	MYO9A:NM_
15	85525523	85525523	C	T	exonic	PDE8A	.	nonsynonym	PDE8A:NM_(
15	86311316	86311316	T	C	exonic	KLHL25	.	nonsynonym	KLHL25:NM_
15	86697675	86697675	C	T	exonic	AGBL1	.	nonsynonym	AGBL1:NM_1
16	1536304	1536304	C	T	exonic	PTX4	.	nonsynonym	PTX4:NM_00
16	3081049	3081049	G	A	exonic	CCDC64B	.	nonsynonym	CCDC64B:NM
16	3613275	3613275	G	A	exonic	NLRC3	.	nonsynonym	NLRC3:NM_1
16	3708834	3708834	C	T	exonic	TRAP1	.	nonsynonym	TRAP1:NM_(
16	18853620	18853620	C	T	exonic	SMG1	.	nonsynonym	SMG1:NM_0
16	19083300	19083300	G	C	exonic	COQ7	.	nonsynonym	COQ7:NM_0(
16	27476768	27476768	T	C	exonic	GTF3C1	.	nonsynonym	GTF3C1:NM_
16	28962631	28962631	C	T	exonic	NFATC2IP	.	nonsynonym	NFATC2IP:NI
16	29853091	29853091	C	T	exonic	MVP	.	nonsynonym	MVP:NM_00
16	30017797	30017797	C	T	exonic	DOC2A	.	nonsynonym	DOC2A:NM_(
16	30748932	30748932	C	G	exonic	SRCAP	.	nonsynonym	SRCAP:NM_(
16	31384676	31384676	G	A	exonic	ITGAX	.	nonsynonym	ITGAX:NM_0
16	57758631	57758631	A	G	exonic	DRC7	.	nonsynonym	DRC7:NM_0(

16	58018231	58018231	C	T	exonic	TEPP	.	nonsynonym	TEPP:NM_19
16	72993725	72993725	G	A	exonic	ZFHX3	.	nonsynonym	ZFHX3:NM_0
16	81942175	81942175	A	G	exonic	PLCG2	.	nonsynonym	PLCG2:NM_0
17	1538083	1538083	A	G	exonic	SCARF1	.	nonsynonym	SCARF1:NM_
17	5425076	5425076	A	G	exonic	NLRP1	.	nonsynonym	NLRP1:NM_0
17	7750903	7750903	T	C	exonic	KDM6B	.	nonsynonym	KDM6B:NM_
17	8280923	8280923	C	T	exonic	RPL26	.	nonsynonym	RPL26:NM_0
17	16248013	16248013	T	C	exonic	CENPV	.	nonsynonym	CENPV:NM_1
17	38061072	38061072	G	C	exonic	GSDMB	.	nonsynonym	GSDMB:NM_
17	38643384	38643384	C	A	exonic	TNS4	.	nonsynonym	TNS4:NM_03
17	43923990	43923990	A	G	exonic	SPPL2C	.	nonsynonym	SPPL2C:NM_
17	48750431	48750431	G	A	exonic	ABCC3	.	nonsynonym	ABCC3:NM_0
17	48917355	48917355	C	T	exonic	WFIKKN2	.	nonsynonym	WFIKKN2:NM
17	62121542	62121542	G	T	exonic	ERN1	.	nonsynonym	ERN1:NM_00
17	73498709	73498709	C	T	exonic	CASKIN2	.	nonsynonym	CASKIN2:NM
17	73733712	73733712	G	T	exonic	ITGB4	.	nonsynonym	ITGB4:NM_0
17	74475837	74475837	A	G	exonic	RHBDF2	.	nonsynonym	RHBDF2:NM_
18	3071878	3071878	C	T	exonic	MYOM1	.	nonsynonym	MYOM1:NM_
18	7011475	7011475	T	C	exonic	LAMA1	.	nonsynonym	LAMA1:NM_
18	13826119	13826119	G	T	exonic	MC5R	.	nonsynonym	MC5R:NM_0
18	13884803	13884803	C	T	exonic	MC2R	.	nonsynonym	MC2R:NM_0
18	21946887	21946887	G	A	exonic	OSBPL1A	.	nonsynonym	OSBPL1A:NM
18	28908178	28908178	G	C	exonic	DSG1	.	nonsynonym	DSG1:NM_00
18	44774764	44774764	A	C	exonic	SKOR2	.	nonsynonym	SKOR2:NM_0
18	50683852	50683852	C	T	exonic	DCC	.	nonsynonym	DCC:NM_005
19	1567630	1567630	G	A	exonic	MEX3D	.	nonsynonym	MEX3D:NM_
19	7569091	7569091	C	G	exonic	C19orf45	.	nonsynonym	C19orf45:NM
19	7697712	7697712	C	A	exonic	PCP2	.	nonsynonym	PCP2:NM_00
19	7830938	7830938	A	G	exonic;splicir	CLEC4M;CLE(	NM_001144!	nonsynonym	CLEC4M:NM_
19	8429328	8429328	G	C	exonic	ANGPTL4	.	nonsynonym	ANGPTL4:NM
19	9072130	9072130	C	G	exonic	MUC16	.	nonsynonym	MUC16:NM_
19	18994936	18994936	C	T	exonic	CERS1	.	nonsynonym	CERS1:NM_0
19	34003674	34003674	A	G	exonic	PEPD	.	nonsynonym	PEPD:NM_00

19	38817359	38817359	C	T	exonic	KCNK6	.	nonsynonym	KCNK6:NM_(
19	40225031	40225031	G	C	exonic	CLC	.	nonsynonym	CLC:NM_001
19	46394095	46394095	G	T	exonic	MYPOP	.	nonsynonym	MYPOP:NM_
19	49002627	49002627	G	T	exonic	LMTK3	.	nonsynonym	LMTK3:NM_(
19	53116949	53116949	T	C	exonic	ZNF83	.	nonsynonym	ZNF83:NM_(
19	53432449	53432449	C	G	exonic	ZNF816-ZNF	.	nonsynonym	ZNF816-ZNF
19	56090524	56090524	G	A	exonic	ZNF579	.	nonsynonym	ZNF579:NM_
19	58639949	58639949	T	C	exonic	ZNF329	.	nonsynonym	ZNF329:NM_
20	23433441	23433441	G	A	exonic	CST11	.	nonsynonym	CST11:NM_0
20	35467706	35467706	G	A	exonic	SOGA1	.	nonsynonym	SOGA1:NM_
20	36775191	36775191	G	A	exonic	TGM2	.	nonsynonym	TGM2:NM_0
20	40040870	40040870	G	A	exonic	CHD6	.	nonsynonym	CHD6:NM_03
20	44047974	44047974	G	A	exonic	PIGT	.	nonsynonym	PIGT:NM_00
20	56227354	56227354	G	C	exonic	PMEPA1	.	nonsynonym	PMEPA1:NM
20	60992295	60992295	C	T	exonic	RBBP8NL	.	nonsynonym	RBBP8NL:NM
20	62196648	62196648	A	G	exonic	HELZ2	.	nonsynonym	HELZ2:NM_0
21	47848349	47848349	C	T	exonic	PCNT	.	nonsynonym	PCNT:NM_00
22	23503155	23503155	C	T	exonic	RAB36	.	nonsynonym	RAB36:NM_(
22	29627111	29627111	G	A	exonic	EMID1	.	nonsynonym	EMID1:NM_(
22	29656165	29656165	T	C	exonic	RHBDD3	.	nonsynonym	RHBDD3:NM
22	29682974	29682974	G	C	exonic	EWSR1	.	nonsynonym	EWSR1:NM_
22	30685380	30685380	C	A	exonic	GATSL3	.	nonsynonym	GATSL3:NM_
22	32000930	32000930	C	T	exonic	SFI1	.	nonsynonym	SFI1:NM_001
22	39067128	39067128	C	T	exonic	CBY1	.	nonsynonym	CBY1:NM_01
22	41605776	41605776	G	C	exonic	L3MBTL2	.	nonsynonym	L3MBTL2:NM
22	42606388	42606388	C	G	exonic	TCF20	.	nonsynonym	TCF20:NM_1
22	50716045	50716045	G	A	exonic	PLXNB2	.	nonsynonym	PLXNB2:NM_
X	2835863	2835863	G	T	exonic	ARSD	.	nonsynonym	ARSD:NM_00
X	2836181	2836181	A	T	exonic	ARSD	.	nonsynonym	ARSD:NM_00
X	2836184	2836184	C	T	exonic	ARSD	.	nonsynonym	ARSD:NM_00

cytoBand	genomicSupe	CLINSIG	CLNDBN	CLNACC	CLNDSDB	CLNDSDBID	gnomAD_gei	gnomAD_gei	gnomAD_gei
1p36.33	.	.	.	.	.	.	0.0059	0.003	0.0383
1p36.31	.	.	.	.	.	.	0.0009	0.0003	0.0155
1p36.11	.	.	.	.	.	.	0.0041	0.0098	0.0015
1p36.11	.	.	.	.	.	.	0.00003936	0	0
1p36.11	.	.	.	.	.	.	0.0082	0.0033	0.1456
1p36.11	.	.	.	.	.	.	0.0022	0.0007	0.068
1p34.3	.	.	.	.	.	.	0.0059	0.0016	0.1695
1p34.2	.	.	.	.	.	.	0.0055	0.0017	0.1551
1p34.1	.	.	.	.	.	.	0.0002	0	0.0048
1p13.2	.	.	.	.	.	.	0.0061	0.0037	0.1742
1p13.2	.	.	.	.	.	.	0	0	0
1p13.2	.	.	.	.	.	.	0.0001	0.0003	0
1q21.3	.	.	.	.	.	.	0	0	0
1q21.3	.	.	.	.	.	.	0.0065	0.0031	0.0597
1q21.3	.	.	.	.	.	.	0.0029	0.0015	0.0752
1q22	.	.	.	.	.	.	0	0	0
1q23.1	.	.	.	.	.	.	0.0021	0.0075	0
1q23.1	.	.	.	.	.	.	0.0044	0.0154	0
1q24.2	.	.	.	.	.	.	0.003	0.0011	0.0907
1q25.2	.	.	.	.	.	.	0.0076	0.0263	0.0012
1q25.2	.	.	.	.	.	.	0	0	0
1q31.1	.	.	.	.	.	.	0	0	0
1q32.1	.	.	.	.	.	.	0	0	0
1q32.3	.	.	.	.	.	.	0.0056	0.0022	0.0728
1q42.12	.	.	.	.	.	.	0.0011	0.0003	0.0334
1q42.12	.	.	.	.	.	.	0	0	0
1q42.12	.	.	.	.	.	.	0.0072	0.0015	0.0837
1q44	.	.	.	.	.	.	0	0	0
1q44	.	.	.	.	.	.	0.0049	0.0174	0
1q44	.	.	.	.	.	.	0.0008	0.0011	0
2p25.3	.	.	.	.	.	.	0	0	0
2p24.1	.	.	.	.	.	.	0.0017	0.0008	0.0191

2p21	.	.	.	.	.	.	0	0	0
2p15	.	.	.	.	.	.	0.0018	0.0013	0.0538
2p13.3	.	.	.	.	.	.	0	0	0
2p13.2	.	Uncertain sig	not_specifie	RCV0002691	MedGen	CN169374	0	0	0
2q13	.	other|other|	not_specifie	RCV0001178	MedGen|Me	CN169374|C	0.0015	0.0008	0.043
2q14.3	.	.	.	.	.	.	0	0	0
2q23.3	.	.	.	.	.	.	0.0038	0.0002	0.0095
2q23.3	.	other|Likely	not_specifie	RCV0001177	MedGen|Me	CN169374|C	0.0066	0.0002	0.0084
2q24.3	.	.	.	.	.	.	0.0021	0.0008	0.037
2q31.1	.	other	not_specifie	RCV0002494	MedGen	CN169374	0.0015	0.0006	0.043
2q31.1	.	.	.	.	.	.	0	0	0
2q31.2	.	Likely benign	Dystonia	RCV0003904	MedGen	C0393593	0	0	0
2q31.2	.	other|Likely	not_specifie	RCV0000402	MedGen|Me	CN169374|C	0.0047	0.0028	0.1325
2q32.2	.	Uncertain sig	not_specifie	RCV0001975	MedGen|Me	CN169374|C	0.00009687	0.0002	0
2q32.2	.	.	.	.	.	.	0	0	0
2q32.3	.	.	.	.	.	.	0.003	0.0002	0.0322
2q32.3	.	.	.	.	.	.	0.0008	0.0002	0.0263
2q35	.	.	.	.	.	.	0.0057	0.002	0.0847
2q37.1	.	Benign|Likel	not_specifie	RCV0002518	MedGen|Me	CN169374|C	0.0056	0.001	0.0562
2q37.3	.	.	.	.	.	.	0	0	0
3p25.3	.	.	.	.	.	.	0	0	0
3p22.3	.	.	.	.	.	.	0	0	0
3p14.1	.	.	.	.	.	.	0.0061	0.0016	0.0979
3p12.2	.	Benign|Likel	not_specifie	RCV0002510	MedGen|Me	CN169374|C	0.0056	0.0027	0.0132
3q11.2	.	.	.	.	.	.	0.0083	0.0024	0.1289
3q13.31	.	.	.	.	.	.	0	0	0
3q21.2	.	.	.	.	.	.	0.0023	0.0006	0.0119
3q27.2	.	.	.	.	.	.	0	0	0
3q27.2	.	.	.	.	.	.	0	0	0
3q29	.	.	.	.	.	.	0.0093	0.0046	0.238
3q29	.	.	.	.	.	.	0.0056	0.0019	0.037
4p16.3	.	.	.	.	.	.	0	0	0
4p16.3	.	.	.	.	.	.	0	0	0

4p16.3	.	.	.	.	.	.	0.007	0.0246	0.0012
4p16.3	.	Benign	not_specifie	RCV0002497	MedGen	CN169374	0.0051	0.017	0.0012
4p16.3	.	.	.	.	.	.	0.0085	0.0301	0
4p16.1	.	.	.	.	.	.	0.0002	0.0002	0.0036
4p15.33	.	.	.	.	.	.	0	0	0
4p13	.	.	.	.	.	.	0	0	0
4q24	.	.	.	.	.	.	0.007	0.0234	0.006
4q25	.	.	.	.	.	.	0.0073	0.0021	0.1492
4q32.3	.	Uncertain sig	Pancreatic_a	RCV0001231	Human_Pher	HP:0006725:	0.0014	0.0001	0.0096
5p15.33	.	.	.	.	.	.	0	0	0
5p13.3	.	.	.	.	.	.	0.0022	0.0028	0.037
5q11.2	.	.	.	.	.	.	0.0014	0.0003	0.0395
5q13.3	.	.	.	.	.	.	0.0021	0.0001	0.0407
5q31.3	.	.	.	.	.	.	0.0031	0.001	0.0203
5q31.3	.	.	.	.	.	.	0.00003229	0	0.0012
5q31.3	.	.	.	.	.	.	0	0	0
5q32	.	.	.	.	.	.	0	0	0
5q35.1	.	.	.	.	.	.	0	0	0
5q35.2	.	.	.	.	.	.	0.0003	0.0011	0
5q35.3	.	.	.	.	.	.	0.0019	0.0064	0.0012
6p22.2	.	.	.	.	.	.	0.009	0.0036	0.0179
6p22.1	.	.	.	.	.	.	0.002	0.0007	0.0537
6p22.1	.	.	.	.	.	.	0.0039	0.0006	0.0764
6p22.1	.	.	.	.	.	.	0.0072	0.0016	0.1181
6p21.33	.	.	.	.	.	.	0	0	0
6p21.33	.	.	.	.	.	.	0	0	0
6p21.33	.	.	.	.	.	.	0.0017	0.0013	0.0383
6p21.32	.	.	.	.	.	.	0	0	0
6q15	.	.	.	.	.	.	0.0045	0.0028	0.0764
6q25.3	.	.	.	.	.	.	0.0024	0.001	0.074
7p22.3	.	.	.	.	.	.	0.0099	0.0344	0.0024
7p15.3	.	Likely benign	not_specifie	RCV0002474	MedGen|Me	CN169374|C	0.002	0.0068	0
7q21.12	.	.	.	.	.	.	0.0004	0	0.0107

7q21.3	.	.	.	.	.	.	0.0006	0.0005	0.0131
7q21.3	.	.	.	.	.	.	0	0	0
7q22.1	.	.	.	.	.	.	0.0049	0.0016	0.037
7q22.1	.	.	.	.	.	.	0.0099	0.0303	0.0286
7q32.2	.	.	.	.	.	.	0.0032	0.0008	0.0406
7q32.2	.	.	.	.	.	.	0.0027	0.0003	0.0096
7q32.2	.	.	.	.	.	.	0.0007	0.0003	0.0215
7q33	.	.	.	.	.	.	0.002	0.0011	0.0549
7q36.1	.	.	.	.	.	.	0.0004	0.0002	0.0095
7q36.3	.	.	.	.	.	.	0.0019	0.0064	0.0012
8p23.1	.	.	.	.	.	.	0.000097	0.0001	0
8p22	.	.	.	.	.	.	0.00009699	0	0
8p21.3	.	.	.	.	.	.	0.0028	0.0003	0.0215
8p21.3	.	.	.	.	.	.	0.0026	0.0013	0.0716
8p11.22	.	.	.	.	.	.	0.0076	0.0009	0.0525
8p11.21	.	.	.	.	.	.	0	0	0
8q11.23	.	.	.	.	.	.	0.0036	0.0013	0.0644
8q22.3	.	.	.	.	.	.	0	0	0
8q23.3	.	.	.	.	.	.	0.0042	0.0022	0.1146
8q24.13	.	.	.	.	.	.	0.0007	0.0002	0
8q24.3	.	.	.	.	.	.	0	0	0
8q24.3	.	.	.	.	.	.	0.0022	0.0009	0.0668
9p23	.	.	.	.	.	.	0.00006457	0.0001	0.0012
9p23	.	.	.	.	.	.	0	0	0
9p13.3	.	.	.	.	.	.	0	0	0
9p13.3	.	.	.	.	.	.	0.0028	0.0053	0.0247
9q22.32	.	Uncertain sig	not_providec	RCV0000345	MedGen|Me	CN221809|C	0.0002	0	0
9q33.3	.	.	.	.	.	.	0.0044	0.0023	0.1002
9q34.3	.	.	.	.	.	.	0.00003245	0	0
9q34.3	.	.	.	.	.	.	0.00009699	0	0
10p15.3	.	.	.	.	.	.	0.0025	0.0083	0
10p12.2	.	.	.	.	.	.	0.01	0.0003	0.0394
10p12.1	.	.	.	.	.	.	0	0	0

10p11.23	.	.	.	.	.	.	0.0052	0.0021	0.1611
10q22.1	.	.	.	.	.	.	0.0008	0.0005	0.0191
10q22.3	.	.	.	.	.	.	0	0	0
10q23.2	.	.	.	.	.	.	0	0	0
10q24.31	.	.	.	.	.	.	0.0002	0.0002	0.0036
10q24.33	.	.	.	.	.	.	0	0	0
10q25.1	.	.	.	.	.	.	0.00009684	0.0001	0
10q25.3	.	.	.	.	.	.	0	0	0
10q26.2	.	.	.	.	.	.	0	0	0
11p15.5	.	.	.	.	.	.	0	0	0
11p15.5	.	.	.	.	.	.	0	0	0
11p15.4	.	.	.	.	.	.	0.0018	0.0009	0.0573
11p15.4	.	.	.	.	.	.	0.0076	0.0011	0.1205
11p15.4	.	.	.	.	.	.	0.0051	0.0019	0.14
11p15.1	.	.	.	.	.	.	0.0038	0.0013	0.0036
11q12.1	.	.	.	.	.	.	0.0048	0.0015	0.0656
11q13.2	.	.	.	.	.	.	0.0092	0.0014	0.0119
11q13.4	.	.	.	.	.	.	0.003	0.0009	0.0012
11q21	.	.	.	.	.	.	0	0	0
11q22.1	.	.	.	.	.	.	0	0	0
11q23.3	.	Benign	not_specifie	RCV0002393	MedGen	CN169374	0.0042	0.0011	0.1423
11q24.2	.	.	.	.	.	.	0.0024	0.0084	0
11q24.3	.	.	.	.	.	.	0.0083	0.003	0.1313
11q25	.	.	.	.	.	.	0	0	0
11q25	.	.	.	.	.	.	0.0015	0.0011	0.0346
12p13.33	.	Benign|Likel	not_specifie	RCV0001764	MedGen|Me	CN169374|C	0.0018	0.0007	0.0525
12p13.31	.	.	.	.	.	.	0	0	0
12q13.12	.	.	.	.	.	.	0	0	0
12q13.2	.	.	.	.	.	.	0	0	0
12q13.3	.	.	.	.	.	.	0.0035	0.0011	0.0931
12q13.3	.	Benign|Likel	not_specifie	RCV0000384	MedGen|Me	CN169374|C	0.0067	0.0023	0.1814
12q21.32	.	.	.	.	.	.	0.0005	0.0002	0.0156
12q23.3	.	.	.	.	.	.	0.0034	0.0014	0.043

12q23.3	.	.	.	.	.	.	0.0011	0.0003	0.0107
12q24.13	.	.	.	.	.	.	0.0049	0.0025	0.1384
13q13.1	.	.	.	.	.	.	0	0	0
13q34	.	.	.	.	.	.	0.00006462	0	0
14q11.2	.	.	.	.	.	.	0	0	0
14q22.3	.	.	.	.	.	.	0.0002	0	0.0024
14q23.1	.	.	.	.	.	.	0.0004	0	0
14q24.1	.	.	.	.	.	.	0	0	0
14q32.11	.	.	.	.	.	.	0	0	0
14q32.12	.	.	.	.	.	.	0.0008	0.0027	0
14q32.2	.	.	.	.	.	.	0.0044	0.0011	0.1253
15q15.2	.	.	.	.	.	.	0.0043	0.0029	0.1038
15q15.2	.	.	.	.	.	.	0.00003235	0	0
15q15.3	.	.	.	.	.	.	0	0	0
15q21.1	.	.	.	.	.	.	0.0005	0.0002	0.0155
15q22.1	.	.	.	.	.	.	0	0	0
15q23	.	.	.	.	.	.	0.0085	0.022	0.0752
15q25.3	.	.	.	.	.	.	0.0042	0.0015	0.1091
15q25.3	.	.	.	.	.	.	0.00009697	0	0
15q25.3	.	.	.	.	.	.	0.0032	0.0013	0.0742
16p13.3	.	.	.	.	.	.	0.00006462	0	0
16p13.3	.	.	.	.	.	.	0.0053	0.0182	0.0024
16p13.3	.	.	.	.	.	.	0	0	0
16p13.3	.	.	.	.	.	.	0.0000323	0.0001	0
16p12.3	.	.	.	.	.	.	0	0	0
16p12.3	.	.	.	.	.	.	0.0071	0.0003	0.0263
16p12.1	.	.	.	.	.	.	0.0005	0.002	0
16p11.2	.	.	.	.	.	.	0.0026	0.009	0
16p11.2	.	.	.	.	.	.	0.0069	0.024	0.0024
16p11.2	.	.	.	.	.	.	0	0	0
16p11.2	.	.	.	.	.	.	0.0071	0.0247	0.0024
16p11.2	.	.	.	.	.	.	0	0	0
16q21	.	.	.	.	.	.	0	0	0

16q21	.	.	.	.	.	.	0.0029	0.0102	0.0012
16q22.3	.	.	.	.	.	.	0.0011	0.0005	0.0322
16q23.3	.	.	.	.	.	.	0.0062	0.0077	0.0084
17p13.3	.	.	.	.	.	.	0.0007	0.0003	0.0131
17p13.2	.	.	.	.	.	.	0.0096	0.0038	0.2053
17p13.1	.	Benign	not_specifie	RCV0002032	MedGen	CN169374	0.0038	0.0022	0.105
17p13.1	.	.	.	.	.	.	0	0	0
17p11.2	.	.	.	.	.	.	0	0	0
17q12	.	.	.	.	.	.	0.0008	0.0003	0
17q21.2	.	.	.	.	.	.	0.0023	0.0001	0.006
17q21.31	.	.	.	.	.	.	0	0	0
17q21.33	.	.	.	.	.	.	0	0	0
17q21.33	.	.	.	.	.	.	0.001	0.0008	0.0239
17q23.3	.	.	.	.	.	.	0.0035	0.0016	0.1026
17q25.1	.	.	.	.	.	.	0.00003249	0	0
17q25.1	.	Likely benign	Epidermolysi	RCV0003061	MedGen:OM	C1856934:22	0.0017	0.001	0.0382
17q25.1	.	.	.	.	.	.	0	0	0
18p11.31	.	Benign|Beni	not_specifie	RCV0002179	MedGen|Hu	CN169374|H	0.0028	0.0007	0.0489
18p11.31	.	.	.	.	.	.	0	0	0
18p11.21	.	.	.	.	.	.	0.0021	0.0072	0.0012
18p11.21	.	.	.	.	.	.	0.0026	0.0092	0.0012
18q11.2	.	.	.	.	.	.	0	0	0
18q12.1	.	.	.	.	.	.	0.0061	0.0217	0.0012
18q21.1	.	.	.	.	.	.	0.0001	0	0
18q21.2	.	.	.	.	.	.	0.0004	0.0011	0
19p13.3	.	.	.	.	.	.	0.0011	0.0011	0.028
19p13.2	.	.	.	.	.	.	0.0037	0.0005	0.0179
19p13.2	.	.	.	.	.	.	0.0017	0.0011	0.0453
19p13.2	.	.	.	.	.	.	0	0	0
19p13.2	.	.	.	.	.	.	0.0012	0.0001	0.0418
19p13.2	.	.	.	.	.	.	0	0	0
19p13.11	.	.	.	.	.	.	0	0	0
19q13.11	.	.	.	.	.	.	0	0	0

19q13.2	.	.	.	.	.	.	0.0076	0.0039	0.1337
19q13.2	.	.	.	.	.	.	0.0091	0.0011	0.068
19q13.32	.	.	.	.	.	.	0.0045	0.0012	0.1308
19q13.33	.	.	.	.	.	.	0	0	0
19q13.41	.	.	.	.	.	.	0.0057	0.0039	0.0112
19q13.41	.	.	.	.	.	.	0.0041	0.0017	0.0538
19q13.42	.	.	.	.	.	.	0.0083	0.002	0.1679
19q13.43	.	.	.	.	.	.	0	0	0
20p11.21	.	.	.	.	.	.	0	0	0
20q11.23	.	.	.	.	.	.	0.00003229	0	0.0012
20q11.23	.	.	.	.	.	.	0	0	0
20q12	.	Benign	not_specifie	RCV0002029	MedGen	CN169374	0.0099	0.0023	0.0704
20q13.12	.	.	.	.	.	.	0.0042	0.0002	0.0847
20q13.31	.	.	.	.	.	.	0	0	0
20q13.33	.	.	.	.	.	.	0.0011	0.0005	0.0191
20q13.33	.	.	.	.	.	.	0.002	0.0002	0.0668
21q22.3	.	.	.	.	.	.	0	0	0
22q11.23	.	.	.	.	.	.	0.0034	0.001	0.0979
22q12.2	.	.	.	.	.	.	0.0025	0.0086	0
22q12.2	.	.	.	.	.	.	0.0002	0.0006	0
22q12.2	.	.	.	.	.	.	0.0002	0.0006	0
22q12.2	.	.	.	.	.	.	0	0	0
22q12.2	.	.	.	.	.	.	0.0083	0.003	0.0871
22q13.1	.	.	.	.	.	.	0.0004	0.0003	0.0036
22q13.2	.	.	.	.	.	.	0.0034	0.001	0.0943
22q13.2	.	.	.	.	.	.	0	0	0
22q13.33	.	.	.	.	.	.	0	0	0
Xp22.33	.	.	.	.	.	.	0.0004	0.0005	0.0024
Xp22.33	.	.	.	.	.	.	0.0006	0.0008	0.0024
Xp22.33	.	.	.	.	.	.	0.0006	0.0008	0.0024

gnomAD_ger	gnomAD_ger	gnomAD_ger	gnomAD_ger	gnomAD_ger	3.5jpn_v2	1000g2015au	1000g2015au	ExAC_ALL	ExAC_AFR
0.0166	0.0043	0.0006	0.0069	0.0061	0.0176	0.0109824	0.0109	0.0173	0.0029
0	0.0068	0	0.0001	0	0	0.00319489	0	0.0025	0.0005
0.0172	0	0.0007	0.002	0.0045	0	0	0	0.0063	0.0544
0	0	0	0.00008095	0	0	0	0	0.001	0
0.0066	0.024	0.0006	0.0033	0.0102	0.0277	0.0305511	0.003	0.0228	0.0047
0	0	0	0	0.0051	0	0.00878594	0	0.0086	0.001
0	0	0	0.0013	0.0092	0	0.0253594	0.002	0.0252	0.0021
0	0.0025	0.0003	0.0003	0.0143	0.0412	0.028155	0.001	0.0193	0.0027
0	0	0	0.00006668	0	0	0.00059904	0.002	0.0004	0
0	0	0	0.00006662	0.0112	0	0.0209665	0	0.0228	0.0034
0	0	0	0	0	0	0	0	0	0
0	0	0	0.00006661	0	0	0.00019968	0	0.0001	0.0002
0	0	0	0	0	0	0	0	0.00002491	0.0003
0.0132	0.0649	0	0.0005	0.0071	0.0709	0.0249601	0	0.016	0.0022
0	0	0	0.0004	0.0082	0	0.0119808	0.002	0.0062	0.0015
0	0	0	0	0	0	0	0	0	0
0	0	0	0	0	0	0.00079872	0	0.0005	0.0061
0	0	0	0	0.001	0	0.00579073	0	0.0015	0.0165
0	0	0	0.0002	0.0051	0	0.0147764	0	0.0125	0.0013
0	0	0	0.00006668	0.002	0	0.0081869	0	0.0028	0.0341
0	0	0	0	0	0	0	0	0	0
0	0	0	0	0	0	0.00019968	0	8.295E-06	0
0	0	0	0	0	0	0	0	0	0
0	0.0241	0.0034	0.0025	0.0041	0.0149	0.0171725	0.002	0.0149	0.0025
0	0	0	0.00006675	0.001	0	0.00379393	0	0.0005	0
0	0	0	0	0	0	0	0	0	0
0	0.0715	0.0023	0.0003	0.0122	0.0676	0.0485224	0	0.026	0.0026
0	0	0	0	0	0	0	0	8.346E-06	0
0	0	0	0.00006664	0	0	0.00798722	0	0.0017	0.0182
0.0036	0.002	0.0009	0.0006	0	0	0	0	0.2545	0.442
0	0	0	0	0	0	0	0	8.738E-06	0
0	0.0111	0.0003	0.0005	0.0041	0.0134	0.00459265	0	0.0015	0

0	0	0	0	0	0	0	0	0.00005793	0
0	0	0	0	0	0	0.00479233	0	0.0054	0.0002
0	0	0	0	0	0	0	0	8.419E-06	0
0	0	0	0	0	0	0	0	0	0
0	0	0	0	0.002	0	0.00579073	0	0.0056	0.0015
0	0	0	0	0	0	0.00019968	0.001	0.00001659	0
0	0.0624	0	0.0004	0	0.0152	0.0111821	0.002	0.0049	0.0003
0	0.0878	0.01	0.0009	0.0061	0.0486	0.0201677	0.001	0.01	0.0004
0.0033	0.0019	0.0037	0.0004	0.0051	0.0318	0.00858626	0.001	0.0079	0.0019
0	0.0006	0	0.00006665	0.0041	0	0.00239617	0	0.0045	0.0008
0	0	0	0	0	0	0	0	0	0
0	0	0	0	0	0.0563	0	0	0.098	0.0648
0	0.0006	0	0.0003	0.0031	0	0.0161741	0	0.021	0.0029
0	0	0	0.00006662	0	0	0.00019968	0.001	0.00004955	0
0	0	0	0	0	0	0	0	0	0
0	0.0117	0.0049	0.0014	0.0071	0.0443	0.0131789	0.002	0.0066	0.0005
0	0.0006	0	0	0	0	0.0101837	0	0.0033	0.0006
0.0033	0.0012	0.0052	0.0041	0.0061	0	0.014377	0.004	0.0151	0.0015
0.0033	0.0173	0.0123	0.0024	0.0112	0	0.014976	0.008	0.0113	0.0009
0	0	0	0	0	0	0	0	0.00004978	0
0	0	0	0	0	0	0	0	0	0
0	0	0	0	0	0	0	0	0	0
0.0033	0.0518	0	0.0002	0.0061	0.0519	0.019369	0	0.0173	0.0014
0	0	0.0043	0.0079	0.0041	0	0.00259585	0.0089	0.0097	0.0024
0.0033	0.0006	0.0103	0.0055	0.0081	0	0.0165735	0.002	0.023	0.0042
0	0	0	0	0	0	0	0	0	0
0	0	0.0126	0.0005	0.0051	0	0.00379393	0.001	0.0032	0.0008
0	0	0	0	0	0	0	0	0	0
0	0	0	0	0	0	0	0	0	0
0	0.0012	0.0003	0.0025	0.0092	0	0.0309505	0.003	0.0318	0.0035
0.0033	0.058	0.0003	0.0015	0.0081	0.0518	0.0283546	0.001	0.0208	0.0013
0	0	0	0	0	0	0	0	0.0002	0.0002
0	0	0	0	0	0	0	0	0.00001011	0

0	0	0	0	0	0	0.00738818	0	0.0022	0.0258
0	0	0.0003	0.0005	0	0	0.00559105	0.001	0.0024	0.0222
0	0	0	0	0	0	0.0081869	0	0.0053	0.0397
0	0	0	0	0	0	0.00039936	0	0.0007	0.00009844
0	0	0	0	0	0	0	0	0	0
0	0	0	0	0	0	0	0	0	0
0	0	0	0.0003	0.0031	0	0.00698882	0	0.0023	0.0246
0	0.0444	0	0.00006661	0.0112	0.0139	0.0347444	0	0.0221	0.0031
0	0	0.0038	0.0011	0.0041	0	0.00159744	0.004	0.0007	0
0	0	0	0	0	0	0	0	0	0
0	0.0043	0.0009	0.0003	0	0.0124	0.00738818	0.001	0.0046	0.0014
0	0.0012	0	0.00006667	0.0031	0	0.00419329	0	0.0053	0.0003
0	0.016	0	0.00006682	0.002	0	0.00539137	0	0.0064	0.0013
0	0.0345	0.0029	0.0002	0.002	0.0866	0.00958466	0	0.0065	0.001
0	0	0	0	0	0	0	0	0.00000828	0
0	0	0	0	0	0	0	0	0	0
0	0	0	0	0	0	0	0	8.239E-06	0
0	0	0	0	0	0	0	0	0	0
0	0	0	0	0	0	0.00039936	0	0.0000637	0.0007
0	0	0	0.00006678	0.001	0	0.00379393	0.001	0.0009	0.0097
0.0464	0.0012	0.0034	0.0127	0.0132	0	0.0091853	0.0119	0.0108	0.0035
0	0.0019	0	0.0003	0.0031	0	0.00938498	0	0.0059	0.0008
0.0033	0.0136	0.0009	0.0013	0.0051	0.0331	0.0147764	0.001	0.0127	0.0008
0	0.0377	0.0052	0.0009	0.0164	0.0843	0.0247604	0.001	0.0255	0.0013
0	0	0	0	0	0	0.00039936	0	0.00004336	0.0001
0	0	0	0	0	0	0	0	8.237E-06	0
0	0.0006	0	0.0003	0.0051	0	0.00579073	0	0.006	0.0016
0	0	0	0	0	0	0	0	0	0
0	0.0234	0	0.0003	0.0071	0.0995	0.0215655	0.002	0.0141	0.002
0	0	0	0	0.0031	0	0.00858626	0	0.0083	0.0013
0	0.0006	0	0.0002	0.001	0	0.0111821	0	0.0042	0.0425
0	0	0	0.00006664	0.002	0	0.00239617	0	0.0009	0.0098
0	0	0	0.00006661	0.002	0	0.00359425	0	0.0025	0.0001

0	0	0	0.0003	0	0.0114	0.00259585	0	0.0027	0.0006
0	0	0	0	0	0	0	0	0	0
0	0	0.0189	0.0018	0.0122	0	0.0061901	0.004	0.0078	0.0013
0.0033	0	0	0.0011	0.0031	0	0.0129792	0.001	0.0075	0.0302
0	0.0117	0.0077	0.0005	0.0051	0.0251	0.0091853	0.002	0.0084	0.0009
0	0.0055	0.0132	0.0009	0.002	0.021	0.00279553	0	0.0024	0.0005
0.0033	0.0006	0	0	0	0	0.00159744	0	0.0026	0.0004
0	0	0	0.00006664	0.0051	0	0.0129792	0	0.0015	0.0005
0	0	0	0	0.001	0	0.00039936	0	0.002	0
0	0	0	0.00006672	0	0	0.00199681	0	0.0006	0.0062
0	0	0	0.0001	0	0	0	0	0.0000172	0
0	0.0019	0	0	0	0	0	0	0.00002486	0
0	0.0277	0.004	0.0003	0.0031	0.032	0.0117812	0.001	0.0083	0.001
0	0	0	0.0002	0.0062	0	0.00838658	0	0.0085	0.0017
0.0033	0.1019	0	0.0005	0.0092	0.0785	0.0305511	0	0.0178	0.0014
0	0	0	0	0	0	0	0	0	0
0	0.0241	0	0.0002	0.0031	0	0.0155751	0	0.0102	0.0012
0	0	0	0	0	0	0	0	0.00001647	0
0	0	0.0003	0.0004	0.0071	0	0.0169728	0	0.0147	0.0019
0	0	0	0.0013	0.001	0	0.00019968	0.001	0.0008	0.0002
0	0	0	0	0	0	0	0	0	0
0	0	0	0.0001	0.0031	0	0.0131789	0.001	0.0043	0.0047
0	0	0	0	0	0	0.00019968	0	0.0006	0.0002
0	0	0	0	0	0	0	0	0.0000092	0
0	0	0	0	0	0.014	0	0	0	0
0	0.012	0	0	0	0	0	0	0.0065	0.0282
0.0033	0	0	0.0003	0	0	0.00019968	0	0.0003	0
0	0.0179	0	0.00006674	0.002	0.0203	0.0241613	0	0.0159	0.0028
0	0	0	0.00006696	0	0	0.00019968	0	0	0
0.0066	0	0	0.00006676	0	0	0	0	0.0002	0
0	0.0037	0	0	0	0	0.00399361	0	0.0013	0.0115
0.0066	0.1485	0.0037	0.0011	0.0031	0.1492	0.0325479	0	0.0161	0.0004
0	0	0	0	0	0	0	0	8.238E-06	0

0	0.0006	0	0	0.0081	0	0.0219649	0	0.0167	0.0018
0	0.0012	0	0.00006662	0.002	0	0.00379393	0	0.0023	0.0006
0	0	0	0	0	0	0	0	0.00002471	0
0	0	0	0	0	0	0	0	0	0
0	0	0	0	0	0	0.00059904	0	0.0015	0
0	0	0	0	0	0	0	0	0	0
0	0	0	0.0001	0	0	0.00019968	0.001	0.00007416	0
0	0	0	0	0	0	0	0	0	0
0	0	0	0	0	0	0	0	0	0
0	0	0	0	0	0	0	0	0	0
0	0	0	0	0	0	0	0	0	0
0	0	0	0	0	0	0.00738818	0	0.0072	0.0009
0.0033	0.0025	0.024	0.0015	0.0132	0	0.0115815	0.003	0.0158	0.0011
0	0	0	0.0008	0.0123	0	0.0145767	0	0.0185	0.0026
0	0.0006	0.0026	0.006	0.0031	0	0.00279553	0.004	0.0044	0.0013
0	0.0438	0	0.0006	0.002	0.0724	0.0165735	0	0.0118	0.0011
0	0	0.0372	0.0079	0.0163	0	0.00259585	0.008	0.0081	0.0027
0	0	0.0026	0.0048	0.002	0	0.00059904	0.001	0.0032	0.0011
0	0	0	0	0	0	0	0	0.00002476	0
0	0	0	0	0	0	0	0	0	0
0	0.0043	0.0016	0.0008	0.0095	0	0.0101837	0.001	0.0093	0
0	0	0	0.00006668	0	0	0.00359425	0.001	0.0009	0.0083
0	0.0006	0.0014	0.0071	0.0081	0	0.0209665	0.0099	0.0204	0.003
0	0	0	0	0	0	0	0	0	0
0	0.0012	0	0.0001	0.002	0	0.00599042	0	0.0053	0.0009
0	0	0	0.0003	0.001	0	0.00898562	0	0.0064	0.0003
0	0	0	0	0	0	0.00019968	0	0.00003076	0
0	0	0	0	0	0	0	0	0	0
0	0	0	0	0	0	0	0	0	0
0	0.008	0.0003	0.0002	0.0041	0	0.0157748	0	0.0126	0.0006
0	0.0136	0	0.0003	0.0102	0	0.0225639	0.001	0.0246	0.0021
0	0	0	0	0.001	0	0.00319489	0	0.0021	0.0007
0	0	0.0023	0.0031	0.0031	0	0.0061901	0.003	0.006	0.0018

0	0.0012	0.0037	0.0005	0	0	0.0009984	0	0.0018	0.0004
0	0	0.0006	0.0001	0.0112	0	0.0235623	0.001	0.0187	0.0021
0	0	0	0	0	0	0	0	0	0
0	0	0	0.00006671	0.001	0	0	0	0.00005877	0.00009872
0	0	0	0	0	0	0	0	0	0
0	0	0	0.0001	0.001	0	0	0	0.0003	0.0001
0	0.0062	0	0.0001	0	0	0.00499201	0	0.0036	0.0002
0	0	0	0	0	0	0	0	0	0
0	0	0	0	0	0	0	0	0.00001681	0
0	0	0	0	0	0	0.00059904	0	0.0002	0.0023
0	0.0068	0	0.00006663	0.0081	0	0.0209665	0	0.0145	0.0013
0	0.0074	0.0003	0.0002	0.0041	0.0117	0.014377	0.001	0.0018	0.0031
0	0	0	0.00006681	0	0	0	0	0	0
0	0	0	0	0	0	0	0	0	0
0	0	0	0.00006676	0	0	0.00159744	0	0.0014	0.0004
0	0	0	0	0	0	0	0	0	0
0	0.0025	0	0.0001	0.0031	0	0.0177716	0	0.01	0.023
0	0.0148	0	0.00006695	0.0031	0.0179	0.0205671	0	0.0566	0.0043
0	0	0	0.0002	0	0	0	0	0.0001	0
0	0.0112	0	0.0003	0.002	0.0177	0.0113818	0	0.012	0.0023
0	0	0	0.0001	0	0	0	0	0.00008435	0
0	0	0	0.00006674	0.002	0	0.0081869	0	0.0023	0.0299
0	0	0	0	0	0	0	0	0.00001281	0
0	0	0	0	0	0	0	0	0.00004209	0.0002
0	0	0	0	0	0	0	0	0.00001656	0
0	0.0068	0.0372	0.0025	0.0184	0.0218	0.0107827	0.006	0.0059	0.0002
0	0	0	0	0	0	0.00079872	0	0.0001	0.0016
0	0	0	0	0.001	0	0.00239617	0	0.0025	0.0105
0	0	0	0.0001	0	0	0.00838658	0	0.0023	0.0263
0	0	0	0	0	0	0.00019968	0	0	0
0	0	0	0.00006672	0.001	0	0.00658946	0	0.002	0.0222
0	0	0	0	0	0	0	0	0.00002477	0
0	0	0	0	0	0	0	0	0	0

0	0	0	0.00006664	0	0	0.00319489	0	0.0009	0.0107
0	0	0	0.00006667	0.002	0	0.00738818	0	0.0054	0.0003
0.0265	0	0.0014	0.0062	0.0112	0	0.00559105	0.0139	0.0066	0.0093
0.0066	0.0031	0	0.00006668	0.001	0	0.00499201	0.001	0.0026	0.0007
0.0033	0.0006	0.0003	0.0051	0.0112	0	0.0229633	0.005	0.0286	0.0041
0	0	0	0.0003	0.0062	0	0.0161741	0	0.0162	0.0024
0	0	0	0	0	0	0	0	0	0
0	0	0	0	0	0	0	0	0	0
0	0	0.0009	0.0013	0	0	0.00079872	0.003	0.0016	0.0002
0	0.0389	0	0.00006662	0.002	0.0267	0.0081869	0	0.0029	0.00009612
0	0	0	0	0	0	0	0	0	0
0	0	0	0	0	0	0	0	8.246E-06	0.00009619
0	0	0	0.0003	0.001	0	0.00539137	0	0.0045	0.0006
0	0	0	0.00006668	0.0061	0	0.0127796	0	0.0137	0.0011
0	0.0006	0	0	0	0	0.00019968	0	0.00005756	0
0	0.0031	0	0.0001	0.0031	0	0.0061901	0	0.0057	0.0022
0	0	0	0	0	0	0	0	0	0
0	0.0185	0	0.0004	0.0041	0	0.0105831	0	0.0086	0.001
0	0	0	0	0	0	0	0	0	0
0	0	0	0	0	0	0.00239617	0	0.0007	0.0073
0	0	0	0	0	0	0.00259585	0	0.0009	0.0095
0	0	0	0	0	0	0.00019968	0	0.00002474	0
0	0	0	0	0	0	0.00519169	0	0.0019	0.0211
0	0.0019	0	0	0	0	0	0	0	0
0	0	0	0.00006661	0	0	0.0009984	0	0.0001	0.0011
0	0	0	0	0.0033	0	0.00439297	0	0	0
0	0.0549	0	0.0003	0.0041	0.0515	0.0125799	0	0.0076	0.0006
0	0	0	0	0.0041	0	0.0081869	0	0.0034	0.0007
0	0	0	0	0	0	0.0213658	0.0368	0	0
0	0	0	0.00006662	0	0	0.00579073	0	0.0022	0.0011
0	0	0	0	0	0	0	0	0	0
0	0	0	0	0	0	0	0	0.0000614	0.0001
0	0	0	0	0	0	0	0	0	0

0.0167	0.0253	0.0003	0.0024	0.0071	0.0619	0.0245607	0.001	0.0173	0.0037
0.0066	0	0.0263	0.0073	0.0102	0	0.0175719	0.0089	0.0156	0.0016
0	0.0037	0	0.0001	0.0096	0	0.0151757	0	0.0219	0.002
0	0	0	0	0	0	0.00039936	0	0	0
0.0035	0.0067	0.0026	0.0069	0.0083	0	0	0	0.0001	0.0003
0	0	0	0.0041	0.0061	0	0.0071885	0.001	0.0082	0.0021
0	0.0302	0.0029	0.0022	0.0112	0.0211	0.0189696	0	0.0028	0.0039
0	0	0	0	0	0	0	0	0	0
0	0	0	0	0	0	0	0	0.00000927	0
0	0	0	0	0	0	0.00019968	0	0.00008544	0
0	0	0	0	0	0	0	0	8.419E-06	0
0.0166	0.0019	0.0135	0.0106	0.0143	0	0.0119808	0.005	0.0144	0.0021
0	0.0259	0	0.0007	0.0051	0.0204	0.0139776	0	0.0136	0.0007
0	0	0	0	0	0	0	0	0	0
0	0	0.0003	0.0006	0.0031	0	0.00339457	0	0.0046	0.0005
0	0	0	0	0.0051	0	0.0127796	0	0.0132	0.0004
0	0	0	0	0	0	0	0	0.00009055	0.0001
0.0099	0	0	0.0003	0.0072	0	0.0185703	0	0.0128	0.0016
0	0	0	0.0001	0	0	0.00239617	0	0.0008	0.0086
0	0	0	0	0	0	0	0	0.00002519	0.0002
0	0	0	0	0	0	0	0	8.237E-06	0.0000961
0	0	0	0	0	0	0	0	0	0
0	0.0728	0.0029	0.0016	0.0051	0.0553	0.0249601	0.001	0.0252	0.0033
0	0.0006	0	0.0003	0	0	0.00019968	0	0.0007	0.0006
0	0.0031	0	0.0003	0.0082	0	0.0159744	0.001	0.0093	0.0011
0	0	0	0	0	0	0	0	0	0
0	0	0	0	0	0	0	0	0.00002699	0
0	0	0	0.0003	0	0	0	0	0.1017	0.0658
0	0	0.0011	0.0003	0	0	0	0	0.0438	0.03
0	0	0.0011	0.0003	0	0	0	0	0.0427	0.0298

ExAC_AMR	ExAC_EAS	ExAC_FIN	ExAC_NFE	ExAC_OTH	ExAC_SAS	avsnp147	SIFT_score	SIFT_convert	SIFT_pred
0.092	0.012	0	0.0121	0.0136	0.0082	rs145087137	0.014	0.531	D
0.0163	0.0071	0.0003	0.00007517	0.0011	0.0026	rs150612979	0	0.912	D
0.0012	0.0012	0	0.0089	0.0172	0	rs6667693	0	0.912	D
0.0007	0	0	0.0051	0	0.0007	rs61775089	0	0.912	D
0.1931	0.026	0.0006	0.0032	0.0209	0.0013	rs77928789	0	0.912	D
0.0862	0.0001	0	0.00004642	0.0047	0	rs150246438	0.019	0.501	D
0.2353	0.0014	0.0006	0.001	0.0132	0.0001	rs148608573	0.004	0.682	D
0.1881	0.0096	0.0006	0.0005	0.0187	0.0005	rs12407929	0	0.912	D
0.0032	0	0	0.0002	0.0011	0	rs138546115	0.05	0.395	D
0.2318	0	0	0.0004	0.0045	0	rs142980721	0.036	0.433	D
0	0	0	0	0	0	.	0.001	0.784	D
0.0008	0	0	0.00004495	0.0011	0	rs373262677	0.046	0.405	D
0	0	0	0	0	0	rs113327860	0.013	0.538	D
0.115	0.0576	0	0.0009	0.0088	0.0014	rs3814299	0.002	0.721	D
0.1648	0	0	0.0004	0.0111	0.0008	rs149718823	0.034	0.44	D
0	0	0	0	0	0	.	0.001	0.784	D
0	0	0	0	0	0	rs149267462	0.004	0.721	D
0.0004	0	0	0.00007498	0	0.0000606	rs151330882	0.005	0.632	D
0.1294	0	0	0.0002	0	0	rs998688	0.026	0.469	D
0.0012	0	0	0	0.0026	0	rs114840329	0.008	0.586	D
0	0	0	0	0	0	.	0.047	0.403	D
0	0	0	0	0	0.00006061	rs532010657	0.024	0.477	D
0	0	0	0	0	0	.	0.002	0.721	D
0.1227	0.0172	0.0056	0.0013	0.0124	0.0015	rs139614117	0.001	0.784	D
0.0246	0	0	0	0.0041	0	rs142356826	0	0.912	D
0	0	0	0	0	0	.	0.012	0.555	D
0.1192	0.0748	0.0014	0.0004	0.0155	0.0628	rs3754413	0.016	0.518	D
0.00008645	0	0	0	0	0	rs762104527	0.01	0.564	D
0.0007	0	0	0.00004503	0.0022	0	rs61742803	0	0.912	D
0.2873	0.3726	0.256	0.2061	0.2325	0.2445	rs73146195	0.025	0.473	D
0	0	0	0.00001591	0	0	rs773540330	0.003	0.682	D
0.0171	0.004	0.0016	0.0004	0.0091	0.0025	rs191820463	0.042	0.416	D

0	0	0	0.0002	0	0	rs74318890	0.045	0.408	D
0.0554	0	0	0.00007492	0.0033	0	rs141666493	0.002	0.721	D
0.00008818	0	0	0	0	0	rs746642455	0.002	0.721	D
0	0	0	0	0	0	.	0	0.912	D
0.0554	0	0	0.0002	0.0011	0.0001	rs190983114	0.005	0.912	D
0	0	0	0.00003003	0	0	rs538625524	0.001	0.784	D
0.0111	0.0468	0	0.0003	0.0033	0.0019	rs3732305	0.003	0.682	D
0.0178	0.092	0.0083	0.0013	0.0109	0.0029	rs76767949	0.017	0.512	D
0.1055	0.0248	0.0052	0.0018	0.0174	0.0007	rs143491867	0	0.912	D
0.0459	0	0	0.00009006	0.0011	0	rs143191074	0.007	0.599	D
0	0	0	0	0	0	.	0.037	0.614	D
0.0911	0.1311	0.1118	0.1027	0.0905	0.0833	rs75862065	0.008	0.586	D
0.2158	0	0	0.0004	0.0084	0.00007732	rs12471771	0.001	0.784	D
0	0	0	0.00009007	0	0	rs374549843	0	0.912	D
0	0	0	0	0	0	.	0.01	0.564	D
0.0425	0.0197	0.0071	0.001	0.0078	0.0008	rs148270536	0	0.912	D
0.0333	0.0005	0	0.00004495	0	0	rs61740605	0.001	0.784	D
0.1162	0.0002	0.0056	0.005	0.0088	0.0055	rs142503044	0.033	0.443	D
0.0594	0.0172	0.0126	0.0024	0.0167	0.0163	rs148474013	0	0.912	D
0.0005	0	0	0	0	0	rs755639567	0	0.912	D
0	0	0	0	0	0	.	0.002	0.721	D
0	0	0	0	0	0	.	0.003	0.682	D
0.1354	0.0522	0.0002	0.0004	0.0189	0.0008	rs74823804	0	0.912	D
0.0421	0	0.0063	0.0133	0.0055	0.001	rs28763904	0.034	0.912	D
0.1759	0.001	0.0088	0.0075	0.0212	0.0068	rs4857276	0.006	0.721	D
0	0	0	0	0	0	.	0.016	0.518	D
0.0194	0.0016	0.0103	0.0008	0.0045	0.0005	rs201533072	0.002	0.721	D
0	0	0	0	0	0	.	0.003	0.721	D
0	0	0	0	0	0	.	0.001	0.784	D
0.3103	0.0005	0.0003	0.0028	0.0145	0.0005	rs139285983	0	0.912	D
0.0509	0.0581	0.0005	0.0024	0.0198	0.0754	rs2276790	0.006	0.614	D
0.0014	0	0	0	0.0011	0.0003	rs753827309	0.015	0.524	D
0	0.0001	0	0	0	0	rs74377230	0	0.912	D

0.0009	0	0	0.00003189	0	0	rs114467899	0.007	0.614	D
0.0028	0	0	0.001	0.0015	0.00007097	rs149905649	0.001	0.784	D
0.0032	0	0	0	0	0.0001	rs148017639	0.05	0.395	D
0.0044	0.0002	0	0.0002	0	0.0005	rs372911936	0.001	0.784	D
0	0	0	0	0	0	.	0.012	0.546	D
0	0	0	0	0	0	.	0.006	0.912	D
0.0016	0	0	0.0001	0.0034	0.0001	rs28485258	0.003	0.682	D
0.1894	0.0428	0	0.0003	0.0078	0.0018	rs4365796	0.008	0.586	D
0	0	0	0.0004	0.0085	0.0008	rs535155432	0.034	0.44	D
0	0	0	0	0	0	.	0.001	0.784	D
0.0417	0.006	0.0005	0.00007499	0.0022	0.0002	rs139754344	0.003	0.682	D
0.0539	0.0013	0	0.00006015	0.0033	0	rs190203684	0.001	0.784	D
0.1243	0.0177	0	0.0005	0.0042	0	rs5744539	0.028	0.461	D
0.0299	0.0434	0.0014	0.0004	0.0044	0.0012	rs17844350	0.008	0.586	D
0.0000864	0	0	0	0	0	rs756115221	0	0.912	D
0	0	0	0	0	0	.	0.016	0.531	D
0	0	0	0.00001499	0	0	rs765634337	0	0.912	D
0	0	0	0	0	0	.	0.002	0.721	D
0	0	0	0	0	0	rs202006112	0.041	0.419	D
0.0008	0	0	0.00008326	0.0013	0	rs201036666	0.012	0.546	D
0.0113	0.0017	0.0032	0.0134	0.011	0.0122	rs142803339	0.008	0.784	D
0.0563	0.005	0	0.00007498	0.0055	0.0002	rs61737340	0.028	0.461	D
0.0986	0.0174	0	0.0025	0.0023	0.002	rs117171552	0	0.912	D
0.1627	0.052	0.0062	0.0013	0.02	0.0351	rs17194565	0.002	0.721	D
0.0002	0.0001	0	0	0	0.00006196	rs555534146	0	0.912	D
0.0000864	0	0	0	0	0	rs201161706	0	0.912	D
0.0589	0.0001	0	0.00007902	0.0012	0	rs140530599	0.023	0.481	D
0	0	0	0	0	0	.	0.048	0.4	D
0.1145	0.0347	0	0.0007	0.0111	0.0002	rs74466886	0.032	0.461	D
0.0827	0	0	0.0001	0.0011	0.00007056	rs147880570	0.006	0.614	D
0.0024	0.0004	0	0.0004	0.0014	0	rs61745221	0	0.912	D
0.0002	0.0002	0	0.00002406	0	0.0002	rs145239537	0.018	0.506	D
0.0261	0	0	0.0000456	0.0011	0	rs186372390	0.018	0.506	D

0.0239	0.0027	0.0002	0.0003	0.0033	0.0004	rs150722853	0	0.912	D
0	0	0	0	0	0	.	0.019	0.501	D
0.0484	0.0006	0.0165	0.003	0.0089	0.0033	rs145989307	0.031	0.45	D
0.0376	0.0001	0	0.0016	0.0044	0.0027	rs115450325	0.008	0.586	D
0.0574	0.0179	0.0135	0.0013	0.0056	0.0002	rs140475659	0	0.912	D
0.012	0.005	0.0083	0.0006	0.0025	0.0002	rs144237288	0	0.912	D
0.025	0.0017	0	0.00002997	0.0022	0.00006056	rs2302824	0.021	0.491	D
0.0732	0	0	0	0.0083	0	rs186048202	0	0.912	D
0.0219	0	0	0	0	0	rs201535252	0.026	0.477	D
0.0005	0.0002	0	0.00007678	0	0.00006061	rs149999873	0.048	0.4	D
0	0	0	0.00003133	0	0	rs372108562	0	0.912	D
0	0	0	0.00004514	0	0	rs201351339	0	0.912	D
0.0416	0.0397	0.0025	0.0014	0.0056	0.0013	rs117397164	0.003	0.682	D
0.0867	0	0	0.0001	0.0056	0.0001	rs149352514	0.011	0.555	D
0.0619	0.1063	0	0.0004	0.0245	0.0298	rs74846385	0	0.912	D
0	0	0	0	0	0	.	0	0.912	D
0.0855	0.0228	0	0.00004496	0.0055	0.0019	rs77653001	0.038	0.427	D
0.0002	0	0	0	0	0	rs754886531	0.016	0.518	D
0.1486	0.0016	0.0002	0.0003	0.0077	0.001	rs145027071	0.035	0.491	D
0.0004	0.0001	0	0.0012	0.0022	0.0005	rs141164874	0.005	0.632	D
0	0	0	0	0	0	.	0.008	0.632	D
0.163	0	0	0	0	0	rs146559587	0.013	0.538	D
0.0059	0	0	0	0	0	rs140138868	0.008	0.586	D
0	0	0	0	0	0.00006899	rs758517075	0.021	0.599	D
0	0	0	0	0	0	rs112212538	0	0.912	D
0.0169	0.0333	0	0.0000735	0	0.0021	.	0	0.912	D
0.00008637	0	0	0.0004	0	0.0006	rs201125580	0.001	0.784	D
0.1317	0.0217	0	0.0001	0.0068	0.0098	rs117033551	0.002	0.721	D
0	0	0	0	0	0	rs368748227	0.008	0.586	D
0.0003	0	0	0.0003	0	0	rs149340579	0	0.912	D
0.0005	0.0033	0	0.00004592	0	0.00006083	rs141298814	0.003	0.682	D
0.0475	0.1394	0.0032	0.0015	0.0156	0.0033	rs2296466	0.019	0.501	D
0	0	0	0.00001499	0	0	rs774121997	0.012	0.546	D

0.1693	0.0002	0	0.0002	0.0111	0	rs142326775	0.003	0.682	D
0.0212	0.0021	0	0.000045	0	0.0002	rs185349094	0.009	0.574	D
0.0002	0	0	0.00001498	0	0	rs770474065	0.001	0.784	D
0	0	0	0	0	0	.	0.012	0.632	D
0.0161	0	0	0	0	0	rs146693057	0.01	0.564	D
0	0	0	0	0	0	.	0.004	0.682	D
0	0.0002	0	0.00005996	0	0.0002	rs75290777	0.015	0.614	D
0	0	0	0	0	0	.	0.003	0.682	D
0	0	0	0	0	0	.	0.018	0.506	D
0	0	0	0	0	0	.	0.012	0.546	D
0	0	0	0	0	0	.	0.026	0.469	D
0.0736	0.0003	0	0.0001	0.0044	0.0002	rs190320444	0	0.912	D
0.14	0.0018	0.0271	0.0012	0.0121	0.0002	rs147713756	0	0.912	D
0.187	0	0	0.0011	0.0011	0.0003	rs143981854	0.03	0.453	D
0.0024	0	0.0017	0.0056	0.0011	0.0066	rs146342750	0.011	0.555	D
0.0794	0.054	0	0.0003	0.0033	0.0007	rs147545257	0.001	0.784	D
0.0135	0.0001	0.0352	0.0072	0.0099	0.0047	rs149901958	0	0.912	D
0.0026	0	0.0014	0.005	0.0022	0.0004	rs147921001	0.034	0.44	D
0	0.0001	0	0.000015	0	0.00006107	rs751861324	0.008	0.586	D
0	0	0	0	0	0	.	0.003	0.682	D
0.25	0	0	0	0	0	rs9332747	0	0.912	D
0.0012	0	0	0.0001	0	0	rs150257705	0.042	0.416	D
0.1545	0.0001	0.0023	0.008	0.0103	0.0034	rs116897071	0	0.912	D
0	0	0	0	0	0	.	0.009	0.599	D
0.0534	0.0003	0	0.0001	0.0044	0.0001	rs138130138	0	0.912	D
0.0638	0.0008	0	0.0002	0.0106	0.0003	rs181994120	0.004	0.654	D
0.0002	0	0	0.00001842	0	0	rs187536858	0	0.912	D
0	0	0	0	0	0	.	0.031	0.45	D
0	0	0	0	0	0	.	0.019	0.506	D
0.1215	0.0086	0.0002	0.0005	0.0055	0.0004	rs2066815	0.001	0.912	D
0.2447	0.0102	0	0.0003	0.0178	0.0002	rs4759043	0.025	0.473	D
0.0212	0	0	0.00004515	0.0022	0	rs146104771	0.019	0.501	D
0.039	0	0.0005	0.0034	0.0099	0.0016	rs150301267	0	0.912	D

0.0137	0.0006	0.0036	0.0002	0.0033	0.0002	rs12423276	0	0.912	D
0.1895	0.0003	0	0.0003	0.0143	0.0008	rs146573098	0.009	0.574	D
0	0	0	0	0	0	.	0.001	0.784	D
0	0	0	0.00009197	0	0	rs377602797	0.004	0.654	D
0	0	0	0	0	0	.	0.001	0.784	D
0.00008738	0.0001	0	0.0005	0	0	rs201423229	0	0.912	D
0	0.0099	0	0.0003	0.0084	0.0138	rs201908822	0.014	0.531	D
0	0	0	0	0	0	.	0.007	0.599	D
0	0	0	0.00003023	0	0	rs372345466	0	0.912	D
0	0	0	0	0	0	rs144321854	0.007	0.599	D
0.1439	0.0075	0	0.0001	0.0121	0	rs74719094	0.002	0.721	D
0.0656	0.009	0	0.0003	0.0089	0.0004	rs117842035	0	0.912	D
0	0	0	0	0	0	.	0.043	0.446	D
0	0	0	0	0	0	.	0	0.912	D
0.0139	0.0001	0	0.0001	0.0012	0.00006351	rs146747045	0.001	0.784	D
0	0	0	0	0	0	.	0	0.912	D
0.0803	0.0007	0	0.0001	0.0033	0.0019	rs138475438	0.042	0.416	D
0.3937	0.0906	0	0.0008	0	0	rs181576780	0.019	0.501	D
0.00008761	0	0	0.0002	0	0	rs143874205	0	0.912	D
0.1127	0.012	0	0.0005	0	0.0011	rs150261781	0.037	0.433	D
0.0004	0	0	0.00007696	0	0	rs201447753	0.029	0.614	D
0	0	0	0.0002	0	0	rs61747753	0.002	0.721	D
0	0	0	0	0	0.00008916	rs553691177	0.039	0.424	D
0	0	0	0.0000153	0	0.0001	rs150558344	0.047	0.403	D
0	0	0	0.00001498	0	0.00006057	rs756437130	0.016	0.912	D
0.0274	0.0064	0.0337	0.0015	0.0143	0	rs138730205	0.006	0.654	D
0.00008682	0	0	0	0	0	rs143331742	0.008	0.614	D
0	0	0	0	0.1	0	rs564246714	0.029	0.457	D
0.0004	0.0004	0	0.00004689	0	0.00006262	rs114581451	0	0.912	D
0	0	0	0	0	0	rs190387347	0.039	0.424	D
0.0013	0	0	0	0.0011	0.00006056	rs75125670	0.001	0.784	D
0	0.0001	0	0.00003004	0	0	rs780288749	0.041	0.421	D
0	0	0	0	0	0	.	0.01	0.564	D

0.0003	0	0	0.00001498	0	0	rs115991261	0.001	0.784	D
0.0504	0	0	0.0001	0.0026	0	rs145239736	0.008	0.586	D
0.0106	0	0.0012	0.008	0.0132	0.0011	rs75472618	0.01	0.564	D
0.0198	0.0022	0	0.0008	0.0011	0.0001	rs142148792	0.009	0.574	D
0.2586	0	0.0003	0.005	0.0212	0.0041	rs146932154	0.003	0.721	D
0.1622	0.0001	0	0.0004	0.0131	0	rs138395797	0.002	0.721	D
0	0	0	0	0	0	.	0.026	0.469	D
0	0	0	0	0	0	.	0.042	0.416	D
0.0004	0	0.0014	0.0016	0.0033	0.0038	rs150508589	0	0.912	D
0.0068	0.0304	0	0.00004495	0.0022	0.0001	rs140876567	0.007	0.599	D
0	0	0	0	0	0	.	0.015	0.524	D
0	0	0	0	0	0	rs755617796	0	0.912	D
0.0452	0	0	0.0001	0.0022	0.0003	rs191998613	0.006	0.654	D
0.1301	0	0	0.00008032	0.0039	0	rs139229826	0	0.912	D
0	0.0005	0	0.00002747	0	0	rs547029082	0.035	0.446	D
0.0524	0.0028	0	0.0002	0.0062	0.0007	rs143203816	0	0.912	D
0	0	0	0	0	0	.	0.002	0.721	D
0.075	0.028	0	0.0002	0.0019	0.0006	rs117342470	0.005	0.632	D
0	0	0	0	0	0	.	0.01	0.564	D
0.0003	0	0	0	0	0	rs61738816	0	0.912	D
0.0005	0	0	0.00001502	0.0011	0	rs146976229	0.048	0.4	D
0	0.0001	0	0.00003	0	0	rs574476197	0	0.912	D
0.0006	0	0	0.00001501	0.0011	0	rs74368609	0.003	0.682	D
0	0	0	0	0	0	.	0	0.912	D
0.0002	0	0	0.00002999	0	0.00006057	rs139976043	0.001	0.784	D
0	0	0	0	0	0	rs575591206	0.003	0.682	D
0.0337	0.0583	0	0.00003005	0.0022	0.0009	rs138148015	0.005	0.632	D
0.1061	0	0	0.0001	0.0051	0	rs189368660	0.002	0.721	D
0	0	0	0	0	0	rs145645281	0.003	0.682	D
0.1053	0.0052	0	0	0	0.0004	rs186754194	0.025	0.473	D
0	0	0	0	0	0	.	0.011	0.555	D
0	0	0	0.00006277	0	0.0001	rs374308521	0.009	0.574	D
0	0	0	0	0	0	.	0.002	0.721	D

0.1376	0.0297	0.0002	0.0023	0.0111	0.0025	rs35762773	0.001	0.784	D
0.0858	0.0001	0.0212	0.0082	0.0198	0.0108	rs146776010	0.015	0.524	D
0.2281	0.0087	0	0.0009	0.1071	0	rs182802964	0.031	0.45	D
0	0	0	0	0	0	rs558211642	0.001	0.784	D
0.0007	0.0011	0	0.00002115	0	0.00007677	rs113015820	0.017	0.512	D
0.0659	0	0	0.0024	0.0121	0.0025	rs191740949	0.011	0.555	D
0.09	0.0362	0	0.0025	0	0.0011	rs117911884	0.019	0.501	D
0	0	0	0	0	0	.	0.037	0.43	D
0	0	0	0.00001689	0	0	rs200290505	0.007	0.614	D
0.0006	0	0	0.00003117	0.0012	0	rs564729213	0	0.912	D
0	0	0	0.00001539	0	0	rs746351550	0.001	0.784	D
0.0822	0.0025	0.0138	0.0083	0.0132	0.0059	rs61752057	0.001	0.784	D
0.1187	0.0249	0.0003	0.0004	0.0088	0.0007	rs80158178	0.045	0.465	D
0	0	0	0	0	0	.	0.003	0.682	D
0.0504	0.0002	0.0007	0.0012	0	0.0007	rs150702382	0.016	0.518	D
0.1311	0	0	0.0001	0.0094	0	rs190592136	0.001	0.784	D
0	0.0008	0	0.00001647	0	0.00006448	rs372603301	0.046	0.405	D
0.1257	0.0001	0	0.0005	0.0123	0.0013	rs142280693	0.001	0.784	D
0.001	0	0	0	0	0	rs139996840	0.001	0.912	D
0	0	0	0	0	0	rs113362509	0.033	0.443	D
0	0	0	0	0	0	rs768764962	0.005	0.912	D
0	0	0	0	0	0	.	0.041	0.421	D
0.1468	0.0831	0.0032	0.0022	0.0271	0.0212	rs35047625	0.001	0.784	D
0.0036	0.0007	0	0.0003	0.0011	0.0004	rs143403345	0.001	0.784	D
0.0869	0.0045	0	0.0008	0.0099	0.0009	rs143455680	0.018	0.506	D
0	0	0	0	0	0	.	0.002	0.721	D
0	0	0	0.00003193	0	0.00008372	rs780142749	0.035	0.437	D
0.0744	0.103	0.1435	0.1098	0.1213	0.0975	rs78034736	0	0.912	D
0.0308	0.0345	0.053	0.0399	0.0598	0.0817	rs73632975	0.039	0.424	D
0.0287	0.0332	0.0543	0.0381	0.0481	0.0836	rs73632976	0.017	0.512	D

Polyphen2_H	Polyphen2_H	Polyphen2_H	Polyphen2_H	Polyphen2_H	Polyphen2_H	LRT_score	LRT_convert	LRT_pred	MutationTas
0.763	0.899	P	0.245	0.619	B	0	0.629	D	0.996
1	0.899	D	0.999	0.971	D	0	0.843	D	1
1	0.899	D	0.992	0.79	D	.	.	.	1
.	.	.	.	.	.	.	.	.	1
0.999	0.764	D	0.982	0.739	D	0.003	0.349	N	0
1	0.899	D	0.951	0.672	D	0.14	0.183	N	0.703
0.999	0.899	D	0.923	0.774	D	0.003	0.351	N	0.621
1	0.899	D	1	0.971	D	.	.	.	0
0.99	0.615	D	0.799	0.563	P	0	0.843	D	1
0	0.026	B	0.001	0.04	B	.	.	.	1
1	0.899	D	0.954	0.797	D	0	0.843	D	1
1	0.899	D	0.999	0.971	D	0	0.843	D	1
0.745	0.412	P	0.276	0.384	B	0.973	0.078	U	1
0.9	0.476	P	0.286	0.388	B	.	.	.	1
0.129	0.254	B	0.078	0.271	B	.	.	.	1
0.99	0.615	D	0.827	0.576	P	0	0.629	D	1
0.993	0.637	D	0.84	0.584	P	0	0.843	D	1
0.988	0.604	D	0.973	0.713	D	.	.	.	1
0.99	0.615	D	0.885	0.61	P	0	0.629	D	1
0.944	0.512	P	0.321	0.421	B	0.539	0.116	N	1
0.001	0.067	B	0.007	0.121	B	0.055	0.227	N	0.513
0.997	0.689	D	0.814	0.57	P	0	0.629	D	0.973
1	0.899	D	0.997	0.85	D	0	0.843	D	1
1	0.899	D	0.997	0.85	D	0.008	0.312	N	1
0.958	0.53	D	0.682	0.517	P	0.248	0.156	N	1
0.974	0.622	D	0.416	0.608	B	0	0.843	D	1
0.01	0.144	B	0.006	0.112	B	0.739	0.098	N	1
0.189	0.277	B	0.097	0.288	B	.	.	.	0.998
1	0.899	D	0.999	0.916	D	0	0.441	D	0.691
0.001	0.067	B	0.012	0.149	B	0.056	0.225	N	1
0.997	0.689	D	0.991	0.782	D	0	0.843	D	1
0.328	0.314	B	0.127	0.311	B	.	.	.	0.836

0.974	0.559	D	0.969	0.703	D	0.001	0.393	N	.
0.998	0.715	D	0.873	0.602	P	0	0.504	D	1
0.164	0.295	B	0.027	0.252	B	0.004	0.343	N	1
0.983	0.764	D	0.954	0.739	D	0	0.843	D	1
0.965	0.899	D	0.468	0.875	P	0	0.843	D	1
0.999	0.764	D	0.87	0.6	P	0.007	0.314	N	1
0.465	0.374	P	0.136	0.38	B	0.927	0.084	N	1
0.973	0.556	D	0.932	0.65	D	0	0.559	D	1
0.999	0.764	D	0.947	0.667	D	0.003	0.359	N	0.966
0.931	0.499	P	0.747	0.541	P	0	0.843	D	1
1	0.899	D	0.998	0.875	D	0	0.843	D	1
0.965	0.899	D	0.889	0.797	P	0	0.559	D	1
1	0.899	D	0.999	0.916	D	.	.	.	1
0.999	0.764	D	0.849	0.736	P	0	0.457	D	0.998
0.514	0.355	P	0.218	0.36	B	0	0.537	D	1
1	0.899	D	0.984	0.745	D	0	0.843	D	1
0.997	0.689	D	0.897	0.619	P	0	0.843	D	1
0.002	0.112	B	0.008	0.127	B	0.014	0.287	N	0.963
0.998	0.715	D	0.971	0.708	D	0	0.843	D	1
0	0.026	B	0	0.013	B	.	.	.	1
1	0.899	D	0.962	0.69	D	0	0.843	D	1
1	0.899	D	0.995	0.85	D	0	0.843	D	1
1	0.899	D	0.996	0.916	D	0	0.843	D	1
0.928	0.496	P	0.666	0.512	P	0	0.629	D	1
0.528	0.464	P	0.09	0.373	B	.	.	.	0.583
0.005	0.119	B	0.009	0.133	B	0	0.629	D	1
1	0.899	D	1	0.971	D	.	.	.	1
0.993	0.689	D	0.96	0.686	D	0	0.843	D	1
1	0.899	D	1	0.971	D	0	0.843	D	1
0.291	0.306	B	0.013	0.154	B	0.032	0.018	N	1
0.998	0.715	D	0.809	0.568	P	0.018	0.277	N	0.998
0.946	0.514	P	0.756	0.544	P	.	.	.	0.999
0	0.026	B	0	0.013	B	.	.	.	1

0.998	0.715	D	0.921	0.639	D	0.002	0.387	N	0.986
1	0.899	D	0.987	0.85	D	0	0.523	D	1
0.999	0.764	D	0.98	0.732	D	0.002	0.369	N	0.992
1	0.899	D	0.855	0.591	P	0	0.629	U	0.927
0.718	0.403	P	0.39	0.424	B	0.659	0.104	N	1
0.686	0.396	P	0.25	0.373	B	0.002	0.381	N	0.995
0.992	0.689	D	0.906	0.626	P	0	0.481	D	0.872
0.994	0.647	D	0.725	0.532	P	.	.	.	0.637
0	0.026	B	0	0.013	B	.	.	.	1
1	0.899	D	1	0.971	D	0	0.843	D	1
0.004	0.112	B	0.001	0.063	B	0.548	0.053	N	1
0.999	0.764	D	0.934	0.652	D	0	0.559	D	1
0.188	0.276	B	0.034	0.212	B	.	.	.	1
0.997	0.715	D	0.928	0.699	D	.	.	.	1
0.975	0.561	D	0.913	0.631	D	.	.	.	0.963
0.001	0.342	B	0.002	0.296	B	0.029	0.255	N	0.983
1	0.899	D	0.973	0.713	D	0	0.445	D	0.99
0.987	0.6	D	0.91	0.629	D	0.676	0.103	N	0.851
0.832	0.442	P	0.625	0.497	P	0.158	0.178	N	0.69
0.607	0.715	P	0.146	0.774	B	0	0.454	D	0.972
0.964	0.764	D	0.3	0.659	B	0	0.504	D	1
0.998	0.715	D	0.992	0.79	D	0	0.843	D	1
0.987	0.6	D	0.873	0.602	P	0.165	0.175	N	1
1	0.899	D	0.945	0.664	D	0.001	0.419	D	0.964
0.626	0.382	P	0.67	0.513	P	0.058	0.225	N	1
0.991	0.622	D	0.992	0.79	D	0.007	0.315	N	1
0.005	0.119	B	0.023	0.188	B	0.177	0.172	N	0.999
0.003	0.102	B	0.003	0.08	B	0.061	0.222	N	1
0.18	0.274	B	0.046	0.233	B	0	0.513	D	0.686
1	0.899	D	0.967	0.699	D	0.005	0.33	N	0.996
0.003	0.102	B	0.002	0.063	B	.	.	.	1
0.194	0.278	B	0.045	0.231	B	0	0.454	N	0.939
0.998	0.715	D	0.854	0.591	P	0.004	0.345	N	0.92

1	0.899	D	0.972	0.971	D	0	0.843	D	1
0.997	0.689	D	0.879	0.606	P	0	0.843	U	1
0.206	0.282	B	0.085	0.279	B	0.037	0.244	N	1
0.991	0.622	D	0.857	0.593	P	0.123	0.189	N	0.992
1	0.899	D	0.998	0.875	D	0	0.629	D	1
1	0.899	D	1	0.971	D	0	0.629	D	1
1	0.899	D	0.94	0.658	D	0	0.629	D	1
1	0.899	D	0.999	0.916	D	0	0.559	D	0.988
0.675	0.471	P	0.39	0.451	B	0.012	0.292	N	1
0.007	0.131	B	0.003	0.08	B	.	.	.	1
1	0.899	D	0.998	0.875	D	.	.	.	0.998
1	0.899	D	0.997	0.916	D	0.083	0.207	N	0.979
1	0.899	D	0.988	0.764	D	0	0.504	D	1
0.977	0.566	D	0.498	0.458	P	0	0.629	D	0.654
0.991	0.622	D	0.721	0.531	P	.	.	.	1
1	0.899	D	1	0.971	D	0	0.629	D	1
0.302	0.308	B	0.109	0.298	B	0.001	0.391	N	0.996
0.023	0.499	B	0.015	0.441	B	0	0.559	D	0.951
0.999	0.764	D	0.997	0.85	D	0	0.481	D	1
0.997	0.689	D	0.804	0.565	P	0	0.537	U	1
0.75	0.413	P	0.454	0.444	P	0.08	0.21	U	0.993
1	0.899	D	0.999	0.916	D	0.788	0.067	N	1
0.29	0.306	B	0.241	0.369	B	0.018	0.276	N	0.999
0.998	0.899	D	0.879	0.651	P	0.123	0.189	N	0.527
0.574	0.368	P	0.146	0.323	B	0.08	0.023	N	1
0.966	0.542	D	0.844	0.586	P	0.002	0.384	N	1
1	0.899	D	0.975	0.736	D	0	0.629	D	1
0.954	0.524	P	0.759	0.545	P	0.003	0.355	N	0.755
0.963	0.537	D	0.178	0.341	B	0.23	0.159	U	1
1	0.899	D	0.983	0.742	D	0	0.559	D	0.785
0.462	0.764	P	0.327	0.628	B	0.016	0.28	N	0.864
0.001	0.067	B	0.001	0.04	B	0.108	0.026	U	1
1	0.899	D	0.982	0.739	D	0	0.843	D	1

1	0.899	D	0.989	0.818	D	0	0.843	D	1
0.487	0.35	P	0.122	0.308	B	.	.	.	0.86
1	0.899	D	0.998	0.875	D	0.029	0.254	N	1
0.679	0.536	P	0.281	0.666	B	0.004	0.338	N	0.737
1	0.899	D	1	0.971	D	0	0.843	D	1
0.976	0.564	D	0.626	0.498	P	0	0.449	D	1
0.954	0.524	P	0.374	0.419	B	0.241	0.157	N	0.956
0.153	0.265	B	0.246	0.371	B	0	0.629	D	1
0.925	0.552	P	0.621	0.496	P	0	0.629	D	1
0.73	0.481	P	0.273	0.383	B	0.037	0.244	N	1
0.456	0.344	P	0.115	0.303	B	.	.	.	0.999
1	0.899	D	1	0.971	D	0	0.629	D	1
0.961	0.535	D	0.541	0.47	P	0.206	0.165	N	1
0.977	0.566	D	0.796	0.562	P	0.001	0.402	N	0.994
0.016	0.161	B	0.015	0.162	B	.	.	.	1
0.028	0.184	B	0.014	0.158	B	0.001	0.405	N	1
0.979	0.571	D	0.646	0.504	P	0.001	0.422	D	0.823
0.563	0.365	P	0.081	0.275	B	0.004	0.342	N	0.865
1	0.899	D	0.999	0.916	D	0	0.843	D	1
0.792	0.473	P	0.507	0.523	P	0.916	0.085	N	1
0.889	0.535	P	0.022	0.223	B	0.662	0.104	U	0.769
0.185	0.276	B	0.101	0.292	B	0.002	0.387	U	0.598
1	0.899	D	1	0.971	D	.	.	.	1
0.998	0.715	D	0.986	0.754	D	0.101	0.199	N	0.999
1	0.899	D	0.999	0.916	D	0	0.843	D	1
0.99	0.615	D	0.756	0.544	P	0	0.843	D	1
1	0.899	D	1	0.971	D	0	0.629	D	0.998
0.552	0.363	P	0.346	0.409	B	0	0.629	D	1
0.995	0.899	D	0.921	0.875	D	0	0.629	D	0.919
1	0.899	D	0.969	0.703	D	0.001	0.406	N	1
0.985	0.592	D	0.507	0.46	P	0	0.504	D	1
0.675	0.393	P	0.367	0.416	B	0	0.629	D	0.999
0.987	0.6	D	0.886	0.611	P	0	0.843	D	1

0.256	0.296	B	0.052	0.241	B	0	0.46	D	0.527
0.966	0.542	D	0.973	0.713	D	0	0.629	D	1
1	0.899	D	0.999	0.916	D	0	0.843	D	1
0.697	0.398	P	0.493	0.456	P	0	0.843	D	1
1	0.899	D	0.995	0.818	D	0	0.843	D	1
1	0.899	D	0.999	0.916	D	0.298	0.146	N	1
.	.	.	.	.	.	.	.	.	1
0.928	0.496	P	0.66	0.51	P	0.01	0.299	N	0.882
0.999	0.764	D	0.918	0.636	D	0.004	0.341	U	0.898
0.407	0.564	B	0.331	0.518	B	0	0.629	D	0.926
0.297	0.307	B	0.172	0.337	B	0	0.843	D	1
.	.	.	.	.	.	0.331	0.141	U	1
0.995	0.764	D	0.847	0.679	P	0.029	0.255	N	1
0.993	0.637	D	0.968	0.701	D	0	0.843	D	1
0.292	0.306	B	0.039	0.223	B	0	0.457	D	0.79
1	0.899	D	0.998	0.875	D	0	0.629	D	1
0.478	0.348	P	0.16	0.331	B	.	.	.	0.993
0.002	0.09	B	0.001	0.04	B	0	0.005	N	1
0.629	0.383	P	0.286	0.388	B	0	0.473	N	1
0.561	0.365	P	0.016	0.166	B	.	.	.	0.998
0.84	0.445	P	0.767	0.549	P	0.014	0.287	N	0.999
1	0.899	D	0.958	0.683	D	0	0.504	D	0.992
0.812	0.435	P	0.416	0.432	B	0.392	0.045	N	1
0.191	0.288	B	0.056	0.286	B	0	0.537	D	1
0.272	0.301	B	0.075	0.268	B	0	0.497	D	0.765
0.736	0.511	P	0.45	0.527	P	0	0.629	D	1
0.698	0.46	P	0.155	0.422	B	0.211	0.164	N	0.934
0.718	0.403	P	0.349	0.41	B	0.867	0.071	N	1
0.998	0.715	D	0.667	0.512	P	0	0.843	D	1
0.245	0.294	B	0.232	0.366	B	0	0.537	D	1
0.514	0.355	P	0.326	0.402	B	0.183	0.17	N	0.999
0.874	0.461	P	0.224	0.363	B	.	.	.	1
0.27	0.523	B	0.095	0.43	B	0	0.843	D	1

0.999	0.764	D	0.974	0.715	D	0	0.463	D	0.998
0.995	0.657	D	0.791	0.559	P	0.004	0.341	N	1
0.001	0.067	B	0.005	0.104	B	0.013	0.289	N	0.622
0.827	0.44	P	0.416	0.432	B	0.002	0.379	N	1
0.001	0.067	B	0	0.149	B	0.058	0.224	N	1
0	0.026	B	0	0.013	B	0.964	0.077	N	0.685
0.998	0.715	D	0.871	0.601	P	.	.	.	1
0.732	0.408	P	0.561	0.477	P	0	0.481	D	0.96
0.767	0.899	P	0.258	0.682	B	0.172	0.173	N	1
0.46	0.345	P	0.098	0.289	B	0	0.002	N	1
0.267	0.299	B	0.086	0.28	B	0.173	0.173	N	1
1	0.899	D	0.997	0.85	D	0	0.523	D	1
1	0.899	D	0.981	0.736	D	0	0.559	D	0.982
0.897	0.474	P	0.925	0.643	D	0	0.629	D	1
0.01	0.144	B	0.002	0.063	B	0.005	0.327	N	1
0.996	0.67	D	0.939	0.657	D	0	0.843	D	1
0.999	0.899	D	0.997	0.971	D	0	0.843	D	1
1	0.899	D	0.993	0.797	D	0	0.843	D	1
0.051	0.21	B	0.024	0.19	B	0.048	0.233	N	1
1	0.899	D	1	0.971	D	0	0.843	D	1
0.004	0.112	B	0.007	0.121	B	0.02	0.271	N	0.952
1	0.899	D	0.999	0.916	D	0.093	0.203	U	1
0.988	0.604	D	0.942	0.66	D	0.019	0.273	N	0.515
.	.	.	.	.	.	.	.	.	0.804
0.9	0.53	P	0.762	0.664	P	0.005	0.335	N	0.932
0.019	0.167	B	0.014	0.158	B	.	.	.	0.999
1	0.899	D	0.998	0.875	D	0	0.843	D	0.58
0.935	0.503	P	0.567	0.479	P	0.007	0.319	N	1
0.087	0.261	B	0.03	0.42	B	.	.	.	1
0.083	0.232	B	0.008	0.127	B	0	0.629	U	0.582
0.991	0.622	D	0.686	0.518	P	.	.	.	1
0.999	0.764	D	0.931	0.649	D	.	.	.	0.995
0.18	0.581	B	0.147	0.566	B	0	0.629	D	0.995

0.857	0.453	P	0.461	0.446	P		0.066	0.218	N	1
1	0.899	D	1	0.971	D	.	.		.	1
1	0.899	D	0.996	0.832	D		0.003	0.356	N	0.823
0.084	0.233	B	0.043	0.229	B		0.002	0.386	U	1
0.425	0.336	B	0.092	0.285	B	.	.		.	1
0.154	0.265	B	0.16	0.331	B	.	.		.	1
0.155	0.265	B	0.088	0.282	B	.	.		.	1
0.001	0.067	B	0.009	0.133	B		0.005	0.328	N	0.951
0.728	0.899	P	0.181	0.655	B		0.503	0.119	N	1
1	0.899	D	1	0.971	D		0	0.504	D	1
1	0.899	D	0.948	0.719	D		0.018	0.275	N	1
1	0.899	D	0.996	0.832	D		0	0.629	D	1
0.949	0.899	P	0.81	0.916	P		0	0.843	D	1
0.859	0.453	P	0.435	0.485	B		0	0.629	D	1
0.999	0.764	D	0.954	0.677	D		0.001	0.418	D	0.64
0.981	0.609	D	0.656	0.57	P		0	0.002	N	1
0.006	0.163	B	0.002	0.063	B		0.99	0.08	N	1
0.013	0.222	B	0.004	0.158	B		0.356	0.137	N	1
0.999	0.899	D	0.887	0.916	P		0.529	0.117	N	0.992
0.991	0.622	D	0.882	0.608	P		0	0.629	D	0.999
0.999	0.764	D	0.992	0.832	D		0.001	0.431	U	1
0.978	0.568	D	0.969	0.703	D		0	0.843	D	0.994
1	0.899	D	0.959	0.685	D		0	0.523	D	0.002
1	0.899	D	0.957	0.682	D		0	0.843	D	1
0.789	0.426	P	0.222	0.362	B		0.015	0.284	N	0.591
0.998	0.715	D	0.994	0.807	D		0	0.843	D	0.98
1	0.899	D	0.996	0.832	D		0	0.466	D	1
1	0.899	D	0.993	0.797	D		0.001	0.411	U	0
0.314	0.311	B	0.248	0.372	B		0	0.629	U	0.002
0.931	0.585	P	0.873	0.609	P		0	0.843	U	0

MutationTas	MutationTas	MutationAss	MutationAss	MutationAss	FATHMM_sc	FATHMM_co	FATHMM_pr	PROVEAN_s	PROVEAN_c
0.428	D	1.905	0.51	L	6.89	0.045	T	-3.1	0.635
0.81	D	2.975	0.857	M	-2.73	0.907	D	-8.71	0.976
0.09	N	0.695	0.181	N	1.88	0.239	T	-0.29	0.117
0.09	P	0.805	0.203	L	1.8	0.269	T	-0.57	0.172
0.588	P	.	.	.	0.56	0.546	T	-5	0.823
0.334	D	0.805	0.203	L	1.68	0.272	T	-3	0.623
0.308	N	2.65	0.778	M	3.64	0.043	T	-3.52	0.684
0.81	P	2.39	0.691	M	4.72	0.017	T	-7.99	0.964
0.81	D	2.815	0.823	M	-0.95	0.754	T	-5.38	0.849
0.09	N	.	.	.	-1.08	0.771	T	-0.21	0.103
0.81	D	2.845	0.829	M	-1.56	0.818	D	-5.13	0.832
0.81	D	3.19	0.892	M	-3.09	0.926	D	-5.07	0.828
0.09	N	2.305	0.662	M	2.51	0.143	T	-2.92	0.611
0.09	N	1.7	0.44	L	5.3	0.011	T	-0.77	0.214
0.09	N	1.935	0.52	L	2.86	0.105	T	-0.81	0.223
0.81	D	2.095	0.583	M	0.44	0.566	T	-1.75	0.414
0.81	D	2.52	0.738	M	.	.	.	-2.18	0.548
0.09	N	1.6	0.409	L	8.52	0.002	T	-1.51	0.368
0.81	D	0.895	0.225	L	0.7	0.515	T	-1.45	0.356
0.09	N	1.65	0.424	L	2.3	0.17	T	-1.48	0.362
0.316	N	0.955	0.24	L	.	.	.	-2.07	0.473
0.39	D	2.28	0.651	M	1.71	0.267	T	-1.64	0.393
0.81	D	2.215	0.627	M	-1.18	0.783	T	-6.93	0.942
0.09	N	2.955	0.853	M	3.36	0.059	T	-4.98	0.821
0.09	N	2.825	0.825	M	0.7	0.515	T	-4.78	0.819
0.81	D	2.25	0.64	M	.	.	.	-1.39	0.372
0.09	N	1.65	0.424	L	1.85	0.248	T	-0.32	0.253
0.81	D	3.315	0.908	M	1.97	0.221	T	-7.95	0.963
0.333	D	4.04	0.971	H	7.08	0.005	T	-5.7	0.874
0.199	N	1.68	0.433	L	6.71	0.005	T	-4.16	0.753
0.81	D	2.525	0.74	M	-0.98	0.758	T	-7.78	0.959
0.35	D	0.55	0.145	N	-1.81	0.84	D	-0.42	0.142

.	.	2.36	0.681	M	.	.	.	-2.62	0.563
0.511	D	2.815	0.823	M	.	.	.	-6.06	0.899
0.81	D	1.59	0.404	L	1.54	0.301	T	-1.73	0.412
0.588	D	3.56	0.935	H	-0.62	0.719	T	-5.69	0.873
0.09	N	2.56	0.75	M	-0.31	0.68	T	-1.62	0.793
0.548	D	2.82	0.824	M	-1.2	0.785	T	-4.41	0.774
0.182	N	1.7	0.44	L	2.8	0.111	T	-1.52	0.397
0.81	D	3.415	0.92	M	0.83	0.478	T	-2.62	0.608
0.385	D	2.38	0.688	M	-4.38	0.974	D	-8.44	0.974
0.81	D	1.78	0.463	L	1.24	0.747	T	-3.73	0.751
0.588	D	2.25	0.64	M	0.71	0.512	T	-4.18	0.755
0.81	D	3.8	0.955	H	-1.48	0.812	T	-8.93	0.98
0.81	D	2.95	0.852	M	-0.29	0.677	T	-5.17	0.864
0.444	D	3.115	0.881	M	-3.41	0.943	D	-6.43	0.913
0.81	D	2.34	0.674	M	3.52	0.049	T	-2.62	0.563
0.81	D	3.545	0.934	H	1.03	0.405	T	-7.24	0.943
0.81	D	2.08	0.576	M	1.3	0.356	T	-2.75	0.769
0.383	D	2.175	0.612	M	1.12	0.387	T	-2.9	0.608
0.588	D	2.485	0.726	M	1.25	0.365	T	-3.57	0.712
0.09	N	.	.	.	.	.	.	-0.74	0.208
0.81	D	2.325	0.668	M	.	.	.	-3.12	0.742
0.81	D	2.985	0.859	M	1.5	0.418	T	-3.78	0.715
0.81	D	2.87	0.836	M	0.74	0.505	T	-3.84	0.737
0.488	D	2.81	0.821	M	2.04	0.229	T	-4.06	0.745
0.311	N	0.59	0.155	N	-1.68	0.829	D	-0.1	0.129
0.81	D	3.11	0.88	M	-1.01	0.762	T	-2.21	0.496
0.81	D	2.74	0.803	M	-5.21	0.989	D	-5.53	0.86
0.81	D	3.795	0.955	H	-3.39	0.942	D	-2.42	0.531
0.81	D	2.695	0.791	M	-3.25	0.935	D	-4.87	0.822
0.09	P	-0.695	0.019	N	0.27	0.592	T	-0.8	0.221
0.224	N	2.495	0.729	M	2.47	0.148	T	-3.89	0.728
0.214	N	.	.	.	4.19	0.027	T	-5.37	0.849
0.09	N	0	0.065	N	1.91	0.233	T	-0.54	0.166

0.246	N	2.205	0.624	M	0.39	0.577	T	-1.33	0.385
0.182	N	2.455	0.715	M	-0.41	0.694	T	-5.31	0.844
0.416	D	2.81	0.821	M	0.58	0.542	T	-2.46	0.537
0.271	N	0.895	0.225	L	1.08	0.394	T	-3.32	0.66
0.09	N	2.045	0.562	M	3.18	0.075	T	-1.24	0.314
0.424	D	0.945	0.238	L	0.98	0.421	T	-1.75	0.804
0.361	D	2.425	0.705	M	2.61	0.272	T	-4.84	0.81
0.81	D	.	.	.	-5.53	0.992	D	-0.65	0.189
0.09	N	0	0.065	N	0.26	0.593	T	-0.58	0.174
0.548	D	2.78	0.814	M	2.91	0.1	T	-5.29	0.843
0.09	N	1.795	0.474	L	3.23	0.07	T	-3.62	0.695
0.524	D	2.7	0.793	M	2.75	0.16	T	-2.27	0.506
0.81	D	.	.	.	2.63	0.129	T	-0.16	0.095
0.81	D	3.705	0.948	H	0.1	0.614	T	-2.84	0.598
0.26	N	3.425	0.921	M	2.01	0.213	T	-5.74	0.877
0.399	D	1.525	0.387	L	2.33	0.211	T	-2.3	0.579
0.242	N	3.115	0.881	M	-0.74	0.732	T	-4.13	0.751
0.352	D	1.15	0.294	L	-3.01	0.922	D	-1.21	0.307
0.333	D	1.565	0.397	L	1	0.414	T	-4.48	0.779
0.441	D	1.1	0.281	L	0.47	0.561	T	-0.94	0.251
0.81	D	3.745	0.951	H	1.11	0.389	T	-3.27	0.687
0.81	D	1.905	0.51	L	2.27	0.174	T	-1.45	0.356
0.09	N	1.75	0.456	L	6.27	0.006	T	-4.51	0.782
0.384	D	2.19	0.618	M	3.48	0.722	T	-2.63	0.674
0.272	N	1.43	0.36	L	1.5	0.312	T	-3.09	0.634
0.506	D	2.71	0.795	M	3.17	0.076	T	-2.43	0.532
0.22	N	2.57	0.754	M	1.7	0.269	T	-2.67	0.571
0.09	N	0.55	0.145	N	.	.	.	-0.12	0.172
0.333	D	.	.	.	1.83	0.248	T	-2.15	0.496
0.43	D	2.815	0.823	M	2.12	0.197	T	-3.42	0.672
0.09	N	.	.	.	1.08	0.394	T	-1.18	0.301
0.372	D	2.065	0.569	M	0.91	0.449	T	-0.93	0.249
0.366	D	2.32	0.666	M	2.45	0.15	T	-3.1	0.635

0.588	D	2.67	0.784	M	1.31	0.354	T	-4.71	0.812
0.81	D	2.05	0.567	M	-2.35	0.881	D	-4.53	0.783
0.09	N	2.075	0.572	M	-0.25	0.67	T	-1.03	0.27
0.239	N	1.87	0.498	L	8.65	0.002	T	-5.56	0.863
0.81	D	2.31	0.663	M	-0.17	0.656	T	-2.73	0.58
0.81	D	4.115	0.975	H	3.48	0.052	T	-7.6	0.953
0.588	D	.	.	.	3.81	0.038	T	-5.34	0.846
0.407	D	1.1	0.281	L	-0.12	0.646	T	-3.14	0.64
0.09	N	0.975	0.246	L	0	0.626	T	0.26	0.057
0.09	N	0.69	0.17	N	4.18	0.028	T	-0.15	0.144
0.449	D	1.77	0.46	L	-2.3	0.877	D	-4.94	0.818
0.395	D	2.36	0.681	M	-2.24	0.916	D	-3.69	0.964
0.81	D	3.165	0.888	M	-2.03	0.897	D	-3.86	0.724
0.33	D	0	0.065	N	-1.32	0.798	T	1.37	0.009
0.09	N	0	0.065	N	0.54	0.549	T	-1.5	0.366
0.81	D	3.245	0.9	M	-2.03	0.856	D	-10.34	0.99
0.429	D	1.955	0.53	M	2.36	0.161	T	-1.5	0.366
0.377	D	2.96	0.854	M	2.26	0.229	T	-2.17	0.49
0.53	D	1.76	0.459	L	-0.25	0.67	T	-4.76	0.803
0.524	D	2.475	0.721	M	0.05	0.619	T	-1.72	0.408
0.238	N	1.875	0.501	L	1.57	0.293	T	0.3	0.043
0.81	D	1.85	0.492	L	1.06	0.398	T	-2.13	0.483
0.457	D	1.935	0.52	L	0.76	0.499	T	-2.2	0.495
0.419	D	0.55	0.145	N	1.06	0.398	T	-1.42	0.427
0.09	P	1.1	0.281	L	.	.	.	0.07	0.061
0.09	N	1.735	0.45	L	.	.	.	-5.12	0.831
0.81	D	2.815	0.823	M	-2.42	0.886	D	-2.23	0.516
0.34	D	0.635	0.16	N	0.18	0.604	T	-2.79	0.59
0.192	N	1.7	0.44	L	-0.26	0.672	T	-2.17	0.49
0.396	D	3.68	0.946	H	3.68	0.042	T	-3.53	0.714
0.354	D	2.765	0.81	M	-3.35	0.94	D	-1.91	0.468
0.09	N	0.895	0.225	L	-0.18	0.658	T	-1.56	0.377
0.81	D	2.8	0.819	M	1.34	0.348	T	-4.47	0.778

0.53	D	2.545	0.745	M	2.71	0.121	T	-3.05	0.629
0.81	D	.	.	.	-1.04	0.766	T	-4.73	0.8
0.81	D	1.625	0.417	L	3.29	0.065	T	-4.75	0.802
0.338	D	1.245	0.315	L	-2.18	0.867	D	-3.37	0.666
0.81	D	3.125	0.882	M	-0.81	0.74	T	-6.5	0.916
0.588	D	3.155	0.887	M	-2.92	0.929	D	-4.24	0.76
0.38	D	2.255	0.644	M	-2.92	0.918	D	-1.57	0.559
0.588	D	1.58	0.399	L	-3.72	0.954	D	-4.29	0.764
0.81	D	2.595	0.761	M	0.17	0.605	T	-3.52	0.684
0.09	N	0.15	0.089	N	1.07	0.418	T	-1.28	0.322
0.211	N	0	0.065	N	-2.45	0.889	D	-0.15	0.093
0.81	D	3.535	0.933	H	8.67	0.001	T	-7.46	0.949
0.81	D	0.975	0.246	L	0.2	0.601	T	-4.24	0.76
0.236	N	1.34	0.335	L	0.55	0.547	T	-1.23	0.312
0.09	N	.	.	.	0.66	0.524	T	-2.62	0.563
0.09	N	0.345	0.112	N	2.01	0.213	T	2.05	0.004
0.81	D	3.37	0.915	M	-0.54	0.71	T	-8	0.965
0.292	N	2.32	0.666	M	0.83	0.478	T	-2.71	0.577
0.524	D	2.135	0.597	M	0.79	0.491	T	-2.8	0.592
0.182	N	0	0.065	N	3.02	0.09	T	-1.34	0.34
0.294	N	0.345	0.112	N	-1.72	0.833	D	-0.37	0.156
0.309	N	1.3	0.327	L	6.99	0.005	T	-5.29	0.843
0.81	D	3.77	0.953	H	3.04	0.088	T	-4.87	0.812
0.456	D	1.95	0.526	M	.	.	.	-2.19	0.493
0.81	D	4.52	0.991	H	-7.03	0.998	D	-7.85	0.96
0.81	D	2.72	0.798	M	1.33	0.35	T	-3.15	0.641
0.449	D	4.055	0.971	H	-5.15	0.988	D	-7.52	0.951
0.81	D	2.325	0.668	M	4.07	0.031	T	-3.07	0.632
0.415	D	2.455	0.715	M	2.99	0.093	T	-2.98	0.62
0.81	D	3.005	0.862	M	-2.81	0.911	D	-4.81	0.807
0.499	D	1.645	0.421	L	-2.22	0.871	D	-3.23	0.65
0.46	D	1.59	0.404	L	0.64	0.529	T	-3.43	0.673
0.588	D	2.695	0.791	M	-0.1	0.643	T	-7.75	0.958

0.319	D		0.695	0.181	N	2.86	0.652	T	-0.11	0.088
0.588	D		2.39	0.691	M	-4.09	0.966	D	-1.53	0.372
0.81	D		4.295	0.983	H	-0.49	0.704	T	-6.78	0.928
0.81	D		1.87	0.498	L	-3.25	0.935	D	-1.81	0.425
0.81	D	.	.	.		-2.57	0.897	D	-5.51	0.859
0.497	D		3.5	0.929	M	-0.3	0.679	T	-6.75	0.927
0.09	N	.	.	.		1.53	0.304	T	0.02	0.068
0.358	D		2.485	0.726	M	0.77	0.54	T	-2.12	0.481
0.361	D		2.38	0.688	M	2.38	0.158	T	-5.48	0.857
0.272	N		2.275	0.649	M	4.33	0.024	T	-1.68	0.401
0.588	D		2.23	0.633	M	2.64	0.128	T	-4.24	0.76
0.09	N		2.05	0.567	M	-0.13	0.648	T	-2.06	0.471
0.09	N		2.585	0.758	M	-0.32	0.682	T	-4.2	0.757
0.548	D		0.975	0.246	L	0.53	0.551	T	-3	0.68
0.297	N		2.19	0.618	M	-0.02	0.629	T	-4.64	0.797
0.81	D		3.325	0.91	M	2.23	0.759	T	-6.62	0.936
0.419	D		2.19	0.618	M	-1.88	0.893	D	-2.32	0.514
0.09	N		1.1	0.281	L	-0.59	0.716	T	-1.39	0.344
0.537	D		1.995	0.543	M	-0.43	0.697	T	-0.18	0.098
0.222	N		0	0.065	N	1.47	0.32	T	-0.65	0.189
0.81	D		2.03	0.557	M	-0.4	0.693	T	-6.33	0.909
0.81	D		1.87	0.498	L	3.53	0.049	T	-4.97	0.82
0.09	N	.	.	.		-1.84	0.842	D	-2.34	0.518
0.81	D		1.475	0.372	L	0.96	0.429	T	-2.17	0.49
0.341	D		0.205	0.094	N	-1.47	0.811	T	-1.07	0.946
0.548	D		1.845	0.488	L	0.86	0.496	T	-2.07	0.473
0.27	N		2.285	0.654	M	1.69	0.27	T	-3.18	0.644
0.09	N		1.385	0.346	L	1.91	0.233	T	0.2	0.049
0.81	D		2.435	0.708	M	1	0.414	T	-6.77	0.928
0.501	D		0.74	0.19	N	-0.5	0.705	T	-3.48	0.679
0.231	N		0.695	0.181	N	-3.07	0.925	D	-2.21	0.496
0.09	N		0.735	0.189	N	0.8	0.488	T	-1.61	0.387
0.588	D		2.41	0.699	M	3.22	0.087	T	-2.77	0.587

0.224	N	2.24	0.636	M	0.46	0.563	T	-5.13	0.832
0.81	D	-0.345	0.033	N	-0.72	0.73	T	-0.6	0.178
0.327	D	0.325	0.104	N	-2.44	0.888	D	-0.35	0.129
0.81	D	1.1	0.281	L	1.7	0.269	T	-1.2	0.316
0.09	N	0	0.065	N	2.28	0.173	T	-0.71	0.223
0.302	N	0.46	0.13	N	3.11	0.082	T	-0.13	0.09
0.81	D	2.625	0.771	M	.	.	.	-3.92	0.731
0.81	D	1.155	0.297	L	.	.	.	-0.93	0.249
0.09	N	0.805	0.203	L	2.9	0.107	T	-0.72	0.219
0.09	N	1.91	0.513	L	2.17	0.19	T	-1.96	0.454
0.09	N	0.805	0.203	L	3.47	0.052	T	-1.94	0.45
0.81	D	2.04	0.558	M	-2.84	0.913	D	-2.63	0.564
0.398	D	1.42	0.358	L	-0.28	0.676	T	-3.51	0.683
0.508	D	1.85	0.492	L	1.41	0.334	T	-1.87	0.437
0.09	N	2.275	0.649	M	-0.76	0.734	T	-1.74	0.412
0.81	D	1.545	0.392	L	-1.04	0.766	T	-5.06	0.827
0.548	D	2.465	0.718	M	0.15	0.616	T	-2.64	0.566
0.588	D	2.42	0.702	M	0.86	0.505	T	-2.86	0.601
0.09	N	2.56	0.75	M	2.26	0.176	T	-3.21	0.648
0.81	D	3.845	0.958	H	2.13	0.196	T	-8.87	0.979
0.264	N	-0.965	0.012	N	1.23	0.369	T	0.83	0.018
0.81	D	2.895	0.841	M	-0.49	0.704	T	-1.91	0.973
0.318	D	1.795	0.474	L	0.13	0.61	T	-3.19	0.646
0.345	D	0	0.065	N	-0.75	0.733	T	-0.24	0.108
0.401	D	1.97	0.535	M	0.35	0.621	T	-2.74	0.782
0.463	D	0.695	0.181	N	0.85	0.471	T	-0.51	0.16
0.311	N	2.175	0.612	M	0.79	0.491	T	-5.73	0.876
0.588	D	1.955	0.53	M	.	.	.	-2.04	0.468
0.09	N	0	0.065	N	4.01	0.219	T	-0.6	0.178
0.81	D	2.075	0.572	M	0.48	0.56	T	0.4	0.037
0.09	N	0.55	0.145	N	1.73	0.264	T	-0.45	0.148
0.81	D	2.645	0.776	M	-2.25	0.873	D	-1.52	0.389
0.427	D	2.92	0.846	M	-1.37	0.837	T	-5.1	0.83

0.09	N	3.125	0.882	M	1.73	0.264	T	-3.9	0.729
0.09	N	3.525	0.932	H	2.08	0.202	T	-5.65	0.87
0.288	N	0.805	0.203	L	0.67	0.522	T	-1.64	0.393
0.2	N	0.695	0.181	N	-1.23	0.79	T	-1.06	0.277
0.09	P	0.61	0.157	N	2.11	0.199	T	-2.57	0.555
0.09	N	2.72	0.798	M	5.73	0.008	T	-7.07	0.939
0.09	N	0	0.065	N	3.35	0.06	T	-0.46	0.15
0.377	D	1.445	0.365	L	2.08	0.202	T	-1.72	0.408
0.09	N	1.1	0.281	L	2.57	0.247	T	-1.88	0.644
0.482	D	1.965	0.534	M	1.28	0.37	T	-6.45	0.922
0.588	D	2.585	0.758	M	-2.52	0.894	D	-4.75	0.802
0.81	D	2.275	0.649	M	0.81	0.485	T	-5.95	0.891
0.81	D	2.525	0.74	M	0.99	0.418	T	-3.26	0.654
0.588	D	2.475	0.721	M	0.36	0.579	T	-5.11	0.831
0.328	D	2.36	0.681	M	2.12	0.197	T	-2.39	0.526
0.09	N	0.695	0.181	N	1.31	0.354	T	-4.09	0.748
0.09	N	0.69	0.17	N	4.85	0.015	T	-2.47	0.539
0.09	N	1.01	0.254	L	-0.16	0.654	T	-3.66	0.7
0.415	D	3.96	0.966	H	-3.2	0.948	D	-5.9	0.915
0.455	D	0.345	0.112	N	1.59	0.288	T	-0.69	0.197
0.81	D	2.24	0.636	M	-4.03	0.972	D	-3.58	0.761
0.42	D	1.7	0.44	L	.	.	.	-1.94	0.45
0.444	P	2.34	0.674	M	2.41	0.307	T	-4.63	0.827
0.524	D	2.36	0.681	M	.	.	.	-3.06	0.65
0.324	D	1.79	0.469	L	2.18	0.189	T	-1.58	0.381
0.396	D	2.28	0.651	M	-0.05	0.634	T	-1.29	0.332
0.511	D	2.75	0.806	M	2.25	0.178	T	-5.56	0.863
0.588	P	4.085	0.973	H	-3.86	0.959	D	-5.43	0.853
0.446	P	1.4	0.355	L	-3.28	0.937	D	-4.77	0.804
0.588	P	3.21	0.895	M	-3.76	0.956	D	-6.7	0.925

PROVEAN_p	VEST3_score	VEST3_ranks	MetaSVM_s	MetaSVM_ra	MetaSVM_p	MetaLR_scor	MetaLR_ranl	MetaLR_pre	M-CAP_score
D	0.64	0.657	-1.157	0.008	T	0.008	0.028	T	.
D	0.93	0.959	0.806	0.945	D	0.83	0.943	D	.
N	0.34	0.419	-1.048	0.149	T	0.059	0.246	T	.
N	0.234	0.287	-0.936	0.433	T	0	0	T	0.013
D	0.263	0.32	-0.998	0.304	T	0.002	0.007	T	.
D	0.34	0.401	-1.035	0.19	T	0.011	0.04	T	.
D	0.547	0.586	-1.226	0	T	0.001	0.003	T	.
D	0.521	0.563	-0.85	0.52	T	0	0	T	.
D	0.885	0.873	0.068	0.836	D	0.532	0.827	D	0.07
N	0.105	0.111	-1.003	0.29	T	0.116	0.411	T	.
D	0.968	0.969	0.575	0.916	D	0.731	0.908	D	0.184
D	0.974	0.975	0.858	0.951	D	0.886	0.962	D	0.338
D	0.128	0.146	-1.076	0.082	T	0.013	0.051	T	0.006
N	0.294	0.354	-0.876	0.501	T	0.001	0.004	T	.
N	0.17	0.221	-0.927	0.447	T	0.008	0.026	T	.
N	0.601	0.626	-0.768	0.57	T	0.171	0.512	T	0.027
N	0.58	0.614	-0.469	0.698	T	0.304	0.676	T	0.006
N	0.152	0.202	-0.835	0.53	T	0	0.001	T	.
N	0.687	0.693	-0.76	0.574	T	0.189	0.54	T	.
N	0.046	0.026	-1.061	0.114	T	0.015	0.06	T	.
N	0.111	0.12	-0.995	0.313	T	0.081	0.319	T	0.01
N	0.588	0.616	-1.057	0.124	T	0.106	0.388	T	0.014
D	0.964	0.963	0.258	0.869	D	0.619	0.866	D	0.232
D	0.783	0.774	-1.214	0.001	T	0.004	0.014	T	.
D	0.357	0.426	-0.832	0.532	T	0.11	0.398	T	.
N	0.846	0.848	-0.805	0.549	T	0.259	0.63	T	0.004
N	0.019	0.009	-0.99	0.327	T	0.002	0.007	T	.
D	0.135	0.156	-1.048	0.15	T	0.083	0.326	T	0.004
D	0.295	0.407	-1.063	0.109	T	0.002	0.007	T	.
D	0.242	0.296	-0.945	0.418	T	0.002	0.007	T	.
D	0.863	0.85	0.379	0.888	D	0.637	0.873	D	0.166
N	0.115	0.127	-0.909	0.47	T	0.075	0.302	T	.

D	0.313	0.761	-0.45	0.704	T	0.271	0.643	T	.
D	0.829	0.822	-0.371	0.729	T	0.212	0.572	T	.
N	0.427	0.483	-1.048	0.148	T	0.047	0.2	T	0.009
D	0.843	0.83	0.546	0.912	D	0.651	0.879	D	0.052
N	0.42	0.613	-0.795	0.555	T	0.146	0.47	T	.
D	0.73	0.728	0.167	0.854	D	0.586	0.852	D	0.109
N	0.387	0.456	-1.024	0.225	T	0.006	0.021	T	.
D	0.86	0.851	-0.848	0.521	T	0.032	0.139	T	.
D	0.701	0.739	0.413	0.893	D	0.774	0.923	D	.
D	0.698	0.702	-0.276	0.757	T	0.306	0.677	T	.
D	0.817	0.805	-0.477	0.695	T	0.343	0.708	T	0.043
D	0.904	0.894	-0.398	0.721	T	0.093	0.354	T	.
D	0.402	0.582	-0.576	0.658	T	0.081	0.32	T	.
D	0.525	0.566	0.742	0.937	D	0.841	0.947	D	0.239
D	0.328	0.463	-1.133	0.016	T	0.032	0.138	T	0.033
D	0.703	0.706	-0.497	0.688	T	0.084	0.33	T	.
D	0.821	0.809	-1.011	0.266	T	0.045	0.193	T	.
D	0.291	0.35	-1.005	0.285	T	0.015	0.06	T	.
D	0.194	0.353	-1.015	0.253	T	0.038	0.163	T	.
N	0.039	0.019	-1.012	0.264	T	0.069	0.282	T	0.002
D	0.833	0.823	-0.119	0.797	T	0.413	0.761	T	0.112
D	0.94	0.949	0.181	0.856	D	0.553	0.836	D	0.016
D	0.809	0.825	-0.684	0.612	T	0.066	0.272	T	.
D	0.707	0.709	-0.881	0.497	T	0.103	0.379	T	.
N	0.267	0.354	-0.723	0.593	T	0.017	0.07	T	.
N	0.855	0.904	-0.297	0.751	T	0.429	0.772	T	0.095
D	0.986	0.991	0.726	0.935	D	0.942	0.981	D	.
N	0.678	0.925	1.065	0.985	D	0.927	0.976	D	0.208
D	0.26	0.575	0.825	0.947	D	0.861	0.954	D	0.265
N	0.055	0.086	-1.089	0.057	T	0	0	T	.
D	0.49	0.537	-1.162	0.007	T	0.014	0.054	T	.
D	0.399	0.588	-1.035	0.189	T	0.015	0.06	T	0.001
N	0.124	0.14	-1.028	0.211	T	0.024	0.101	T	0.001

N	0.144	0.239	-0.79	0.558	T	0.101	0.374	T	.
D	0.467	0.606	-0.569	0.661	T	0.3	0.672	T	.
N	0.224	0.37	-0.601	0.648	T	0.183	0.531	T	.
D	0.722	0.722	-0.986	0.336	T	0.062	0.26	T	0.006
N	0.159	0.191	-0.968	0.377	T	0.017	0.072	T	0.005
N	0.638	0.655	-1.027	0.213	T	0.07	0.286	T	0.086
D	0.267	0.422	-1	0.299	T	0.045	0.195	T	.
N	0.425	0.481	-0.718	0.596	T	0.178	0.524	T	.
N	0.159	0.191	-1.004	0.288	T	0.125	0.43	T	0.014
D	0.76	0.754	-1.005	0.284	T	0.097	0.365	T	0.009
D	0.109	0.117	-0.955	0.402	T	0.006	0.019	T	.
N	0.59	0.637	-1.132	0.017	T	0.054	0.23	T	.
N	0.048	0.029	-1.071	0.092	T	0.015	0.062	T	.
D	0.671	0.722	-0.042	0.814	T	0.202	0.559	T	.
D	0.347	0.408	-0.933	0.437	T	0.156	0.488	T	0.012
N	0.582	0.686	-1.084	0.066	T	0.05	0.213	T	0.023
D	0.748	0.744	-0.167	0.785	T	0.58	0.849	D	0.12
N	0.4	0.459	0.677	0.929	D	0.812	0.937	D	0.811
D	0.351	0.412	-0.755	0.577	T	0.135	0.45	T	0.035
N	0.717	0.718	-0.783	0.562	T	0.19	0.542	T	.
D	0.23	0.426	-0.222	0.771	T	0.281	0.653	T	.
N	0.448	0.501	-1.115	0.026	T	0.077	0.309	T	.
D	0.785	0.776	-0.935	0.433	T	0.004	0.011	T	.
D	0.286	0.35	-0.29	0.753	T	0.391	0.745	T	.
D	0.248	0.303	-1.088	0.059	T	0.054	0.229	T	0.015
N	0.823	0.819	-0.824	0.537	T	0.084	0.328	T	0.026
D	0.413	0.471	-1.063	0.111	T	0.041	0.175	T	.
N	0.348	0.409	-1.009	0.271	T	0.024	0.1	T	0.006
N	0.49	0.537	-1.084	0.066	T	0.032	0.135	T	.
D	0.223	0.274	-1.098	0.043	T	0.067	0.276	T	.
N	.	.	-1.048	0.15	T	0.013	0.051	T	.
N	0.093	0.109	-1.073	0.086	T	0.054	0.228	T	0.015
D	0.593	0.62	-1.081	0.071	T	0.054	0.228	T	.

D	0.786	0.782	-0.854	0.517	T	0.148	0.474	T	.
D	0.75	0.745	0.375	0.887	D	0.679	0.889	D	0.305
N	0.295	0.426	-0.988	0.331	T	0.093	0.354	T	.
D	0.073	0.062	-0.82	0.54	T	0	0.001	T	.
D	0.892	0.937	-0.525	0.678	T	0.204	0.562	T	.
D	0.923	0.915	-0.95	0.41	T	0.068	0.278	T	.
D	0.121	0.893	-1.026	0.218	T	0.058	0.243	T	.
D	0.698	0.833	-0.629	0.636	T	0.068	0.279	T	.
N	0.352	0.473	-0.903	0.477	T	0.131	0.442	T	.
N	0.047	0.028	-0.909	0.47	T	0.006	0.021	T	0.01
D	0.246	0.301	0.525	0.909	D	0.722	0.905	D	0.179
D	0.35	0.639	0.742	0.937	D	0.828	0.942	D	0.043
D	0.75	0.745	0.178	0.855	D	0.486	0.804	T	.
N	0.348	0.409	-0.547	0.669	T	0.065	0.267	T	.
N	0.321	0.382	-1.126	0.02	T	0.003	0.008	T	.
D	0.997	0.999	0.695	0.931	D	0.787	0.928	D	0.091
N	0.03	0.034	-1.123	0.022	T	0.011	0.04	T	.
N	0.641	0.693	-1.054	0.133	T	0.074	0.297	T	0.011
D	0.779	0.771	-0.868	0.507	T	0.026	0.113	T	.
N	0.516	0.565	-0.188	0.78	T	0.411	0.759	T	0.025
N	0.162	0.294	-1.049	0.146	T	0.086	0.335	T	0.002
N	0.663	0.674	-1.104	0.036	T	0.02	0.084	T	.
N	0.221	0.271	-0.953	0.404	T	0.12	0.42	T	0.01
N	0.591	0.64	-0.886	0.493	T	0.156	0.487	T	0.018
N	0.166	0.2	-1.066	0.103	T	0	0	T	.
D	0.263	0.32	-1.057	0.126	T	0.098	0.366	T	.
N	0.513	0.667	0.583	0.917	D	0.809	0.935	D	0.19
D	0.603	0.648	-0.971	0.369	T	0.01	0.035	T	.
N	0.21	0.258	-0.904	0.476	T	0.124	0.429	T	0.118
D	0.707	0.709	0.453	0.899	D	0.582	0.85	D	0.082
N	0.084	0.157	0.637	0.924	D	0.778	0.925	D	.
N	0.417	0.474	-1.076	0.081	T	0.003	0.008	T	.
D	0.643	0.681	-0.499	0.687	T	0.257	0.628	T	0.166

D	0.55	0.626	-1.272	0	T	0.003	0.008	T	.
D	.	.	-0.817	0.542	T	0.172	0.514	T	.
D	0.733	0.894	-1.152	0.01	T	0.068	0.281	T	0.014
D	0.336	0.435	0.105	0.843	D	0.638	0.874	D	0.048
D	0.968	0.968	0.488	0.904	D	0.652	0.879	D	.
D	0.504	0.567	0.74	0.937	D	0.829	0.943	D	0.156
N	0.249	0.311	0.538	0.911	D	0.703	0.898	D	0.042
D	0.933	0.926	0.389	0.889	D	0.786	0.928	D	0.096
D	0.151	0.185	-0.64	0.632	T	0.23	0.596	T	0.029
N	0.043	0.023	-1.081	0.07	T	0.054	0.227	T	0.009
N	0.174	0.219	-0.401	0.72	T	0.49	0.806	T	0.1
D	0.844	0.831	-0.918	0.459	T	0.001	0.002	T	.
D	0.505	0.549	-0.665	0.621	T	0.038	0.164	T	.
N	0.197	0.25	-1.142	0.013	T	0.015	0.062	T	.
D	0.128	0.166	-0.91	0.469	T	0.111	0.398	T	0.013
N	0.245	0.299	-1.014	0.255	T	0.004	0.011	T	.
D	0.492	0.548	-0.179	0.782	T	0.398	0.75	T	.
D	0.308	0.387	-0.789	0.558	T	0.088	0.341	T	0.079
D	0.508	0.693	-0.513	0.682	T	0.275	0.647	T	0.042
N	0.206	0.264	-0.987	0.333	T	0.027	0.116	T	0.094
N	0.157	0.264	-0.707	0.601	T	0.208	0.567	T	.
D	0.43	0.486	-1	0.299	T	0.001	0.004	T	.
D	0.936	0.93	-1.122	0.022	T	0.007	0.025	T	.
N	0.224	0.275	-0.807	0.548	T	0.192	0.545	T	0.007
D	0.993	0.998	0.186	0.857	D	0.302	0.673	T	.
D	0.242	0.823	-0.792	0.556	T	0.066	0.273	T	.
D	0.778	0.77	1.064	0.984	D	0.979	0.993	D	0.885
D	0.317	0.853	-1.011	0.266	T	0.022	0.095	T	0.015
D	0.577	0.618	-0.568	0.661	T	0.308	0.679	T	0.031
D	0.389	0.514	0.054	0.833	D	0.437	0.777	T	.
D	0.244	0.298	-0.972	0.368	T	0.014	0.056	T	.
D	0.552	0.588	-0.894	0.486	T	0.11	0.396	T	.
D	0.718	0.718	-0.434	0.71	T	0.216	0.578	T	.

N	0.251	0.306	-0.744	0.583	T	0.133	0.445	T	.	
N	0.301	0.361	-0.449	0.705	T	0.266	0.638	T	.	
D	0.858	0.845	0.675	0.929	D	0.669	0.885	D		0.431
N	0.085	0.138	0.605	0.92	D	0.796	0.931	D		0.43
D	0.922	0.915	1.052	0.982	D	0.874	0.958	D		0.334
D	0.807	0.812	0.136	0.848	D	0.553	0.837	D		0.075
N	0.099	0.103	-1.028	0.21	T	0.038	0.163	T	.	
N	0.528	0.568	-0.894	0.486	T	0.136	0.451	T		0.009
D	0.358	0.419	-1.118	0.024	T	0.085	0.331	T		0.031
N	0.552	0.588	-1.062	0.111	T	0.022	0.091	T		0.015
D	0.882	0.87	-1.055	0.13	T	0.002	0.005	T	.	
N	0.391	0.451	-1.052	0.138	T	0.018	0.077	T	.	
D	0.478	0.58	-0.708	0.601	T	0.354	0.718	T		0.045
D	0.527	0.567	-0.621	0.64	T	0.337	0.704	T		0.02
D	0.342	0.409	-0.659	0.623	T	0.158	0.491	T	.	
D	0.915	0.934	0.622	0.922	D	0.693	0.894	D		0.172
N	0.141	0.501	-0.55	0.668	T	0.145	0.468	T	.	
N	0.152	0.181	-1.03	0.204	T	0.011	0.042	T	.	
N	0.54	0.578	-0.654	0.625	T	0.263	0.634	T		0.046
N	0.274	0.332	-1.069	0.096	T	0.009	0.031	T	.	
D	0.296	0.356	-0.391	0.723	T	0.343	0.708	T		0.014
D	0.225	0.511	-1.228	0	T	0.015	0.061	T	.	
N	0.268	0.49	-0.461	0.701	T	0.518	0.82	D		0.037
N	0.262	0.607	-1.023	0.228	T	0.088	0.34	T		0.021
N	0.47	0.52	-0.535	0.674	T	0.279	0.651	T		0.048
N	0.381	0.441	-0.997	0.307	T	0.042	0.18	T	.	
D	0.272	0.33	-1.012	0.262	T	0.077	0.309	T		0.025
N	0.061	0.047	-1.011	0.267	T	0.054	0.23	T	.	
D	0.48	0.529	-0.696	0.607	T	0.092	0.352	T	.	
D	0.352	0.48	-0.673	0.617	T	0.25	0.62	T		0.046
N	0.068	0.059	-0.394	0.722	T	0.303	0.674	T	.	
N	0.064	0.055	-1.038	0.178	T	0.058	0.245	T		0.007
D	0.754	0.749	-1.086	0.063	T	0.026	0.112	T		0.008

D	0.678	0.698	-0.772	0.568	T	0.252	0.622	T	.
N	0.242	0.296	-0.599	0.649	T	0.131	0.441	T	.
N	0.111	0.12	-0.004	0.822	T	0.432	0.773	T	.
N	0.291	0.35	-1.1	0.041	T	0.021	0.089	T	.
N	0.103	0.107	-0.987	0.333	T	0.001	0.003	T	.
N	0.037	0.049	-0.944	0.42	T	0.001	0.003	T	.
D	0.571	0.612	-0.269	0.759	T	0.362	0.724	T	0.011
N	0.154	0.184	-0.859	0.513	T	0.161	0.497	T	0.007
N	0.169	0.265	-1.069	0.097	T	0.034	0.145	T	0.004
N	0.205	0.251	-1.038	0.18	T	0.022	0.091	T	.
N	0.223	0.274	-1.011	0.265	T	0.017	0.068	T	0.004
D	0.872	0.86	0.897	0.956	D	0.854	0.951	D	0.052
D	0.565	0.598	-0.355	0.734	T	0.249	0.619	T	.
N	0.633	0.651	-1.165	0.006	T	0.013	0.052	T	.
N	0.188	0.24	-0.623	0.639	T	0.27	0.642	T	0.064
D	0.789	0.82	-0.203	0.776	T	0.254	0.624	T	.
D	0.879	0.867	-0.307	0.748	T	0.371	0.731	T	0.143
D	0.773	0.772	-0.622	0.639	T	0.2	0.557	T	.
D	0.243	0.297	-1.013	0.26	T	0.034	0.148	T	0.006
D	0.948	0.944	-0.42	0.714	T	0.173	0.516	T	0.03
N	0.023	0.006	-1.031	0.201	T	0.019	0.078	T	.
N	0.932	0.925	-0.21	0.774	T	0.482	0.802	T	0.211
D	0.133	0.153	-0.832	0.532	T	0.142	0.464	T	.
N	0.4	0.459	-0.784	0.561	T	0.168	0.509	T	0.398
D	0.254	0.435	-0.304	0.749	T	0.325	0.694	T	0.041
N	0.13	0.149	-1.067	0.101	T	0.065	0.269	T	.
D	0.437	0.492	-0.826	0.536	T	0.051	0.218	T	.
N	0.354	0.415	-1.085	0.065	T	0.053	0.225	T	.
N	0.113	0.127	-0.907	0.472	T	0.034	0.146	T	.
N	0.193	0.236	-1.045	0.158	T	0.051	0.218	T	.
N	0.249	0.304	-1.009	0.272	T	0.059	0.247	T	0.001
N	0.305	0.365	0.642	0.924	D	0.755	0.916	D	0.276
D	0.484	0.532	0.312	0.877	D	0.637	0.873	D	0.21

D	0.453	0.506	-1.144	0.012	T	0.005	0.016	T	.
D	0.555	0.59	-1.083	0.068	T	0.049	0.208	T	.
N	0.343	0.404	-1.103	0.038	T	0.009	0.034	T	.
N	0.049	0.03	-0.982	0.345	T	0.158	0.492	T	0.773
D	0.033	0.056	-0.964	0.384	T	0	0	T	.
D	0.087	0.103	-0.905	0.474	T	0.002	0.007	T	.
N	0.153	0.182	-0.927	0.446	T	0.001	0.004	T	.
N	0.492	0.539	-1.075	0.083	T	0.041	0.177	T	0.005
N	0.159	0.191	-1.058	0.123	T	0.079	0.313	T	0.001
D	0.583	0.685	-0.693	0.608	T	0.213	0.574	T	0.083
D	0.545	0.596	0.653	0.926	D	0.774	0.923	D	0.134
D	0.77	0.763	-0.593	0.651	T	0.179	0.526	T	.
D	0.849	0.836	-0.987	0.334	T	0.044	0.19	T	.
D	0.436	0.491	-0.469	0.698	T	0.261	0.632	T	0.073
N	0.356	0.417	-1.004	0.288	T	0.05	0.212	T	.
D	0.288	0.39	-0.849	0.52	T	0.037	0.159	T	.
N	0.118	0.13	-0.912	0.466	T	0.004	0.015	T	0.02
D	0.207	0.254	-1.082	0.07	T	0.014	0.055	T	.
D	0.38	0.44	0.877	0.954	D	0.913	0.971	D	.
N	0.456	0.508	-1.014	0.256	T	0.099	0.37	T	0.018
D	0.751	0.763	1.053	0.982	D	0.941	0.981	D	0.131
N	0.384	0.697	-0.572	0.66	T	0.285	0.658	T	0.191
D	0.602	0.627	-1.045	0.157	T	0.009	0.033	T	.
D	0.821	0.817	-0.116	0.797	T	0.422	0.767	T	0.012
N	0.662	0.7	-1.072	0.089	T	0.002	0.007	T	.
N	0.57	0.612	-0.202	0.776	T	0.444	0.781	T	0.034
D	0.744	0.74	-1.022	0.232	T	0.106	0.386	T	0.027
D	0.757	0.751	-0.813	0.544	T	0.163	0.499	T	.
D	0.556	0.591	-0.117	0.797	T	0.172	0.514	T	.
D	0.845	0.832	-0.14	0.792	T	0.511	0.816	D	.

M-CAP_ranks	M-CAP_pred	CADD_raw	CADD_raw_r	CADD_phred	DANN_score	DANN_ranks	fathmm-MKI	fathmm-MKI	fathmm-MKI
.	.	5.216	0.704	25.6	0.998	0.925	0.943	0.605	D
.	.	5.327	0.721	25.8	0.999	0.936	0.988	0.871	D
.	.	3.077	0.43	22.5	0.908	0.195	0.066	0.124	N
0.323	T	1.5	0.242	13.31	0.923	0.211	0.007	0.028	N
.	.	4.443	0.595	24.2	0.971	0.319	0.231	0.217	N
.	.	6.252	0.863	28.9	0.999	0.984	0.938	0.59	D
.	.	7.067	0.94	33	0.999	0.998	0.912	0.528	D
.	.	6.963	0.935	33	0.999	0.988	0.001	0.009	N
0.71	D	5.392	0.731	26	0.997	0.81	0.978	0.769	D
.	.	-0.501	0.055	0.219	0.872	0.165	0.059	0.118	N
0.858	D	7.117	0.942	34	0.999	0.963	0.991	0.918	D
0.92	D	6.57	0.904	31	0.999	0.936	0.994	0.957	D
0.161	T	1.748	0.271	14.68	0.864	0.16	0.062	0.12	N
.	.	2.429	0.353	19.01	0.493	0.041	0.041	0.094	N
.	.	-1.373	0.02	0.004	0.302	0.016	0.035	0.085	N
0.5	D	5.996	0.824	27.8	0.992	0.548	0.988	0.874	D
0.164	T	6.333	0.875	29.3	0.999	0.977	0.916	0.537	D
.	.	4.978	0.67	25.1	0.998	0.918	0.132	0.175	N
.	.	6.378	0.881	29.5	0.999	0.972	0.955	0.649	D
.	.	3.726	0.505	23.3	0.996	0.719	0.033	0.083	N
0.271	T	1.984	0.299	16.11	0.991	0.511	0.653	0.324	D
0.337	T	5.524	0.751	26.3	0.998	0.88	0.978	0.773	D
0.884	D	4.666	0.625	24.5	0.991	0.517	0.921	0.548	D
.	.	5.02	0.676	25.2	0.998	0.925	0.745	0.362	D
.	.	4.322	0.579	24	0.988	0.455	0.406	0.262	N
0.11	T	4.233	0.567	23.9	0.999	0.982	0.959	0.664	D
.	.	3.143	0.437	22.6	0.994	0.61	0.098	0.153	N
0.098	T	3.699	0.502	23.3	0.975	0.34	0	0.001	N
.	.	4.34	0.581	24	0.992	0.554	0.956	0.652	D
.	.	-0.447	0.059	0.298	0.633	0.07	0.047	0.102	N
0.845	D	5.521	0.75	26.3	0.999	0.976	0.993	0.939	D
.	.	1.736	0.27	14.61	0.987	0.449	0.971	0.725	D

.	.	4.998	0.672	25.1	0.995	0.7	0.823	0.413	D
.	.	6.449	0.89	29.8	0.998	0.884	0.966	0.698	D
0.244	T	3.48	0.476	23.1	0.998	0.842	0.961	0.674	D
0.649	D	7.066	0.94	33	0.998	0.875	0.985	0.833	D
.	.	5.229	0.706	25.6	0.996	0.725	0.117	0.166	N
0.786	D	7.234	0.946	34	0.999	0.994	0.945	0.612	D
.	.	1.664	0.261	14.2	0.998	0.872	0.361	0.252	N
.	.	4.792	0.643	24.7	0.999	0.933	0.983	0.815	D
.	.	5.641	0.769	26.6	0.998	0.909	0.974	0.744	D
.	.	4.88	0.656	24.9	0.998	0.841	0.99	0.904	D
0.608	D	5.772	0.789	27	0.998	0.885	0.99	0.891	D
.	.	5.821	0.797	27.2	0.998	0.926	0.956	0.65	D
.	.	3.684	0.5	23.3	0.96	0.278	0.972	0.73	D
0.886	D	4.747	0.637	24.7	0.988	0.466	0.912	0.528	D
0.549	D	4.624	0.619	24.5	0.997	0.807	0.956	0.652	D
.	.	6.786	0.924	32	0.999	0.996	0.938	0.591	D
.	.	6.265	0.865	29	0.999	0.967	0.989	0.882	D
.	.	4.078	0.547	23.7	0.987	0.448	0.98	0.786	D
.	.	6.785	0.924	32	1	1	0.974	0.743	D
0.034	T	0.173	0.11	4.391	0.954	0.263	0.018	0.056	N
0.791	D	6.543	0.901	31	0.999	0.992	0.986	0.839	D
0.373	T	7.299	0.948	34	0.999	0.981	0.995	0.962	D
.	.	7.368	0.95	34	0.999	0.999	0.964	0.686	D
.	.	3.714	0.503	23.3	0.997	0.82	0.983	0.811	D
.	.	5.381	0.729	25.9	0.998	0.894	0.914	0.532	D
0.764	D	3.94	0.53	23.5	0.979	0.366	0.992	0.928	D
.	.	6.261	0.864	28.9	0.999	0.979	0.99	0.896	D
0.872	D	5.898	0.809	27.5	0.995	0.665	0.998	0.994	D
0.897	D	6.649	0.912	32	0.999	0.94	0.96	0.667	D
.	.	-0.485	0.056	0.239	0.396	0.027	0.001	0.004	N
.	.	7.101	0.942	33	0.999	0.991	0.894	0.495	D
0.012	T	0.368	0.129	6.327	0.796	0.124	0.068	0.127	N
0.011	T	-1.404	0.019	0.003	0.154	0.004	0.001	0.008	N

.	.	3.373	0.464	22.9	0.993	0.575	0.485	0.28	N
.	.	2.841	0.402	21.6	0.998	0.842	0.146	0.183	N
.	.	7.49	0.952	34	0.997	0.831	0.679	0.333	D
0.155	T	4.935	0.663	25	0.996	0.751	0.379	0.256	N
0.131	T	3.8	0.514	23.4	0.991	0.513	0.201	0.207	N
0.747	D	4.007	0.539	23.6	0.986	0.433	0.382	0.257	N
.	.	6.3	0.87	29.1	0.99	0.488	0.967	0.705	D
.	.	1.354	0.226	12.55	0.999	0.974	0.985	0.831	D
0.346	T	-0.283	0.07	0.724	0.579	0.057	0.023	0.065	N
0.243	T	4.959	0.667	25	0.998	0.897	0.934	0.579	D
.	.	0.602	0.151	8.169	0.674	0.081	0.182	0.199	N
.	.	5.43	0.736	26.1	0.991	0.517	0.93	0.567	D
.	.	5.61	0.764	26.5	0.998	0.923	0.41	0.263	N
.	.	4.42	0.592	24.2	0.995	0.682	0.994	0.952	D
0.302	T	4.578	0.613	24.4	0.991	0.51	0.446	0.271	N
0.457	T	3.902	0.526	23.5	0.995	0.671	0.951	0.633	D
0.801	D	5.372	0.728	25.9	0.999	0.931	0.675	0.332	D
0.985	D	5.622	0.766	26.6	0.995	0.698	0.933	0.575	D
0.563	D	2.201	0.326	17.51	0.927	0.217	0.978	0.767	D
.	.	5.026	0.676	25.2	0.997	0.824	0.891	0.49	D
.	.	5.336	0.722	25.8	0.999	0.955	0.989	0.888	D
.	.	5.472	0.743	26.2	0.998	0.914	0.988	0.87	D
.	.	2.85	0.403	21.6	0.993	0.6	0.08	0.138	N
.	.	6.2	0.856	28.7	0.997	0.829	0.929	0.565	D
0.36	T	5.937	0.815	27.6	0.999	0.971	0.446	0.271	N
0.489	D	3.597	0.49	23.2	0.984	0.406	0.957	0.657	D
.	.	0.75	0.166	9.153	0.788	0.12	0.602	0.309	D
0.172	T	3.291	0.454	22.8	0.993	0.582	0.404	0.262	N
.	.	5.486	0.745	26.2	0.981	0.378	0.858	0.447	D
.	.	2.933	0.413	22	0.998	0.887	0.719	0.35	D
.	.	0.709	0.162	8.889	0.449	0.034	0.032	0.08	N
0.359	T	2.265	0.333	17.94	0.98	0.368	0.889	0.488	D
.	.	4.938	0.664	25	0.997	0.824	0.969	0.711	D

.	.	6.625	0.909	32	0.999	0.993	0.889	0.488	D
0.91	D	4.157	0.557	23.8	0.987	0.446	0.937	0.586	D
.	.	0.847	0.175	9.751	0.979	0.363	0.035	0.085	N
.	.	4.516	0.605	24.3	0.998	0.854	0.762	0.37	D
.	.	4.682	0.627	24.6	0.997	0.836	0.889	0.488	D
.	.	7.454	0.951	34	0.999	0.972	0.726	0.352	D
.	.	6.129	0.845	28.4	0.998	0.928	0.963	0.681	D
.	.	7.963	0.956	35	0.999	0.99	0.843	0.43	D
.	.	2.713	0.387	20.9	0.997	0.771	0.9	0.505	D
0.263	T	1.043	0.194	10.9	0.957	0.27	0.026	0.071	N
0.855	D	5.799	0.793	27.1	0.999	0.972	0.974	0.746	D
0.607	D	4.828	0.648	24.8	0.999	0.982	0.549	0.295	D
.	.	8.001	0.957	35	0.999	0.999	0.975	0.751	D
.	.	3.653	0.496	23.2	0.998	0.875	0.359	0.252	N
.	.	0.571	0.149	7.951	0.764	0.111	0.025	0.068	N
0.756	D	6.034	0.83	28	0.996	0.723	0.936	0.584	D
.	.	2.385	0.348	18.72	0.994	0.612	0.735	0.357	D
0.282	T	1.971	0.298	16.03	0.997	0.793	0.771	0.376	D
.	.	5.03	0.677	25.2	0.999	0.962	0.948	0.623	D
0.482	D	6.423	0.887	29.7	0.995	0.705	0.948	0.622	D
0.042	T	5.324	0.72	25.8	0.995	0.703	0.946	0.615	D
.	.	6.178	0.852	28.6	0.845	0.148	0.639	0.32	D
0.26	T	3.552	0.484	23.1	0.993	0.589	0.389	0.258	N
0.406	T	7.542	0.953	34	0.997	0.802	0.91	0.524	D
.	.	0.069	0.1	3.28	0.907	0.193	0.024	0.067	N
.	.	0.322	0.125	5.908	0.884	0.174	0.14	0.18	N
0.862	D	6.677	0.914	32	0.999	0.986	0.94	0.595	D
.	.	3.479	0.476	23.1	0.964	0.291	0.962	0.677	D
0.798	D	3.007	0.422	22.3	0.908	0.195	0.015	0.049	N
0.74	D	6.199	0.856	28.7	0.999	0.99	0.958	0.661	D
.	.	5.199	0.702	25.5	0.999	0.958	0.383	0.257	N
.	.	2.444	0.355	19.1	0.997	0.82	0.218	0.213	N
0.846	D	5.506	0.748	26.3	0.999	0.976	0.95	0.629	D

.	.	7.934	0.956	35	0.999	0.999	0.951	0.633	D
.	.	1.499	0.242	13.31	0.998	0.856	0.666	0.329	D
0.337	T	7.727	0.955	35	0.999	0.997	0.975	0.749	D
0.632	D	4.561	0.611	24.4	0.991	0.517	0.87	0.462	D
.	.	6.969	0.935	33	0.997	0.836	0.947	0.617	D
0.838	D	4.989	0.671	25.1	0.999	0.962	0.839	0.426	D
0.606	D	3.898	0.525	23.5	0.993	0.597	0.936	0.585	D
0.766	D	6.567	0.903	31	0.999	0.937	0.975	0.747	D
0.517	D	4.351	0.582	24.1	0.998	0.867	0.989	0.877	D
0.244	T	1.642	0.259	14.08	0.987	0.438	0.886	0.483	D
0.773	D	2.282	0.335	18.05	0.921	0.208	0.256	0.225	N
.	.	6.345	0.876	29.3	0.999	0.994	0.982	0.802	D
.	.	3.684	0.5	23.3	0.989	0.471	0.412	0.264	N
.	.	5.803	0.794	27.1	0.996	0.735	0.217	0.213	N
0.33	T	2.852	0.404	21.6	0.999	0.967	0.085	0.143	N
.	.	0.534	0.145	7.686	0.82	0.135	0.285	0.234	N
.	.	6.309	0.871	29.2	0.999	0.949	0.735	0.357	D
0.732	D	5.03	0.677	25.2	0.999	0.936	0.543	0.293	D
0.603	D	7.593	0.953	34	0.999	0.997	0.886	0.484	D
0.762	D	5.591	0.761	26.5	0.995	0.655	0.13	0.174	N
.	.	3.074	0.429	22.5	0.981	0.379	0.541	0.293	D
.	.	4.67	0.626	24.5	0.998	0.919	0.457	0.274	N
.	.	5.872	0.805	27.4	0.999	0.942	0.99	0.904	D
0.186	T	6.557	0.902	31	0.997	0.786	0.651	0.324	D
.	.	7.229	0.946	34	0.999	0.995	0.988	0.871	D
.	.	6.771	0.922	32	0.999	0.986	0.987	0.856	D
0.991	D	6.104	0.841	28.3	0.993	0.584	0.96	0.671	D
0.356	T	5.232	0.707	25.6	0.999	0.995	0.994	0.952	D
0.537	D	6.492	0.895	30	0.998	0.847	0.947	0.618	D
.	.	6.748	0.921	32	0.999	0.971	0.999	1	D
.	.	3.586	0.488	23.2	0.998	0.883	0.876	0.469	D
.	.	4.033	0.542	23.7	0.996	0.764	0.974	0.742	D
.	.	6.375	0.88	29.5	0.999	0.986	0.991	0.908	D

.	.	3.903	0.526	23.5	0.997	0.804	0.908	0.519	D
.	.	6.243	0.862	28.9	0.996	0.729	0.945	0.61	D
0.94	D	6.326	0.874	29.3	0.999	0.979	0.878	0.472	D
0.94	D	5.429	0.736	26.1	0.997	0.817	0.987	0.854	D
0.919	D	6.417	0.886	29.7	0.997	0.812	0.969	0.716	D
0.724	D	4.94	0.664	25	0.998	0.902	0.968	0.705	D
.	.	0.008	0.094	2.661	0.655	0.076	0.518	0.287	D
0.247	T	3.412	0.468	23	0.998	0.889	0.792	0.389	D
0.533	D	6.213	0.857	28.7	0.999	0.964	0.904	0.512	D
0.354	T	5.253	0.71	25.6	0.987	0.444	0.608	0.31	D
.	.	4.857	0.652	24.9	0.995	0.685	0.618	0.313	D
.	.	1.371	0.228	12.64	0.938	0.232	0.133	0.176	N
0.621	D	3.469	0.475	23	0.995	0.66	0.606	0.31	D
0.431	T	6.327	0.874	29.3	0.992	0.542	0.95	0.628	D
.	.	5.518	0.75	26.3	0.999	0.94	0.693	0.339	D
0.85	D	4.61	0.618	24.4	0.999	0.99	0.991	0.908	D
.	.	1.862	0.285	15.36	0.997	0.82	0.927	0.561	D
.	.	3.237	0.448	22.8	0.985	0.42	0.081	0.139	N
0.623	D	4.436	0.594	24.2	0.998	0.891	0.979	0.777	D
.	.	6.268	0.865	29	0.993	0.581	0.645	0.321	D
0.335	T	3.278	0.453	22.8	0.997	0.798	0.398	0.261	N
.	.	5.295	0.716	25.7	0.999	0.936	0.887	0.485	D
0.572	D	2.309	0.339	18.22	0.995	0.701	0.087	0.144	N
0.444	T	2.643	0.379	20.5	0.978	0.356	0.665	0.328	D
0.632	D	5.103	0.688	25.3	0.997	0.838	0.848	0.436	D
.	.	4.427	0.593	24.2	0.993	0.568	0.957	0.655	D
0.483	D	4.143	0.556	23.8	0.995	0.669	0.939	0.591	D
.	.	1.644	0.259	14.09	0.981	0.374	0.057	0.114	N
.	.	5.857	0.803	27.3	0.999	0.973	0.954	0.645	D
0.624	D	2.107	0.314	16.9	0.996	0.754	0.996	0.975	D
.	.	0.667	0.158	8.613	0.645	0.073	0.836	0.424	D
0.2	T	0.095	0.103	3.556	0.877	0.169	0.028	0.075	N
0.216	T	4.556	0.61	24.4	0.991	0.521	0.962	0.679	D

.	.	5.411	0.733	26	0.999	0.943	0.428	0.267	N
.	.	2.948	0.415	22	0.997	0.787	0.978	0.769	D
.	.	3.346	0.461	22.9	0.997	0.806	0.314	0.241	N
.	.	4.609	0.617	24.4	0.99	0.508	0.921	0.547	D
.	.	2.46	0.357	19.21	0.934	0.226	0.103	0.157	N
.	.	1.459	0.238	13.09	0.719	0.095	0.452	0.272	N
0.285	T	5.199	0.702	25.5	0.963	0.287	0.934	0.578	D
0.198	T	2.883	0.407	21.8	0.899	0.187	0.681	0.334	D
0.105	T	3.214	0.446	22.7	0.968	0.307	0.183	0.2	N
.	.	1.803	0.278	15.01	0.271	0.013	0.149	0.184	N
0.104	T	1.335	0.224	12.45	0.97	0.316	0.042	0.096	N
0.65	D	6.419	0.886	29.7	0.999	0.978	0.991	0.917	D
.	.	6.481	0.894	30	0.999	0.982	0.288	0.234	N
.	.	4.726	0.634	24.6	0.995	0.654	0.983	0.812	D
0.694	D	2.462	0.357	19.23	0.995	0.665	0.459	0.274	N
.	.	6.423	0.887	29.7	0.933	0.224	0.935	0.58	D
0.826	D	6.589	0.906	31	0.999	0.969	0.993	0.942	D
.	.	7.903	0.956	35	0.999	0.999	0.991	0.907	D
0.154	T	0.649	0.156	8.491	0.72	0.095	0.582	0.303	D
0.522	D	5.497	0.747	26.2	0.996	0.722	0.989	0.885	D
.	.	1.193	0.209	11.71	0.909	0.196	0.1	0.155	N
0.873	D	6.617	0.909	32	0.998	0.911	0.984	0.822	D
.	.	3.441	0.472	23	0.997	0.806	0.673	0.331	D
0.933	D	-0.979	0.033	0.018	0.657	0.076	0.452	0.272	N
0.596	D	6.607	0.908	32	0.918	0.205	0.931	0.57	D
.	.	3.474	0.475	23	0.992	0.548	0.659	0.326	D
.	.	5.223	0.705	25.6	0.997	0.84	0.277	0.231	N
.	.	5.303	0.717	25.8	0.996	0.745	0.506	0.285	D
.	.	-0.031	0.091	2.297	0.902	0.189	0.003	0.015	N
.	.	4.135	0.555	23.8	0.993	0.58	0.813	0.405	D
0.002	T	-0.208	0.075	1.054	0.286	0.015	0.002	0.014	N
0.901	D	6.341	0.876	29.3	0.992	0.54	0.655	0.325	D
0.873	D	5.181	0.699	25.5	0.997	0.839	0.929	0.565	D

.	.	3.345	0.461	22.9	0.994	0.647	0.47	0.276	N
.	.	3.398	0.467	23	0.983	0.393	0.187	0.202	N
.	.	4.11	0.551	23.7	0.996	0.721	0.922	0.55	D
0.982	D	1.166	0.207	11.57	0.995	0.693	0.1	0.155	N
.	.	0.493	0.141	7.377	0.996	0.722	0.142	0.181	N
.	.	1.568	0.25	13.68	0.964	0.291	0.011	0.039	N
.	.	-0.37	0.064	0.457	0.738	0.101	0.078	0.137	N
0.122	T	4.397	0.589	24.1	0.991	0.517	0	0.002	N
0.015	T	0.263	0.119	5.327	0.996	0.766	0.051	0.108	N
0.74	D	6.647	0.912	32	0.999	0.988	0.965	0.691	D
0.817	D	5.596	0.762	26.5	0.999	0.979	0.953	0.639	D
.	.	8.066	0.957	35	0.999	0.99	0.971	0.725	D
.	.	6.363	0.879	29.4	1	1	0.949	0.625	D
0.717	D	4.391	0.588	24.1	0.996	0.744	0.988	0.863	D
.	.	7.084	0.941	33	0.999	0.994	0.154	0.187	N
.	.	3.298	0.455	22.9	0.995	0.703	0.196	0.205	N
0.423	T	1.134	0.203	11.4	0.752	0.106	0.409	0.263	N
.	.	4.259	0.571	23.9	0.995	0.676	0.092	0.148	N
.	.	3.893	0.525	23.5	0.998	0.892	0.762	0.371	D
0.401	T	4.482	0.6	24.2	0.997	0.828	0.811	0.403	D
0.814	D	2.591	0.373	20.1	0.993	0.578	0.966	0.697	D
0.862	D	4.263	0.571	23.9	0.989	0.477	0.843	0.431	D
.	.	7.331	0.949	34	0.999	0.976	0.6	0.308	D
0.297	T	7.761	0.955	35	0.999	0.99	0.889	0.488	D
.	.	6.111	0.842	28.3	0.995	0.682	0.864	0.454	D
0.552	D	4.659	0.624	24.5	0.997	0.825	0.972	0.73	D
0.501	D	5.537	0.753	26.3	0.998	0.928	0.958	0.661	D
.	.	5.246	0.709	25.6	0.995	0.69	0.926	0.558	D
.	.	2.622	0.376	20.3	0.972	0.325	0.891	0.491	D
.	.	5.826	0.798	27.2	0.998	0.899	0.844	0.432	D

Eigen_coding	Eigen-raw	Eigen-PC-raw	GenoCanyon_	GenoCanyon_	integrated_fi	integrated_fi	integrated_c	GERP++_RS	GERP++_RS_
c	0.249	0.187	1	0.473	0.706	0.609	0	3.33	0.371
c	0.711	0.6	1	0.747	0.428	0.059	0	5.19	0.713
c	-0.674	-0.93	1	0.517	0.65	0.456	0	.	.
c	-1.148	-1.296	1	0.517	0.65	0.456	0	.	.
c	0.501	0.221	1	0.747	0.206	0.033	2	2.18	0.267
c	0.31	0.309	0.993	0.332	0.635	0.413	0	3.91	0.442
c	0.748	0.725	1	0.747	0.752	0.988	0	5.26	0.734
c	0.472	0.414	1	0.747	0.563	0.31	0	3.13	0.349
c	0.767	0.728	1	0.747	0.672	0.522	0	4.94	0.645
c	-1.013	-1.075	1	0.408	0.549	0.223	0	0.712	0.172
c	0.964	0.931	1	0.983	0.554	0.246	0	5.63	0.861
c	0.8	0.728	1	0.747	0.487	0.133	0	6.07	0.987
c	-1.027	-1.292	1	0.424	0.487	0.133	0	-9.31	0.005
c	-0.49	-0.636	0	0.035	0.487	0.133	0	2.17	0.266
c	-1.427	-1.516	1	0.473	0.706	0.609	0	-3.71	0.041
c	0.652	0.643	1	0.747	0.563	0.31	0	4.64	0.572
c	0.674	0.671	1	0.747	0.707	0.73	0	5.5	0.813
c	-0.024	-0.089	0.031	0.14	0.487	0.133	0	3.92	0.444
c	0.632	0.624	1	0.747	0.487	0.133	0	5.08	0.682
c	-0.314	-0.44	0.355	0.197	0.653	0.482	0	3.11	0.347
c	-0.389	-0.304	1	0.48	0.497	0.185	0	1.2	0.201
c	0.559	0.572	0.997	0.352	0.707	0.73	0	5.32	0.753
c	0.096	-0.052	0.3	0.192	0.563	0.31	0	-3.3	0.047
c	0.408	0.221	0.684	0.225	0.447	0.083	0	4.04	0.461
c	0.039	-0.177	0	0.052	0.554	0.283	0	-0.316	0.121
c	0.451	0.52	1	0.517	0.732	0.924	0	5.52	0.821
c	-0.883	-0.86	1	0.48	0.628	0.401	0	1.58	0.224
c	-0.784	-1.048	0.001	0.085	0.563	0.31	0	-0.595	0.11
c	0.436	0.216	0	0.069	0.487	0.133	0	3.07	0.343
c	-1.227	-1.294	0	0.012	0.554	0.246	0	0.449	0.158
c	0.886	0.818	1	0.983	0.707	0.73	0	5.36	0.765
c	0.134	0.213	1	0.747	0.563	0.31	0	3.86	0.435

c	0.469	0.453	1	0.747	0.568	0.321	0	4.76	0.6
c	0.738	0.707	1	0.747	0.722	0.854	0	5.49	0.809
c	-0.016	0.186	1	0.747	0.707	0.73	0	5.46	0.799
c	0.927	0.857	1	0.747	0.706	0.609	0	4.87	0.627
c	-0.041	-0.219	1	0.747	0.443	0.08	1	4.06	0.464
c	0.501	0.429	0.001	0.074	0.581	0.33	0	2.86	0.323
c	-0.263	-0.242	0.165	0.177	0.707	0.73	0	3.25	0.362
c	0.772	0.742	1	0.747	0.487	0.133	0	5.97	0.969
c	0.609	0.562	0.137	0.172	0.487	0.133	0	4.87	0.627
c	0.52	0.608	1	0.747	0.615	0.372	0	5.8	0.921
c	0.458	0.529	1	0.747	0.707	0.73	0	5.85	0.936
c	0.949	0.902	1	0.747	0.732	0.924	0	6.07	0.987
c	0.848	0.831	1	0.747	0.554	0.246	0	5.96	0.967
c	0.152	0.097	0.856	0.251	0.732	0.924	0	3.55	0.396
c	0.388	0.466	1	0.747	0.707	0.73	0	5.8	0.921
c	0.621	0.493	0.622	0.219	0.487	0.133	0	4.13	0.475
c	0.899	0.919	1	0.747	0.732	0.924	0	6.06	0.983
c	0.331	0.387	1	0.747	0.707	0.73	0	5.22	0.722
c	0.573	0.526	1	0.489	0.737	0.974	0	4.16	0.48
c	-0.714	-1.021	0.658	0.222	0.498	0.195	0	-0.565	0.111
c	0.797	0.76	1	0.983	0.66	0.49	0	4.77	0.603
c	0.927	0.89	1	0.747	0.707	0.73	0	5.62	0.857
c	0.915	0.88	1	0.983	0.722	0.854	0	5.47	0.803
c	0.638	0.627	1	0.747	0.732	0.924	0	5.68	0.88
c	0.161	0.355	0.95	0.278	0.487	0.133	0	6.16	0.993
c	0.2	0.348	1	0.747	0.706	0.609	0	5.51	0.817
c	0.875	0.84	1	0.747	0.615	0.372	0	5.74	0.9
c	1.057	0.991	1	0.747	0.516	0.203	0	5.55	0.832
c	0.532	0.457	0.995	0.339	0.516	0.203	0	3.39	0.378
c	-1.963	-2.017	0.614	0.218	0.497	0.185	0	-6.31	0.018
c	0.513	0.481	0.081	0.159	0.635	0.413	0	4.81	0.613
c	-1.022	-1.255	1	0.48	0.005	0	3	-2.15	0.067
c	-1.741	-1.815	0.001	0.08	0.66	0.495	0	.	.

c	-0.044	-0.114	0.986	0.31	0.554	0.283	0	2.97	0.333
c	-0.339	-0.588	1	0.434	0.583	0.331	0	1.99	0.253
c	0.543	0.461	1	0.747	0.442	0.072	0	3.8	0.427
c	-0.678	-0.907	0.942	0.275	0.516	0.203	0	-4.71	0.03
c	-0.502	-0.572	0.985	0.308	0.707	0.73	0	0.011	0.133
c	-0.175	-0.046	1	0.747	0.442	0.072	0	4.61	0.566
c	0.528	0.513	0.997	0.349	0.616	0.391	0	4.84	0.62
c	0.719	0.749	1	0.747	0.706	0.609	0	5.8	0.921
c	-1.116	-1.201	1	0.473	0.652	0.477	0	0.155	0.141
c	0.64	0.53	1	0.747	0.672	0.522	0	4.81	0.613
c	-1.176	-1.145	0.915	0.264	0.554	0.283	0	1.9	0.247
c	0.709	0.708	1	0.747	0.741	0.977	0	5.24	0.728
c	-0.11	-0.026	1	0.747	0.442	0.072	0	3.69	0.413
c	0.831	0.682	1	0.462	0.651	0.465	0	4.78	0.605
c	0.043	-0.121	0.264	0.188	0.549	0.223	0	3.52	0.393
c	-0.126	-0.044	1	0.489	0.707	0.73	0	4.35	0.513
c	0.515	0.4	1	0.747	0.549	0.223	0	5.44	0.793
c	0.429	0.387	1	0.747	0.598	0.34	0	4.12	0.473
c	0.092	0.025	1	0.489	0.701	0.575	0	4.28	0.5
c	0.32	0.422	1	0.747	0.732	0.924	0	5.75	0.904
c	0.691	0.569	1	0.747	0.672	0.522	0	3.92	0.444
c	0.703	0.661	1	0.747	0.615	0.372	0	4.63	0.57
c	-0.092	-0.381	0	0.054	0.487	0.133	0	2.54	0.295
c	0.481	0.436	1	0.747	0.707	0.73	0	4.51	0.544
c	-0.136	-0.219	0.999	0.397	0.707	0.73	0	2.94	0.33
c	0.448	0.267	1	0.747	0.615	0.372	0	4.26	0.497
c	-0.443	-0.432	1	0.747	0.646	0.45	0	4.5	0.542
c	-0.876	-0.795	1	0.747	0.628	0.401	0	2.27	0.274
c	-0.159	-0.122	1	0.983	0.442	0.072	0	4.9	0.635
c	0.083	-0.092	0.051	0.149	0.554	0.246	0	2.55	0.296
c	-1.413	-1.588	1	0.747	0.554	0.283	0	-9.71	0.004
c	-0.61	-0.578	0.004	0.105	0.554	0.283	0	3.58	0.4
c	0.564	0.571	1	0.48	0.732	0.924	0	5.38	0.772

c	0.553	0.506	1	0.747	0.615	0.372	0	5.58	0.843
c	0.454	0.443	1	0.747	0.701	0.575	0	5.21	0.719
c	-0.636	-0.657	0.007	0.113	0.615	0.372	0	1.17	0.199
c	0.368	0.286	1	0.747	0.487	0.133	0	3.49	0.389
c	0.458	0.359	0.058	0.151	0.732	0.924	0	3.41	0.38
c	0.459	0.243	1	0.422	0.526	0.212	0	-1.92	0.072
c	0.704	0.606	1	0.983	0.554	0.283	0	5.85	0.936
c	0.767	0.75	1	0.747	0.421	0.056	0	5.66	0.872
c	0.055	0.079	0.998	0.358	0.497	0.185	0	5.01	0.663
c	-1.151	-1.12	0.001	0.083	0.554	0.246	0	3.29	0.366
c	0.533	0.456	0.997	0.348	0.497	0.185	0	3.86	0.435
c	0.456	0.367	0	0.012	0.487	0.133	0	3.46	0.386
c	0.776	0.739	1	0.983	0.731	0.878	0	4.86	0.625
c	-0.12	-0.135	1	0.747	0.497	0.185	0	5.13	0.696
c	-0.675	-0.893	1	0.747	0.487	0.133	0	0.46	0.158
c	0.874	0.821	1	0.747	0.554	0.283	0	6.03	0.978
c	0.047	0.176	0.776	0.237	0.707	0.73	0	4.47	0.536
c	-0.11	-0.127	0.002	0.087	0.554	0.246	0	2.04	0.257
c	0.535	0.497	0.003	0.095	0.487	0.133	0	4.17	0.481
c	0.667	0.655	0.837	0.248	0.447	0.083	0	5.7	0.886
c	-0.018	-0.114	1	0.747	0.732	0.924	0	2.47	0.29
c	0.227	0.198	1	0.747	0.564	0.319	0	4.89	0.632
c	-0.364	-0.345	1	0.432	0.554	0.246	0	1.01	0.19
c	0.448	0.521	1	0.747	0.732	0.924	0	5.3	0.746
c	-1.293	-1.432	1	0.747	0.516	0.203	0	-1.14	0.092
c	-0.443	-0.698	1	0.747	0.616	0.392	0	0.698	0.172
c	0.666	0.645	1	0.747	0.563	0.31	0	5.52	0.821
c	0.032	0.047	1	0.747	0.578	0.323	2	2.18	0.267
c	-1.149	-1.399	1	0.747	0.742	0.98	0	-6.35	0.018
c	0.459	0.411	1	0.747	0.66	0.495	0	4.62	0.568
c	-0.227	-0.384	0.542	0.212	0.516	0.203	0	-3.04	0.051
c	-0.864	-0.827	0.02	0.132	0.706	0.609	0	3.65	0.408
c	0.65	0.553	1	0.747	0.707	0.73	0	5.29	0.743

c	0.729	0.714	1	0.408	0.732	0.924	0	5.8	0.921
c	0.705	0.682	0.003	0.097	0.615	0.372	0	4.9	0.635
c	0.858	0.846	1	0.983	0.632	0.408	0	5.36	0.765
c	0.199	0.279	1	0.747	0.554	0.283	0	4.85	0.622
c	0.766	0.685	1	0.747	0.447	0.083	0	4.75	0.598
c	0.567	0.54	1	0.454	0.515	0.199	0	5.07	0.68
c	0.23	0.219	1	0.747	0.497	0.185	0	5.61	0.853
c	-0.018	0.13	0.992	0.325	0.497	0.185	0	5.4	0.779
c	0.347	0.291	0.553	0.213	0.554	0.283	0	1.94	0.249
c	-0.393	-0.406	1	0.431	0.635	0.413	0	3.18	0.355
c	-0.452	-0.462	0.999	0.378	0.598	0.34	0	2.25	0.272
c	0.961	0.86	1	0.983	0.487	0.133	0	5.48	0.806
c	0.223	0.154	0.999	0.377	0.554	0.246	0	5.3	0.746
c	0.168	0.09	0.991	0.324	0.638	0.428	0	3.74	0.419
c	-0.669	-0.725	0.846	0.249	0.549	0.223	0	1.83	0.242
c	-1.296	-1.259	0.975	0.295	0.672	0.522	0	-0.331	0.12
c	0.228	0.077	0.415	0.202	0.706	0.609	0	1.85	0.243
c	0.212	0.295	1	0.429	0.66	0.495	0	5.23	0.725
c	0.627	0.591	0.721	0.229	0.732	0.924	0	4.64	0.572
c	-0.349	-0.449	1	0.747	0.623	0.393	0	1.84	0.242
c	-0.209	-0.176	1	0.747	0.524	0.211	1	1.29	0.206
c	-0.452	-0.423	0.043	0.146	0.487	0.133	0	4.29	0.502
c	0.891	0.822	1	0.747	0.615	0.352	0	5.48	0.806
c	-0.033	0.062	1	0.747	0.726	0.872	0	3.96	0.449
c	0.689	0.641	1	0.747	0.635	0.413	0	5.19	0.713
c	0.687	0.63	1	0.747	0.487	0.133	0	5.4	0.779
c	0.987	0.898	1	0.747	0.461	0.09	0	4.89	0.632
c	0.453	0.541	1	0.747	0.626	0.397	0	5.61	0.853
c	0.353	0.436	1	0.437	0.635	0.413	0	5.59	0.846
c	0.89	0.839	1	0.747	0.707	0.73	0	5.27	0.737
c	0.305	0.295	1	0.747	0.554	0.283	0	3.82	0.43
c	0.442	0.522	0.939	0.274	0.732	0.924	0	5.64	0.864
c	0.705	0.662	1	0.747	0.497	0.185	0	5.32	0.753

c	0.087	0.292	1	0.517	0.707	0.73	0	5.77	0.91
c	0.536	0.469	0.119	0.168	0.638	0.428	0	4.31	0.505
c	0.78	0.632	1	0.983	0.437	0.062	0	3.8	0.427
c	0.15	0.122	1	0.747	0.707	0.73	0	5.37	0.769
c	0.977	0.906	1	0.747	0.672	0.522	0	5.75	0.904
c	0.788	0.72	1	0.747	0.722	0.854	0	5.58	0.843
c	-0.525	-0.572	1	0.425	0.616	0.391	0	1.84	0.242
c	-0.031	-0.093	0.963	0.286	0.707	0.73	0	-2.6	0.058
c	0.222	0.176	1	0.443	0.707	0.73	0	2.57	0.298
c	0.157	0.114	1	0.747	0.635	0.413	0	4.55	0.553
c	-0.581	-0.639	0.145	0.173	0.615	0.372	0	-2.73	0.056
c	-0.468	-0.597	0.726	0.23	0.615	0.372	0	2.33	0.279
c	0.228	0.122	0.784	0.239	0.447	0.083	0	3.43	0.382
c	0.686	0.687	1	0.747	0.732	0.924	0	5.67	0.876
c	-0.004	0.037	1	0.747	0.554	0.283	0	4.85	0.622
c	0.982	0.923	1	0.747	0.719	0.83	0	5.96	0.967
c	-0.016	0.094	0.998	0.364	0.638	0.428	0	4.98	0.655
c	-1.192	-1.268	1	0.747	0.455	0.088	0	-1.34	0.086
c	0.349	0.441	1	0.747	0.713	0.817	0	5.71	0.89
c	-0.255	-0.142	1	0.489	0.487	0.133	0	4.46	0.534
c	0.196	0.104	0.932	0.27	0.497	0.185	0	3.53	0.394
c	0.182	0.073	0.472	0.207	0.635	0.413	0	-0.536	0.112
c	-0.126	-0.194	0	0.063	0.116	0.024	0	3	0.336
c	-0.369	-0.365	0.963	0.286	0.707	0.73	0	3.63	0.406
c	0.01	0.197	0.952	0.279	0.707	0.73	0	5.43	0.789
c	0.193	0.218	1	0.747	0.744	0.983	0	4.76	0.6
c	0.032	-0.009	1	0.747	0.707	0.73	0	5.34	0.759
c	-1.057	-1.24	1	0.747	0.442	0.072	0	-3.48	0.044
c	0.514	0.453	0.993	0.331	0.707	0.73	0	4.85	0.622
c	0.022	0.135	1	0.747	0.635	0.413	0	5.01	0.663
c	-0.311	-0.274	1	0.517	0.672	0.522	0	3.59	0.401
c	-0.914	-1.054	0.056	0.151	0.706	0.609	0	-2.02	0.069
c	0.21	0.283	0.999	0.384	0.578	0.323	0	5.18	0.71

c	0.502	0.373	1	0.417	0.496	0.176	0	3.37	0.375
c	0.427	0.429	1	0.747	0.372	0.05	0	5.11	0.691
c	-0.333	-0.206	1	0.5	0.672	0.522	0	4.85	0.622
c	0.058	0.1	0.964	0.287	0.672	0.522	0	5.11	0.691
c	-0.769	-0.707	0.187	0.18	0.706	0.609	0	1.99	0.253
c	-0.894	-0.769	0.066	0.154	0.672	0.522	0	0.151	0.141
c	0.693	0.656	1	0.747	0.722	0.854	0	4.41	0.524
c	0.026	0.023	0.035	0.142	0.732	0.924	0	3.75	0.421
c	-0.476	-0.654	1	0.747	0.732	0.924	0	0.889	0.183
c	-0.747	-0.781	1	0.747	0.598	0.34	0	3.26	0.363
c	-0.909	-0.968	0	0.05	0.516	0.203	0	1.17	0.199
c	0.872	0.812	1	0.747	0.554	0.283	0	4.72	0.591
c	0.27	0.196	0.972	0.293	0.515	0.199	0	4.46	0.534
c	0.415	0.372	0.775	0.237	0.672	0.522	0	5.22	0.722
c	-0.692	-0.719	0.942	0.275	0.696	0.567	0	1.69	0.232
c	0.463	0.452	1	0.747	0.554	0.283	0	4.26	0.497
c	0.77	0.776	1	0.747	0.731	0.878	0	6.06	0.983
c	0.717	0.653	1	0.747	0.554	0.246	0	4.88	0.63
c	-0.922	-0.913	0.144	0.173	0.732	0.924	0	3.29	0.366
c	0.958	0.864	1	0.983	0.497	0.185	0	5.01	0.663
c	-1.13	-1.047	0.089	0.161	0.487	0.133	0	-1.99	0.07
c	0.488	0.439	1	0.747	0.707	0.73	0	4.72	0.591
c	0.264	0.221	0.018	0.131	0.5	0.196	0	2.78	0.316
c	-0.34	-0.333	0.996	0.343	0.598	0.34	0	2.82	0.319
c	0.649	0.639	0.001	0.076	0.554	0.246	0	4.68	0.582
c	-0.591	-0.5	1	0.747	0.658	0.486	0	1.93	0.249
c	0.203	0.059	0	0.065	0.516	0.203	0	2.79	0.317
c	0.006	0.031	1	0.747	0.66	0.495	0	4.9	0.635
c	-1.442	-1.543	0	0.012	0.549	0.223	0	-0.635	0.109
c	-0.118	0.064	1	0.747	0.652	0.477	0	4.95	0.648
c	-0.645	-0.894	0	0.05	0.487	0.133	0	-0.773	0.104
c	0.404	0.318	1	0.747	0.635	0.413	0	4.44	0.53
c	0.172	0.113	0.863	0.252	0.732	0.924	0	3.52	0.393

c	-0.073	-0.223	0.344	0.196	0.706	0.609	0	3.09	0.345
c	0.041	-0.295	0	0.035	0.615	0.372	0	1.3	0.207
c	0.309	0.266	0.893	0.259	0.646	0.45	0	4.38	0.519
c	-0.921	-0.869	0.987	0.312	0.581	0.326	0	-0.43	0.116
c	-0.899	-1.001	0	0.068	0.707	0.73	0	1.63	0.228
c	-1.109	-1.243	0	0.012	0.707	0.73	0	-0.817	0.102
c	-1.067	-1.14	1	0.458	0.66	0.495	0	-0.748	0.105
c	-0.43	-0.369	0.007	0.115	0.707	0.73	0	2.99	0.335
c	-0.589	-0.754	0.004	0.105	0.653	0.482	0	0.842	0.18
c	0.573	0.51	1	0.423	0.66	0.495	0	4.86	0.625
c	0.595	0.566	1	0.747	0.722	0.854	0	4.55	0.553
c	0.82	0.799	1	0.983	0.707	0.73	0	5.16	0.704
c	0.794	0.765	1	0.983	0.707	0.73	0	5.54	0.828
c	0.599	0.607	1	0.747	0.696	0.567	0	4.56	0.555
c	0.257	0.19	0.89	0.258	0.646	0.45	0	3.32	0.37
c	-0.198	-0.307	0.997	0.349	0.635	0.413	0	4.63	0.57
c	-1.015	-0.994	0.114	0.167	0.707	0.73	0	1.75	0.236
c	-0.627	-0.57	0.988	0.314	0.615	0.372	0	4.65	0.575
c	0.569	0.381	1	0.747	0.66	0.495	0	4.54	0.551
c	0.37	0.388	1	0.451	0.635	0.413	0	4.71	0.589
c	0.787	0.779	1	0.747	0.672	0.522	0	5.7	0.886
c	0.18	0.208	1	0.747	0.652	0.477	0	3.77	0.423
c	0.104	0.094	0.829	0.246	0.706	0.609	0	2.41	0.285
c	0.664	0.633	0.061	0.152	0.706	0.609	0	4.58	0.559
c	0.2	0.302	0.372	0.199	0.732	0.924	0	4.15	0.478
c	0.773	0.773	1	0.517	0.732	0.924	0	5.93	0.959
c	0.499	0.382	1	0.419	0.672	0.522	0	3.94	0.446
.	.	.	1	0.983	.	.	.	3.36	0.374
.	.	.	1	0.747	.	.	.	3.47	0.387
.	.	.	1	0.747	.	.	.	3.47	0.387

phyloP100wa	phyloP100wa	phyloP20way	phyloP20way	phastCons10	phastCons10	phastCons20	phastCons20	SiPhy_29way	SiPhy_29way
3.498	0.528	0.994	0.605	1	0.715	0.599	0.288	10.408	0.433
5.985	0.7	0.852	0.362	1	0.715	0.223	0.222	17.725	0.882
-0.049	0.118	0	0.135	0.001	0.137	0.162	0.208	4.66	0.119
-0.536	0.062	0	0.135	0.003	0.159	0.173	0.211	.	.
0.701	0.251	0.875	0.382	0.047	0.211	0.987	0.523	6.478	0.211
1.183	0.314	0.953	0.551	0.818	0.297	1	0.888	8.775	0.337
6.75	0.745	0.953	0.551	1	0.715	0.999	0.75	17.429	0.874
6.594	0.738	0.998	0.613	1	0.715	0.555	0.28	12.486	0.55
9.507	0.97	1.048	0.713	1	0.715	0.971	0.463	18.42	0.905
0.544	0.228	-0.36	0.068	0.697	0.284	0.003	0.074	4.732	0.123
10.003	0.997	1.048	0.713	1	0.715	0.994	0.587	19.281	0.94
7.867	0.854	0.892	0.403	1	0.715	0.507	0.272	20.652	0.996
-0.081	0.112	0.046	0.159	0	0.063	0.006	0.095	5.425	0.156
1.897	0.392	0.953	0.551	0.01	0.182	0.002	0.062	5.158	0.143
-1.554	0.023	-0.139	0.11	0	0.063	0.002	0.062	9.853	0.4
9.225	0.943	1.073	0.849	1	0.715	0.999	0.75	13.046	0.582
2.792	0.473	0.935	0.49	0.991	0.37	0.033	0.15	17.253	0.869
-0.429	0.07	0.789	0.32	0	0.063	0.006	0.095	9.104	0.357
4.591	0.605	0.935	0.49	1	0.715	1	0.888	18.056	0.892
1.863	0.389	1.048	0.713	0.009	0.18	0.03	0.147	6.035	0.188
0.25	0.179	0.935	0.49	0.988	0.363	1	0.888	0.49	0.005
3.593	0.535	0.935	0.49	1	0.715	0.992	0.562	13.015	0.58
0.372	0.201	-0.139	0.11	0.995	0.385	0.995	0.604	14.476	0.67
3.398	0.521	0.892	0.403	0.935	0.322	0.052	0.166	12.016	0.524
0.749	0.258	-0.011	0.132	0.146	0.234	0.868	0.363	9.511	0.381
5.513	0.665	0.932	0.445	1	0.715	1	0.888	19.442	0.948
0.926	0.282	0.935	0.49	0.001	0.137	0.177	0.212	3.157	0.061
0.395	0.204	0.242	0.267	0.129	0.231	0.037	0.154	5.028	0.136
5.291	0.652	0.989	0.601	0.999	0.424	0.01	0.111	7.913	0.288
-0.049	0.118	-1.455	0.011	0	0.063	0.04	0.157	8.885	0.344
9.778	0.982	1.045	0.669	1	0.715	0.017	0.128	19.515	0.951
5.284	0.652	0.852	0.362	1	0.715	1	0.888	14.812	0.695

2.752	0.469	0.935	0.49	1	0.715	0.998	0.697	14.788	0.693
3.556	0.532	0.935	0.49	1	0.715	0.912	0.389	16.513	0.841
4.591	0.605	1.048	0.713	1	0.715	0.999	0.75	13.626	0.615
9.994	0.993	1.048	0.713	1	0.715	0.974	0.471	15.89	0.79
2.116	0.413	0.935	0.49	0.038	0.207	0.001	0.043	9.055	0.354
3.097	0.497	0.038	0.153	1	0.715	0.97	0.461	14.259	0.655
0.459	0.215	0.852	0.362	0.132	0.232	0.652	0.299	8.407	0.316
6.1	0.709	1.061	0.807	1	0.715	1	0.888	16.461	0.838
3.433	0.523	1.011	0.635	1	0.715	1	0.888	12.747	0.565
9.325	0.96	1.199	0.96	1	0.715	0.999	0.75	16.148	0.813
8.586	0.905	1.14	0.893	1	0.715	0.994	0.587	16.236	0.821
5.204	0.648	1.048	0.713	1	0.715	1	0.888	17.561	0.877
5.542	0.667	1.048	0.713	1	0.715	0.998	0.697	15.164	0.724
3.321	0.515	0.112	0.206	1	0.715	0.984	0.507	14.009	0.638
4.464	0.596	0.998	0.613	1	0.715	0.997	0.653	17.802	0.884
3.272	0.511	1.029	0.652	1	0.715	0.905	0.384	14.393	0.664
9.926	0.988	0.98	0.597	1	0.715	0.995	0.604	19.392	0.946
2.172	0.418	1.088	0.866	1	0.715	1	0.888	9.173	0.361
5.435	0.661	1.048	0.713	1	0.715	0.891	0.375	12.171	0.533
-1.948	0.016	-0.406	0.061	0	0.063	0.001	0.043	.	.
9.676	0.979	1.048	0.713	1	0.715	1	0.888	17.8	0.884
7.791	0.843	0.932	0.445	1	0.715	0.999	0.75	19.69	0.96
8.035	0.891	1.048	0.713	1	0.715	0.999	0.75	19.686	0.96
7.792	0.843	1.199	0.96	1	0.715	0.956	0.434	15.602	0.763
3.302	0.513	0.935	0.49	1	0.715	0.954	0.431	19.04	0.929
8.017	0.886	0.964	0.58	1	0.715	1	0.888	15.625	0.765
8.915	0.923	1.166	0.901	1	0.715	0.993	0.574	14.616	0.68
9.998	0.993	1.048	0.713	1	0.715	0.996	0.625	18.645	0.913
0.29	0.186	0.071	0.179	0.982	0.353	0.988	0.529	7.565	0.269
-0.483	0.066	-0.975	0.023	0	0.063	0	0.016	8.985	0.35
1.325	0.331	1.048	0.713	0.906	0.313	0.963	0.446	12.89	0.573
-1.143	0.033	-0.528	0.049	0.003	0.159	0.5	0.271	3.307	0.065
-2.15	0.014	-1.72	0.007	0.001	0.137	0.001	0.043	.	.

0.728	0.255	0.978	0.596	0.224	0.244	0.815	0.341	7.105	0.244
0.187	0.167	-0.487	0.053	0.001	0.137	0.035	0.152	11.234	0.48
2.089	0.411	0.807	0.329	1	0.715	1	0.888	11.042	0.469
-0.269	0.086	-0.653	0.041	0.862	0.304	0.003	0.074	1.173	0.017
-0.265	0.086	0.007	0.138	0.002	0.151	0.678	0.304	7.233	0.251
2.344	0.434	-0.013	0.131	1	0.715	0.96	0.441	13.298	0.596
4.996	0.634	1.011	0.635	1	0.715	0.999	0.75	12.474	0.55
8.34	0.899	1.048	0.713	1	0.715	0.955	0.433	20.071	0.977
0.114	0.152	-1.009	0.022	0.057	0.215	0	0.016	3.618	0.076
6.832	0.749	0.953	0.551	1	0.715	0.294	0.236	15.157	0.723
0.54	0.227	-0.346	0.071	0	0.063	0.001	0.043	2.283	0.038
6.164	0.715	0.935	0.49	1	0.715	1	0.888	19.033	0.929
1.042	0.297	0.87	0.379	0.926	0.319	0.772	0.328	13.105	0.585
9.184	0.94	0.802	0.324	1	0.715	0.012	0.117	13.647	0.616
0.023	0.133	0.095	0.197	0	0.063	0.54	0.278	7.308	0.255
4.212	0.581	0.852	0.362	1	0.715	0.806	0.338	14.806	0.695
2.203	0.421	0.935	0.49	0.949	0.328	0.052	0.166	13.078	0.583
2.747	0.469	0.833	0.339	0.996	0.391	0.998	0.697	13.092	0.584
4.142	0.576	1.199	0.96	1	0.715	0.123	0.197	11.148	0.475
6.353	0.728	1.088	0.866	1	0.715	0.991	0.552	16.055	0.805
9.457	0.968	0.879	0.385	1	0.715	0.669	0.302	13.825	0.627
6.044	0.704	0.935	0.49	1	0.715	0.96	0.441	15.389	0.743
0.844	0.271	0.739	0.307	0	0.063	0.015	0.124	5.042	0.137
1.84	0.386	0.927	0.437	1	0.715	0.996	0.625	13.902	0.632
3.298	0.513	0.017	0.142	0.234	0.245	0.053	0.166	10.117	0.416
6.167	0.716	0.892	0.403	1	0.715	0.809	0.339	13.397	0.601
0.079	0.145	0.902	0.416	0	0.063	0.031	0.148	14.215	0.652
1.085	0.302	0.117	0.209	0.002	0.151	0.99	0.544	7.372	0.258
5.198	0.648	0.97	0.593	0.905	0.313	0.023	0.138	15.43	0.747
1.862	0.389	0.892	0.403	0.83	0.299	0.783	0.331	5.951	0.184
-2.602	0.01	-0.212	0.1	0	0.063	0.002	0.062	1.266	0.019
2.369	0.436	-0.204	0.101	0.988	0.363	0.654	0.299	12.291	0.54
3.94	0.561	1.199	0.96	1	0.715	1	0.888	10.436	0.434

3.393	0.52	0.932	0.445	1	0.715	0.972	0.466	15.559	0.759
7.937	0.87	0.986	0.6	1	0.715	0.638	0.296	14.259	0.655
1.239	0.321	0.11	0.204	0.002	0.151	0.95	0.426	0.921	0.012
0.437	0.211	-0.602	0.044	0.045	0.21	0.51	0.273	13.296	0.596
3.458	0.525	0.191	0.249	1	0.715	0.986	0.517	8.197	0.304
0.41	0.207	0.935	0.49	0.991	0.37	1	0.888	8.565	0.325
5.056	0.639	0.935	0.49	1	0.715	0.861	0.359	19.154	0.935
4.547	0.602	0.852	0.362	1	0.715	0.965	0.45	18.513	0.908
6.194	0.718	1.14	0.893	0.942	0.325	0.052	0.166	9.966	0.407
0.663	0.246	-0.022	0.129	0	0.063	0	0.016	8.739	0.335
7.41	0.791	0.07	0.177	1	0.715	0.591	0.287	11.997	0.523
2.203	0.421	1.038	0.658	0.9	0.312	0.955	0.433	7.519	0.266
7.822	0.847	0.852	0.362	1	0.715	0.993	0.574	15.512	0.754
3.142	0.5	0.852	0.362	0.203	0.241	0.959	0.439	9.666	0.39
-1.632	0.021	-0.329	0.075	0	0.063	0.001	0.043	3.332	0.066
7.786	0.842	0.935	0.49	1	0.715	0.988	0.529	18.061	0.893
2.14	0.415	0.892	0.403	1	0.715	0.997	0.653	11.278	0.482
0.473	0.217	0.964	0.58	1	0.715	0.994	0.587	6.864	0.231
5.158	0.645	1.199	0.96	1	0.715	1	0.888	12.83	0.569
2.737	0.468	0.935	0.49	1	0.715	0.998	0.697	19.841	0.967
6.493	0.734	-0.083	0.118	0.986	0.359	0.123	0.197	9.395	0.374
2.296	0.43	0.839	0.342	0.943	0.325	0.074	0.178	15.541	0.757
0.076	0.145	0.065	0.171	0.328	0.253	0.956	0.434	10.316	0.427
4.3	0.586	0.935	0.49	0.999	0.424	0.999	0.75	19.575	0.954
-0.258	0.087	-3.053	0.001	0	0.063	0	0.016	.	.
0.099	0.149	1.13	0.883	0.012	0.185	0.109	0.192	.	.
3.755	0.547	0.892	0.403	0.998	0.411	0.996	0.625	19.436	0.948
2.048	0.407	0.148	0.236	0.99	0.367	0.983	0.502	8.264	0.308
-0.335	0.079	-0.055	0.122	0.024	0.198	0.189	0.215	7.096	0.243
7.032	0.761	0.949	0.536	1	0.715	0.849	0.354	16.003	0.8
0.569	0.232	-0.035	0.126	0.116	0.229	0.499	0.271	11.233	0.48
1.068	0.3	1.199	0.96	0.611	0.276	0.008	0.104	7.681	0.275
5.759	0.682	1.044	0.665	1	0.715	0.767	0.326	15.652	0.768

2.705	0.465	0.935	0.49	1	0.715	0.996	0.625	14.313	0.658
4.619	0.607	1.048	0.713	1	0.715	1	0.888	5.556	0.163
9.579	0.973	1.036	0.656	1	0.715	1	0.888	19.075	0.931
2.569	0.454	0.935	0.49	0.997	0.399	0.996	0.625	13.339	0.598
5.059	0.639	0.935	0.49	1	0.715	0.997	0.653	13.626	0.615
1.796	0.382	0.953	0.551	0.983	0.354	0.96	0.441	12.694	0.562
5.448	0.661	0.935	0.49	1	0.715	0.049	0.163	19.243	0.939
5.726	0.679	0.935	0.49	1	0.715	0.932	0.405	19.566	0.954
5.633	0.673	1.061	0.807	1	0.715	0.993	0.574	9.614	0.387
-0.009	0.126	0.964	0.58	0.063	0.217	0.251	0.228	8.684	0.332
1.856	0.388	0.075	0.182	0.767	0.291	0.257	0.229	6.693	0.222
5.012	0.635	1	0.621	1	0.715	0.987	0.523	18.27	0.9
4.837	0.623	1.199	0.96	0.522	0.269	0.853	0.356	11.551	0.498
-0.026	0.122	0.88	0.385	0	0.063	1	0.888	9.537	0.382
0.541	0.227	0.935	0.49	0	0.063	0.947	0.422	8.083	0.297
0.181	0.166	-2.425	0.003	0.003	0.159	0.016	0.126	6.101	0.191
1.981	0.4	0.935	0.49	0.98	0.35	0.997	0.653	5.807	0.176
1.918	0.394	1.024	0.645	1	0.715	0.998	0.697	8.21	0.305
5.368	0.657	0.892	0.403	1	0.715	1	0.888	13.536	0.609
-0.121	0.105	-0.586	0.045	0.686	0.283	0.087	0.184	6.119	0.192
-0.017	0.124	0.51	0.281	0.005	0.169	0.998	0.697	6.186	0.196
0.487	0.219	0.998	0.613	0	0.063	0.1	0.189	8.847	0.341
8.017	0.886	1.061	0.807	1	0.715	0.955	0.433	15.568	0.759
1.794	0.382	0.855	0.374	1	0.715	0.985	0.512	8.924	0.346
9.604	0.978	1.048	0.713	1	0.715	0.972	0.466	17.481	0.875
10.003	0.997	1.048	0.713	1	0.715	0.947	0.422	17.946	0.889
9.87	0.985	0.919	0.43	1	0.715	0.998	0.697	18.221	0.898
8.976	0.93	1.03	0.653	1	0.715	0.277	0.233	18.998	0.928
3.185	0.504	1.048	0.713	0.998	0.411	0.985	0.512	18.759	0.918
9.405	0.966	0.953	0.551	1	0.715	0.919	0.394	18.05	0.892
2.715	0.466	1.048	0.713	0.997	0.399	0.908	0.386	9.056	0.354
6.469	0.733	1.199	0.96	1	0.715	0.998	0.697	11.14	0.474
7.133	0.767	0.935	0.49	1	0.715	0.851	0.355	19.003	0.928

3.794	0.549	0.935	0.49	1	0.715	1	0.888	13.559	0.611
1.986	0.401	0.059	0.165	0.998	0.411	0.655	0.299	12.738	0.564
6.352	0.728	0.805	0.326	1	0.715	0.994	0.587	15.435	0.748
5.494	0.664	0.852	0.362	1	0.715	0.113	0.194	19.095	0.932
7.674	0.83	1.061	0.807	1	0.715	1	0.888	15.351	0.74
4.171	0.578	0.072	0.18	0.996	0.391	0.038	0.155	16.482	0.839
0.217	0.173	-0.298	0.081	0.954	0.33	0.689	0.307	4.366	0.106
0.234	0.176	0.098	0.198	0.961	0.334	0.948	0.423	1.031	0.014
4.213	0.581	0.076	0.183	1	0.715	0.999	0.75	11.647	0.503
1.793	0.382	0.935	0.49	0.856	0.303	0.909	0.387	9.028	0.352
-0.541	0.061	0.148	0.236	0.192	0.24	0.997	0.653	1.202	0.018
0.21	0.171	0.144	0.231	0	0.063	0.002	0.062	7.34	0.256
3.339	0.516	0.964	0.58	0.961	0.334	0.964	0.448	7.413	0.26
4.978	0.633	1.048	0.713	1	0.715	1	0.888	19.115	0.933
4.294	0.586	0.848	0.348	0.267	0.248	0.123	0.197	10.825	0.456
8.014	0.882	1.056	0.757	1	0.715	1	0.888	16.445	0.837
1.804	0.383	0.051	0.162	0.927	0.319	0.844	0.352	13.266	0.594
0.123	0.154	-0.084	0.118	0.001	0.137	0.001	0.043	2.025	0.033
8.012	0.881	1.061	0.807	1	0.715	0.982	0.498	15.152	0.723
1.116	0.306	0.935	0.49	0.993	0.376	0.962	0.444	11.569	0.499
1.105	0.305	0.07	0.177	0.001	0.137	0.005	0.09	8.869	0.343
1.284	0.326	0.953	0.551	0.628	0.277	0.991	0.552	14.571	0.677
0.729	0.255	0.953	0.551	0.005	0.169	0.18	0.213	8.2	0.304
2.463	0.445	0.935	0.49	0.983	0.354	0.001	0.043	4.648	0.119
3.872	0.556	0.892	0.403	1	0.715	0.994	0.587	7.404	0.26
6.104	0.71	1.048	0.713	1	0.715	0.342	0.244	15.671	0.769
3.881	0.556	0.979	0.596	0.985	0.357	0.013	0.12	13.569	0.611
-0.047	0.118	-0.053	0.122	0	0.063	0.001	0.043	6.733	0.224
2.205	0.422	0.935	0.49	0.999	0.424	0.963	0.446	12.764	0.566
7.174	0.77	0.892	0.403	1	0.715	0.991	0.552	12.932	0.575
1.433	0.343	0.789	0.32	0.027	0.2	0.023	0.138	10.996	0.466
-0.007	0.126	-0.349	0.07	0	0.063	0.001	0.043	2.476	0.043
7.682	0.833	1.199	0.96	1	0.715	0.634	0.295	13.865	0.629

1.419	0.342	0.839	0.342	0.892	0.31	0.427	0.259	9.046	0.353
5.324	0.654	1.048	0.713	1	0.715	0.994	0.587	18.551	0.91
2.357	0.435	1.088	0.866	0.025	0.199	0.998	0.697	14.455	0.668
3.172	0.503	1.026	0.645	0.837	0.3	0.685	0.306	12.311	0.541
1.902	0.392	0.227	0.256	0.006	0.172	0.977	0.48	7.545	0.268
-0.271	0.085	-0.33	0.074	0.937	0.323	0.646	0.298	3.588	0.075
7.781	0.842	0.892	0.403	1	0.715	0.998	0.697	14.864	0.699
0.946	0.285	1.056	0.757	0.983	0.354	0.959	0.439	9.329	0.37
0.846	0.271	-0.097	0.117	0.094	0.225	0.165	0.209	4.103	0.094
0.31	0.19	0.065	0.171	0.005	0.169	0.013	0.12	5.026	0.136
0.397	0.205	1.112	0.879	0.007	0.175	0.856	0.357	10.34	0.429
9.915	0.987	1.036	0.656	1	0.715	0.944	0.418	18.572	0.911
0.802	0.265	0.934	0.45	0.185	0.239	0.996	0.625	13.518	0.608
4.661	0.61	0.953	0.551	1	0.715	0.837	0.349	18.794	0.919
1.679	0.37	-0.073	0.12	0.021	0.196	0.385	0.252	7.799	0.282
7.196	0.772	0.949	0.536	1	0.715	0.995	0.604	16.854	0.858
8.198	0.896	1.194	0.916	1	0.715	0.998	0.697	16.609	0.846
7.66	0.826	0.935	0.49	1	0.715	0.954	0.431	18.576	0.911
2.736	0.468	-0.349	0.07	0.179	0.238	0	0.016	3.833	0.083
9.569	0.973	0.953	0.551	1	0.715	0.989	0.536	17.287	0.87
0.416	0.208	-0.512	0.051	0.165	0.236	0.93	0.404	6.166	0.195
7.519	0.806	1.048	0.713	1	0.715	0.924	0.398	16.427	0.836
0.017	0.132	0.154	0.24	0.91	0.314	0.993	0.574	4.681	0.12
0.455	0.214	0.851	0.35	0	0.063	0.048	0.163	3.864	0.085
4.536	0.601	0.935	0.49	1	0.715	0.969	0.458	15.437	0.748
0.972	0.288	0.631	0.29	0.007	0.175	0.868	0.363	9.575	0.384
1.35	0.334	0.835	0.34	0.13	0.231	0.837	0.349	7.868	0.285
0.386	0.203	0.852	0.362	0.001	0.137	0.247	0.227	15.108	0.719
0.243	0.177	-0.039	0.125	0.076	0.22	0.002	0.062	.	.
2.206	0.422	0.821	0.336	1	0.715	0.997	0.653	9.344	0.371
-4.901	0.002	-2.377	0.003	0	0.063	0	0.016	2.171	0.036
1.533	0.354	0.728	0.305	1	0.715	0.986	0.517	15.628	0.765
1.572	0.358	1.14	0.893	0.993	0.376	0.506	0.272	8.59	0.327

0.646	0.243	-0.021	0.129	0.023	0.197	0.011	0.114	10.233	0.423
1.593	0.361	0.361	0.273	0.66	0.28	0.007	0.1	5.87	0.179
2.839	0.477	0.853	0.373	1	0.715	0.511	0.273	12.433	0.547
0.053	0.14	0.845	0.345	0.001	0.137	0.766	0.326	5.864	0.179
-0.987	0.038	0.77	0.312	0	0.063	0.263	0.23	5.805	0.176
-0.962	0.039	-0.155	0.108	0	0.063	0.003	0.074	3.968	0.089
-0.708	0.051	-0.048	0.123	0	0.063	0	0.016	6.594	0.217
2.087	0.41	0.976	0.595	0.018	0.193	1	0.888	5.869	0.179
0.738	0.256	0.064	0.169	0.144	0.233	0.002	0.062	3.878	0.085
2.478	0.446	0.902	0.416	0.998	0.411	0.966	0.452	12.063	0.527
1.637	0.365	1.048	0.713	0.551	0.271	0.998	0.697	12.908	0.574
7.497	0.802	1.048	0.713	1	0.715	1	0.888	18.827	0.921
9.353	0.965	1.048	0.713	1	0.715	0.631	0.295	18.656	0.914
7.533	0.807	1.048	0.713	1	0.715	1	0.888	16.221	0.82
2.147	0.416	0.919	0.43	0.298	0.251	0.986	0.517	3.586	0.075
3.08	0.496	1.024	0.645	0.035	0.205	0.029	0.146	14.036	0.64
2.699	0.465	-0.443	0.057	0.262	0.247	0.003	0.074	6.387	0.206
0.918	0.281	0.935	0.49	0	0.063	0.024	0.14	7.844	0.284
2.226	0.424	0.998	0.613	0.038	0.207	0.978	0.483	13.179	0.589
2.243	0.425	0.954	0.563	1	0.715	0.991	0.552	13.305	0.596
4.96	0.631	1.045	0.669	1	0.715	0.997	0.653	19.835	0.966
3.853	0.554	0.852	0.362	1	0.715	0.994	0.587	11.304	0.484
0.121	0.154	0.077	0.185	0.858	0.303	0.999	0.75	5.437	0.157
1.915	0.394	0.935	0.49	1	0.715	0.997	0.653	16.524	0.841
2.228	0.424	0.937	0.529	1	0.715	0.993	0.574	12.027	0.525
6.125	0.712	0.935	0.49	1	0.715	1	0.888	20.344	0.988
7.793	0.843	0.998	0.613	1	0.715	0.325	0.241	12.115	0.53
4.494	0.598	0.62	0.289	1	0.715	0.032	0.149	14.32	0.659
5.957	0.698	0.701	0.299	1	0.715	0.933	0.406	11.476	0.494
4.906	0.628	0.557	0.284	1	0.715	0.913	0.39	14.531	0.674

Interpro_don	GTEx_V6_ge	GTEx_V6_tissue
Arfaptin hom	.	.
GPCR family	.	.
UBX domain,	.	.
UBX domain	ENSG000001	Lung
.	.	.
.	.	.
.	.	.
Zinc finger C	.	.
P-loop contai	.	.
.	.	.
.	.	.
Metal-depen	.	.
.	.	.
.	.	.
.	.	.
Heat shock c	.	.
Ribosomal pr	.	.
GPCR, rhodo	.	.
.	.	.
.	.	.
.	.	.
.	.	.
Protein kinas	.	.
.	.	.
AAA+ ATPase	.	.
.	.	.
.	.	.
Zinc finger C	.	.
GPCR, rhodo	.	.
GPCR, rhodo	.	.
.	.	.
.	.	.

.	.	.
Activator of I	.	.
.	.	.
C2 domain	.	.
.	.	.
Concanavalir	.	.
.	.	.
.	.	.
Ion transport	.	.
.	.	.
.	.	.
Double-stran	.	.
Immunoglob	.	.
.	.	.
Quinoproteir	.	.
P-loop contai	.	.
E3 ubiquitin-	.	.
.	.	.
Nucleic acid-	.	.
.	.	.
.	.	.
Tetratricoper	.	.
SCA7 domair	.	.
.	.	.
Ephrin recep	.	.
ATPase, F1/V	.	.
Amino acid p	.	.
Diacylglycerc	.	.
Protein kinas	.	.
.	ENSG000002	Thyroid
Transferrin-li	.	.
Krueppel-ass	.	.
.	.	.

.	.	.
.	.	.
Alpha-2-mac	.	.
.	.	.
.	.	.
.	.	.
DBB domain	.	.
High mobility	.	.
.	.	.
Leucine-rich	.	.
.	.	.
Sorting nexir	.	.
PH domain-li	.	.
Cadherin|Ca	.	.
Cadherin, cyt	.	.
Sterile alpha	.	.
GPCR, rhodo	.	.
.	.	.
.	.	.
.	.	.
B30.2/SPRY	.	.
.	.	.
.	.	.
.	.	.
Aminoacyl-tF	.	.
Heat shock p	.	.
Fibronectin t	.	.
.	.	.
.	.	.
.	.	.
.	.	.
AAA+ ATPase	.	.
.	.	.

.	.	.
Myc-type, ba	.	.
PKD domain	.	.
GPCR, rhodo	.	.
.	.	.
Peptidase M	.	.
Peptidase M	.	.
.	.	.
Ankyrin repe	.	.
.	.	.
Doublecortin	.	.
Macrophage	.	.
.	.	.
Soluble quin	.	.
.	.	.
Prokineticin	.	.
.	.	.
.	.	.
Sushi/SCR/C	.	.
.	.	.
DNA topoiso	.	.
Armadillo-lik	.	.
.	.	.
PDZ domain	.	.
.	ENSG000001	Adipose_Subcutaneous|Colon_Transverse|Muscle_Skeletal|Nerve_Tibial|Skin_Not_Sun_Exposed_Suprap
.	.	.
.	.	.
WD40-repea	.	.
.	.	.
Concanavalir	.	.
Adenosine de	.	.
.	.	.
.	.	.

Zinc finger, C	.	.
Storkhead-bo	.	.
.	.	.
Zinc finger, L	.	.
Polycystin ca	.	.
.	.	.
.	.	.
Potassium ch	.	.
.	.	.
Leucine-rich	.	.
Insulin-like	.	.
GPCR, rhodo	.	.
.	.	.
Zinc finger C	.	.
.	.	.
Ribosomal pr	.	.
P-loop contai	.	.
Chaperone D	.	.
Domain of un	.	.
.	.	.
.	.	.
GPCR, rhodo	.	.
Metallopepti	.	.
.	.	.
Glycoside hy	.	.
.	.	.
GPCR, rhodo	.	.
S-adenosyl-L	.	.
.	.	.
STAT transcr	.	.
Myosin head	.	.
.	.	.
Green fluore	.	.

.	.	.
Tryptophan s	.	.
Glycoside hy	.	.
PH domain-li	.	.
.	.	.
.	.	.
PPM-type ph	.	.
.	.	.
.	.	.
Chromograni	.	.
.	.	.
.	.	.
Immunoglob	.	.
.	.	.
.	.	.
Nucleotide-b	.	.
.	.	.
.	.	.
.	.	.
Armadillo-lik	.	.
Concanavalir	.	.
.	.	.
.	.	.
.	.	.
Protein kinas	.	.
.	.	.
.	.	.
.	.	.
.	.	.
C2 domain|S	.	.
.	.	.
Integrin alph	.	.
.	.	.

.	.	.
.	.	.
SH2 domain	.	.
.	.	.
FIIND domai	.	.
.	.	.
.	.	.
Glutathione-	.	.
.	.	.
.	.	.
.	.	.
AAA+ ATPas	.	.
Immunoglob	.	.
KEN domain	.	.
.	.	.
.	.	.
.	.	.
.	.	.
.	.	.
GPCR, rhodo	.	.
GPCR, rhodo	.	.
Ankyrin repe	.	.
Cadherin|Ca	.	.
.	.	.
Fibronectin t	.	.
.	.	.
.	.	.
.	.	.
C-type lectin	.	.
.	.	.
.	.	.
TRAM/LAG1,	.	.
.	.	.

.	.	.
Concanavalir	.	.
.	.	.
.	.	.
Zinc finger C	ENSG000001	Cells_Transformed_fibroblasts|Whole_Blood
.	.	.
.	.	.
Zinc finger C	.	.
.	.	.
.	.	.
Transglutam	.	.
.	.	.
.	.	.
.	.	.
Tumour-supp	.	.
AAA+ ATPase	.	.
.	.	.
P-loop contai	.	.
.	.	.
UBA-like|Ub	.	.
.	.	.
.	.	.
.	.	.
.	.	.
.	.	.
.	.	.
Plexin, cytopl	.	.
Alkaline phos	.	.
Alkaline phos	.	.
Alkaline phos	.	.

ıubic|Skin_Sun_Exposed_Lower_leg|Thyroid
